# Plasma metabolomic profiling in subclinical atherosclerosis: the Diabetes Heart Study

**DOI:** 10.1186/s12933-021-01419-y

**Published:** 2021-12-07

**Authors:** Parag Anilkumar Chevli, Barry I. Freedman, Fang-Chi Hsu, Jianzhao Xu, Megan E. Rudock, Lijun Ma, John S. Parks, Nicholette D. Palmer, Michael D. Shapiro

**Affiliations:** 1 Section on Hospital Medicine, Department of Internal Medicine, Wake Forest School of Medicine, Winston-Salem, NC USA; 2 Section on Nephrology, Department of Internal Medicine, Wake Forest School of Medicine, Winston-Salem, NC USA; 3 Department of Biostatistics and Data Science, Division of Public Health Sciences, Wake Forest School of Medicine, Winston-Salem, NC USA; 4 Department of Biochemistry, Wake Forest School of Medicine, 1 Medical Center Blvd, Winston-Salem, NC 27157 USA; 5 Section on Molecular Medicine, Department of Internal Medicine, Wake Forest School of Medicine, Winston-Salem, NC USA; 6 Section of Cardiovascular Medicine, Center for Preventive Cardiology, Wake Forest School of Medicine, 1 Medical Center Blvd, Winston-Salem, NC 27157 USA

**Keywords:** Diabetes mellitus, Cardiovascular disease, Coronary artery calcium, Metabolomics, African Americans, European Americans

## Abstract

**Background:**

Incidence rates of cardiovascular disease (CVD) are increasing, partly driven by the diabetes epidemic. Novel prediction tools and modifiable treatment targets are needed to enhance risk assessment and management. Plasma metabolite associations with subclinical atherosclerosis were investigated in the Diabetes Heart Study (DHS), a cohort enriched for type 2 diabetes (T2D).

**Methods:**

The analysis included 700 DHS participants, 438 African Americans (AAs), and 262 European Americans (EAs), in whom coronary artery calcium (CAC) was assessed using ECG-gated computed tomography. Plasma metabolomics using liquid chromatography-mass spectrometry identified 853 known metabolites. An ancestry-specific marginal model incorporating generalized estimating equations examined associations between metabolites and CAC (log-transformed (CAC + 1) as outcome measure). Models were adjusted for age, sex, BMI, diabetes duration, date of plasma collection, time between plasma collection and CT exam, low-density lipoprotein cholesterol (LDL-C), and statin use.

**Results:**

At an FDR-corrected p-value < 0.05, 33 metabolites were associated with CAC in AAs and 36 in EAs. The androgenic steroids, fatty acid, phosphatidylcholine, and bile acid metabolism subpathways were associated with CAC in AAs, whereas fatty acid, lysoplasmalogen, and branched-chain amino acid (BCAA) subpathways were associated with CAC in EAs.

**Conclusions:**

Strikingly different metabolic signatures were associated with subclinical coronary atherosclerosis in AA and EA DHS participants.

**Supplementary Information:**

The online version contains supplementary material available at 10.1186/s12933-021-01419-y.

## Background

Despite four decades of declines in cardiovascular disease (CVD) mortality, CVD continues to be the leading cause of death and disability in the United States and globally [1]. Current projections forecast an increase in CVD, primarily driven by the epidemic of type 2 diabetes (T2D). While the increased risk of CVD associated with diabetes is multifactorial, it is clear that altered lipid, branched-chain amino acid, and carbohydrate metabolism likely mediate some of the risk [2–4]. There is considerable interest in the various “omics” technologies as a means to investigate the molecular underpinnings of atherosclerosis. Metabolomics has garnered significant attention due to its advantages relative to other measures, providing a direct reflection of biochemical activity and the underlying state of the organism. It is anticipated that insights from metabolomics will translate into new CVD biomarkers and potentially novel therapeutic targets. The American Heart Association recently recognized the growing importance of metabolomics on risk for CVD [5].

The burden of coronary artery atherosclerosis effectively predicts CVD events. Quantification of coronary artery calcium (CAC) with electrocardiographic gated computed tomography is a non-invasive method for determining subclinical atherosclerosis and is widely recommended for risk assessment [6–9]. Although the prevalence and severity of CAC vary based on ancestry, significant associations exist between CAC and CVD mortality irrespective of race/ethnicity in the CAC Consortium. These data include the largest racially heterogeneous cohort of CAC scores assembled [10]. Similarly, CAC predicts CVD and all-cause mortality in individuals with diabetes [11].

Few studies have examined the association between metabolomic profiling and subclinical atherosclerosis, and results have been inconsistent [12–14]. A recent study of 209 Japanese subjects with T2D demonstrated that there were statistically significant associations between the brachial-ankle pulse wave velocity and plasma levels of indoxyl sulfate, mannitol, mesoerythritol, and pyroglutamic acid [15]. Moreover, very few studies have assessed metabolic signatures associated with CVD in those with diabetes. A study only examining plasma lipidomic profiles demonstrated the potential of plasma lipid species as biomarkers for cardiovascular risk stratification in T2D [16]. Given the dearth of evidence and the profound metabolic changes associated with diabetes, we aimed to identify metabolic pathways associated with subclinical atherosclerosis in individuals with T2D using targeted plasma metabolomic profiling.

## Methods

The data that support the findings of this study are available from the corresponding author upon reasonable request.

### Study participants

Eligibility and recruitment methods in the Diabetes Heart Study (DHS) have been reported previously [17]. In brief, DHS is a community-based study of the epidemiologic and genetic contributions to CVD in the setting of diabetes. It enrolled siblings concordant for T2D (without advanced kidney disease) and an unaffected family member. T2D was defined as a clinical diagnosis of diabetes after the age of 34 years, in the absence of historical evidence of diabetic ketoacidosis. Participant examinations were conducted in the General Clinical Research Center (GCRC) of Wake Forest University School of Medicine (WFSM). Examinations included interviews for medical history and health behaviors, anthropometric measures, resting blood pressure, electrocardiography, fasting blood sampling, and a spot urine collection. Hypertension was defined as a physician reported diagnosis, use of anti-hypertensive medications, or measured clinic blood pressure > 140/90 mm Hg. Diabetes duration was self-reported. CVD was defined by participants reporting history of a CVD event including MI, angina, or stroke; history of vascular procedures including coronary angioplasty, coronary artery bypass graft, or endarterectomy; or with Q wave abnormalities on ECG indicative of prior MI. Laboratory assays included urine albumin and creatinine, total cholesterol, low-density lipoprotein (LDL)-cholesterol (LDL-C), high-density lipoprotein (HDL)-cholesterol (HDL-C), triglycerides, glycated hemoglobin, fasting glucose, and blood chemistries. The WFSM Institutional Review Board approved the protocol, and all participants provided written informed consent.

### Coronary Artery Calcium

Coronary artery calcium (CAC) was quantified using a standardized protocol based on those implemented in the National Heart, Lung, and Blood Institute’s (NHLBI) Coronary Artery Risk Development in Young Adults (CARDIA) and Multi-Ethnic Study of Atherosclerosis (MESA) studies [18]. The scanning technique and quantification of CAC in the DHS utilized single-and multi-detector computed tomography (CT) systems, as previously reported [19]. Two experienced cardiac imagers quantified CAC with the SmartScore software package (GE Advantage Windows). Imaging measures included the Agatston score corrected for slice thickness, calcified plaque mass, and calcified plaque volume. All calcified plaques were quantified by a more sensitive CT attenuation threshold of 90 Hounsfield units (HU) [11].

### Metabolomics analysis

Global untargeted metabolomics profiling of plasma samples was performed by Metabolon Inc. (Durham, North Carolina) on the DiscoveryHD4 panel. Fasting plasma samples were stored at − 80 °C since collection between 1998 and 2010. Samples were prepared using the automated MicroLab STAR system (Hamilton Company, Salt Lake City, UT). A methanol extraction was used to remove protein, dissociate small molecules bound to protein or trapped in the precipitated protein matrix, and to recover chemically diverse metabolites. The resulting extract was divided into five fractions: two for analysis by two separate reverse phase/ultra-performance liquid chromatography-mass spectroscopy (MS)/MS methods with positive ion mode electrospray ionization (ESI), one for analysis by reverse phase/ultra-performance liquid chromatography-MS/MS with negative ion mode ESI, one for analysis by hydrophilic interaction liquid chromatography/ultra-performance liquid chromatography-MS/MS with negative ion mode ESI and one sample was reserved for backup. All methods utilized a Waters ACQUITY ultra-performance liquid chromatography (UPLC) and a Thermo Scientific Q-Exactive high resolution/accurate mass spectrometer interfaced with a heated electrospray ionization source and Orbitrap mass analyzer operated at 35,000 mass resolution. Raw data were extracted, peak-identified and quality control processed using Metabolon’s hardware and software. Compounds were identified by comparison to library entries of purified standards or recurrent unknown entities. Peaks were quantified using area under the curve. Several types of controls were analyzed in addition to experimental samples: a technical replicate, pooled matrix sample generated from a small volume of each experimental sample; process blanks, extracted water samples; and QC standards, a cocktail of QC standards chosen not to interfere with the measurement of endogenous compounds were spiked into every analyzed sample, allowed instrument performance monitoring and aided chromatographic alignment. This panel identified and provided relative quantification of known chemical compounds (n = 853) among amino acid, carbohydrate, energy, lipid, nucleotide, and peptide super pathways. In addition to individual named biochemicals; super- and sub-pathways were annotated based on a combination of pathway and chemical structure similarities to serve as a guide for interpretation. Prior to return, data were block corrected for a run day, normalized by batch, and volume extracted. Missing data for metabolites were imputed to the minimum.

### Statistical analysis

Mean, and standard deviation (SD) were computed for continuous, normally distributed variables. Median, first quartile, third quartile, and interquartile range (IQR) were also computed for continuous, non-normally distributed variables. Count and percentage were computed for discrete variables by race/ethnicity groups. The comparisons between race/ethnicity groups were performed using marginal models incorporating generalized estimating equations (GEEs). To study the associations between metabolites and CAC, ancestry-specific linear regression models incorporating GEEs were performed. These models account for familial correlation using a sandwich estimator of the variance. An exchangeable correlation structure was used. Metabolites were modeled as predictors, with CAC as the outcome. Metabolites were covariates of interest and inverse normal transformed. For metabolites not detected in more than 50% of participants (AAs: n = 181; EAs: n = 167), they were analyzed as both continuous and binary measures (dichotomized as detectable vs. non-detectable). CAC was log-transformed to satisfy the conditional normality assumption. It is possible to have a CAC score of 0. In order to have meaningful log-transformed values, a constant of one was added to CAC before the log transformation (log (CAC + 1)). For participants who had prior procedures, including coronary angioplasty, coronary artery bypass graft, or endarterectomy, we winsorized their CAC scores based on the 95th percentile. We also analyzed data using dichotomized CAC (presence (CAC ≥ 10) vs. absence (CAC < 10)) [20]. As a conservative approach accounting for the evolution of CT scanners used in this study, absolute dichotomous calling of CAC reflecting presence and absence was avoided as participants with extremely low scores on original equipment were felt to lack detectable CAC. Thus, we report CAC as present when ≥ 10, based upon our previous publication, supporting statistical validity and robustness of this approach [11]. We fit a series of models. The first model was the basic model controlling for age, sex, body mass index (BMI), smoking status, hypertension status, CVD, diabetes duration, date of plasma collection, and time between plasma collection and CT exam. The second model additionally adjusted for LDL-C. The third model included all covariates in the first model but further adjusted for LDL-C and statin use. A false discovery rate (FDR) was used in consideration of multiple testing. A mediation analysis was performed comparing the regression coefficients in the fully adjusted model with and without duration of diabetes as a covariate to assess the impact of diabetes duration. We considered a change in estimate of 20% or greater to be meaningful mediation. Cross-ancestry replication used a nominally significant threshold, P < 0.05, to indicate statistical significance. Additional DHS participant samples, recruited and processed in identical fashion, were evaluated to assess replication of metabolite associations with CAC. A nominally significant threshold, P < 0.05, was used to evaluate statistical significance.

## Results

### Baseline characteristics

The study sample included 700 participants, 438 AAs from 334 families (22 non-diabetic and 416 with T2D) and 262 EAs from 204 families (40 non-diabetic and 222 with T2D). Table 1 contains baseline characteristics; 55.3% of participants were women, and moderate obesity was common. Consistent with enrichment for T2D, the study population had mean ages of 58.7 and 61.8 years in AAs and EAs, respectively; mean diabetes duration exceeded 10 years in both groups. As expected, EAs had a higher median CAC score compared with AAs (493.5 for EAs vs. 321 for AAs, p = 0.010).

**Table 1:** Baseline characteristics of DHS participants

Characteristic	African American		European American		*P*-value^a^
	N	Mean ± SD or N (%)	N	Mean ± SD or N (%)	
Male, N (%)	438	183 (41.8)	262	130 (49.6)	
Age (years)	438	58.7 ± 8.8	262	61.8 ± 9.0	** < 0.001**
Education, N (%)	431		259		
Less Than High School		64 (14.9)		57 (22.0)	
High School Graduated		231 (53.6)		138 (53.3)	
Above High School		136 (31.5)		64 (24.7)	
Smoking Status, N (%)	435		261		**0.005**
Never		150 (34.5)		109 (41.8)	
Former		173 (39.8)		110 (42.1)	
Current		112 (25.7)		42 (16.1)	
BMI (kg/m^2^)	437	34.0 ± 7.8	262	31.8 ± 6.4	** < 0.001**
Total Cholesterol (mg/dL)	428	180.0 ± 42.5	256	186.4 ± 41.8	0.069
LDL-C (mg/dL)	419	106.5 ± 34.8	240	106.6 ± 34.9	0.986
HDL-C (mg/dL)	428	48.9 ± 13.8	256	43.0 ± 12.4	** < 0.001**
Triglyceride^b^ (mg/dL)	428	98 (75, 145)	256	171.5 (123, 235)	** < 0.001**
Creatinine (mg/dL)	428	1.05 ± 0.32	262	1.11 ± 0.30	**0.002**
Systolic Blood Pressure (mm Hg)	438	137.4 ± 19.6	262	140.3 ± 18.0	**0.037**
Diastolic Blood Pressure (mm Hg)	438	75.7 ± 11.3	262	73.3 ± 9.8	**0.002**
Hypertension, N (%)	438	374 (85.4)	262	231 (88.2)	0.329
Diabetes duration (years)	415	10.8 ± 7.8	220	10.4 ± 6.9	0.811
Fasting glucose (mg/dL)	438	149.8 ± 71.8	262	145.4 ± 57.8	0.370
HbA1C (%)	430	8.2 ± 2.2	260	7.4 ± 1.7	** < 0.001**
CVD, N (%)	304	98 (32.2)	125	51 (40.8)	0.329
CAC^b^	433	321 (5.5, 2186.5)	250	493.5 (37.5, 6284.5)	**0.010**

*P*-values marked with bold indicate statistically significant differences between the groups

*BMI* Body Mass Index, *CAC* Coronary artery calcium, *CVD* Cardiovascular disease, *DHS* Diabetes Heart Study, *HDL-C* High-density lipoprotein cholesterol, *LDL-C* Low-density lipoprotein cholesterol

^a^P-value by a marginal model with generalized estimating equations

^b^Median (Interquartile range)

### CAC Association among African Americans

**Fig. 1:**
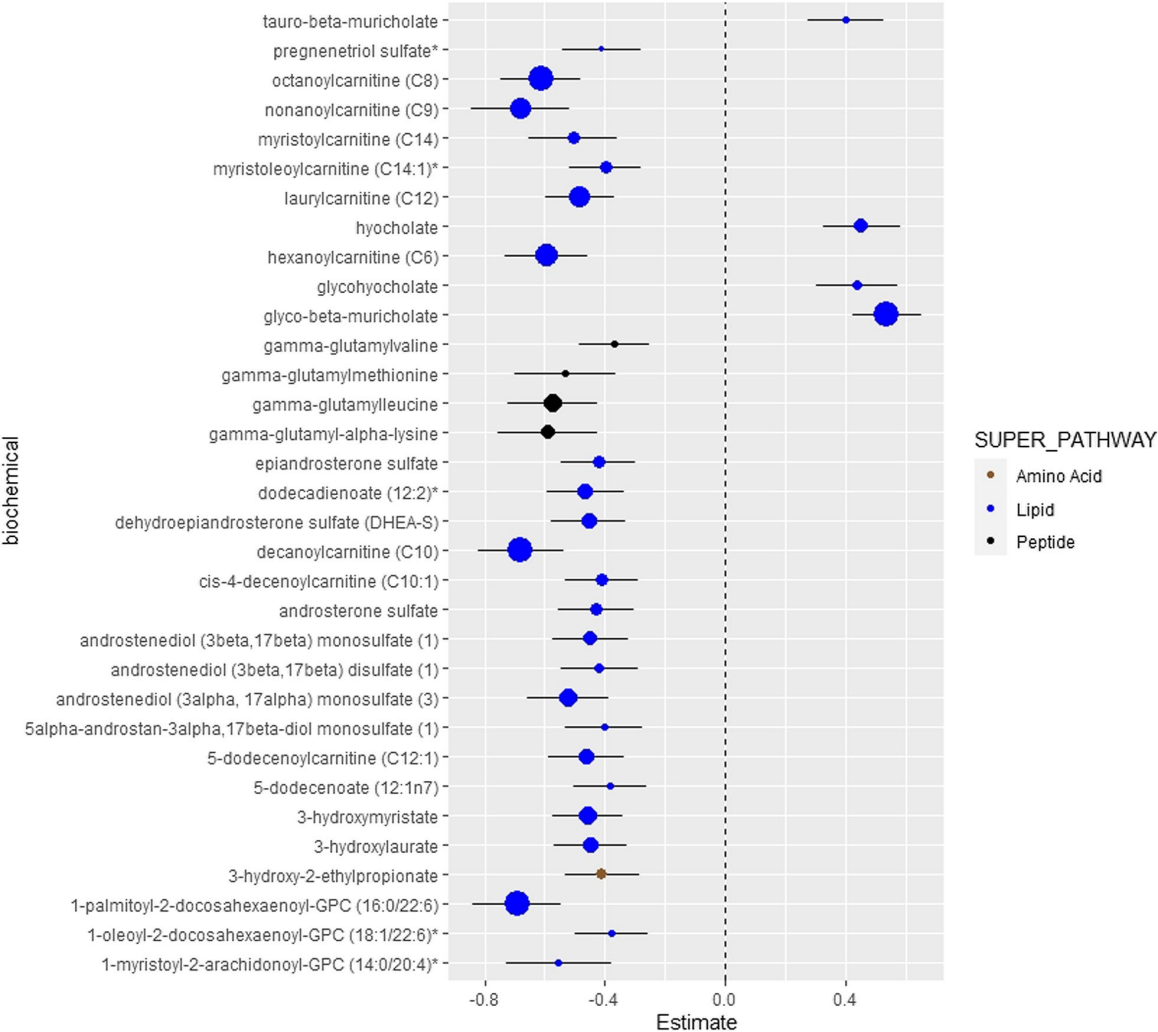
Association between plasma metabolites and CAC in AA. The model was adjusted for age, sex, BMI, smoking status, hypertension status, CVD, duration of diabetes, date of plasma collection, time between plasma collection and CT exam, LDL-C, statin use. Data are sorted by super pathway, sub pathway, and biochemical. Regression coefficients per standard deviation (95% confidence interval) of metabolites associated with coronary artery calcium. Data point size corresponds to statistical significance on a –log_10_ (P_FDR_) scale. *Indicates compounds that have not been officially confirmed based on a standard, but identified by virtue of their recurrent chromatographic and spectral nature

A total of 853 metabolites were analyzed (Additional file 1: Table S1). Metabolites not detected in more than 95% of participants were removed (AAs: n = 104; EAs: n = 123) from the analyses. Among AAs, 22 metabolites were statistically significantly associated with CAC after adjustment for age, sex, BMI, smoking status, hypertension status, CVD, diabetes duration, date of plasma collection, time between plasma collection and CT exam (FDR-corrected p-value < 0.05; Additional file 1: Table S2). 17 out of 22 metabolites were derived from the lipid metabolism superpathway. The androgenic steroid and fatty acid metabolism subpathways were inversely associated with CAC, whereas primary and secondary bile acid metabolism subpathway was positively associated with CAC in AAs. When the model was further adjusted for LDL-C, in addition to the covariates in the initial model, there were 36 metabolites associated with CAC (Additional file 1: Table S3). The majority of metabolites (31 metabolites) were derived from lipid metabolism pathways. These lipid metabolism metabolites were derived from androgenic steroid, acylcarnitine (medium-chain and monounsaturated), dicarboxylate fatty acid, monohydroxy fatty acid, medium-chain fatty acid, pregnenolone steroids, primary/secondary bile acid, and phosphatidylcholine subpathways. In the final model, further adjusted for statin use, in addition to LDL-C and covariates in the initial model, 33 total metabolites were associated with CAC in AAs, including 28 metabolites from the lipid metabolism pathway (Fig. 1). As in the prior analysis, the androgenic steroid and fatty acid metabolism subpathways demonstrated an inverse association with CAC, while the bile acid metabolism subpathway demonstrated a positive association with CAC (Additional file 1: Table S4). Table 2 displays the lipid metabolites significantly associated with CAC among AAs in the three different models. Results from the mediation analysis (Additional file 1: Table S14) did not suggest that duration of diabetes significantly impacted these findings. In addition, the impact of HbA1c, as an alternative to diabetes duration, was explored in the fully-adjusted model, and the results were not significantly different. (Additional file 1: Table S8).

**Table 2:** Number of Lipid Metabolites associated with CAC in DHS participants

Adjustment of Co-variates	African American	European American
Initial^a^	17	7
Initial^a^ + LDL-C	31	30
Initial^a^ + LDL-C + Statin	28	19

*CAC* Coronary artery calcium, *DHS* Diabetes Heart Study, *LDL-C* Low-density lipoprotein cholesterol

^a^Initial adjustments include age, sex, BMI, smoking status, hypertension status, CVD, duration of diabetes, date of plasma collection, and time between plasma collection and CT exam

### CAC Association among European Americans

**Fig. 2:**
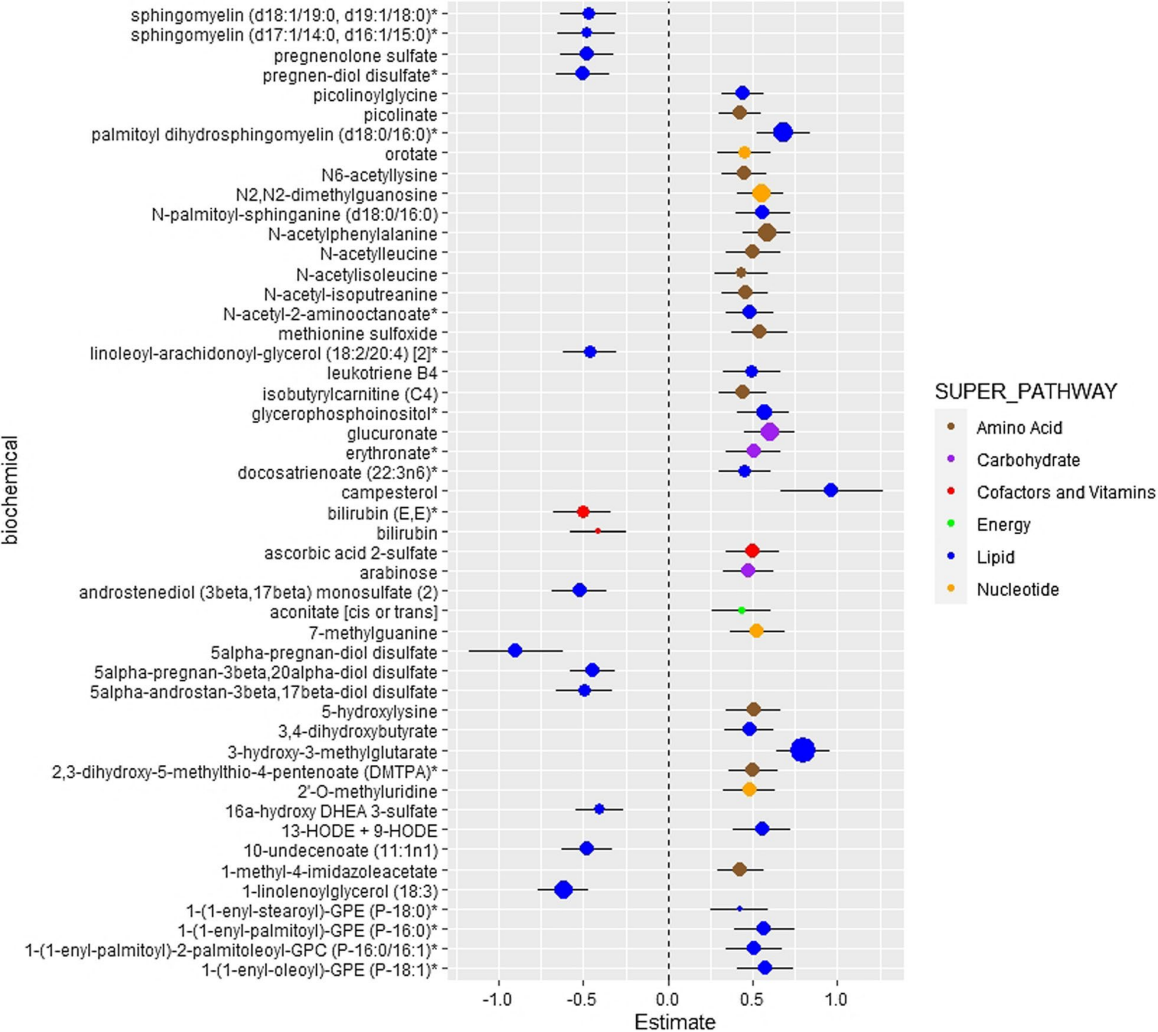
Association between plasma metabolites and CAC in EA. The model was adjusted for age, sex, BMI, smoking status, hypertension status, CVD, duration of diabetes, date of plasma collection, time between plasma collection and CT exam, LDL-C, statin use. Data are sorted by super pathway, sub pathway, and biochemical. Regression coefficients per standard deviation (95% confidence interval) of metabolites associated with coronary artery calcium. Data point size corresponds to statistical significance on a –log_10_ (P_FDR_) scale. *Indicates compounds that have not been officially confirmed based on a standard, but identified by virtue of their recurrent chromatographic and spectral nature

Adjusting for age, sex, BMI, smoking status, hypertension status, CVD, diabetes duration, date of plasma collection, time between plasma collection and CT exam, 12 metabolites were significantly associated with CAC among EAs (FDR-corrected p-value < 0.05; Additional file 1: Table S5). Of these, five were inversely associated and seven were positively associated with CAC. Amino acid and lipid metabolism pathways exhibited significant associations with CAC. Interestingly, all four of the metabolites from amino acid metabolism pathways were positively associated with CAC. Of seven metabolites associated with lipid metabolism pathways, five were inversely associated with CAC. Significant subpathways demonstrating an association with CAC included branched-chain amino acid (BCAA), lysine, and phenylalanine metabolism subpathways. When additionally adjusting for LDL-C in the model, 57 metabolites were associated with CAC (Additional file 1: Table S6). The lipid metabolism and amino acid metabolism pathways continued to be the significant pathways associated with CAC in EAs. When the model was further adjusted for statin use, 36 metabolites were significantly associated with CAC (Fig. 2). Interestingly, compared to 7 significant metabolites from the lipid metabolism pathway in the initial model, we found 19 metabolites from the lipid metabolism pathway in the final model adjusted for age, sex, BMI, smoking status, hypertension status, CVD, diabetes duration, date of plasma collection, time between plasma collection and CT exam, LDL-C, and statin use (Additional file 1: Table S7). The additional lipid metabolites were lysoplasmalogen and pregnenolone/progestin steroids, positively and negatively associated with CAC in EAs, respectively. Table 2 demonstrates the number of significant lipid metabolites associated with CAC among EAs in three different models. Results from the mediation analysis (Additional file 1: Table S15) did not suggest that duration of diabetes significantly impacted these findings. In addition, the impact of HbA1c as an alternative to diabetes duration was explored in the fully-adjusted model and the results were not significantly different. (Additional file 1: Table S9).

Between AAs and EAs, there was a modest overlap of nominally associated (P < 0.05) metabolites in AAs (N = 10; Additional file 1: Table S10) or EAs (N = 5; Additional file 1: Table S11) in the final model adjusted for age, sex, BMI, smoking status, hypertension status, CVD, diabetes duration, date of plasma collection, time between plasma collection and CT exam, LDL-C, and statin use. In AAs, seven androgenic steroid metabolites in the lipid metabolism pathway displayed cross-ancestry replication. In EAs, one androgenic steroid, a medium-chain fatty acid, and two pregnenolone steroid metabolites in the lipid metabolism pathway displayed cross-ancestry replication. These results highlight androgenic steroid metabolism among metabolic subpathways significantly associated with CAC in AAs and EAs.

When using a CAC threshold of 10 for presence (≥ 10) versus absence (< 10), there were 49 metabolites associated with coronary atherosclerosis in AA and 2 metabolites associated with coronary atherosclerosis in EAs in a model adjusted for age, sex, BMI, smoking status, hypertension status, CVD, diabetes duration, LDL-C, and statin use (Additional file 1: Table S12–S13). Date of plasma collection and time between plasma collection and CT exam was not included in the model owing to collinearity among the predictors. For AAs, 19 of these 49 metabolites were found to be significantly associated with CAC in our fully adjusted model using linear regression analysis (Additional file 1: Table S4). For EA, both metabolites were found to be significantly associated with CAC in a similar model using linear regression analysis (Additional file 1: Table S7).

As for the known difference in levels of CAC in AAs and EAs, there were clear distinctions in the association of metabolic pathways and CAC between these ancestral populations. For lipid metabolism pathways, beyond androgenic steroid metabolism, the highest number of metabolites significantly associated with CAC in AAs derived from fatty acid and bile acid metabolism subpathways. In contrast, a significant number of metabolites from lysoplasmalogen, pregnenolone steroids, and progestin steroids metabolism subpathways were associated with CAC in EAs.

Finally, to validate the associations observed between metabolites and subclinical atherosclerosis, replication of significant metabolites was assessed in an independent dataset of 673 DHS participants inclusive of 181 AAs and 492 EAs. The baseline characteristics of these participants are shown in Additional file 1: Table S16. For the model adjusted for age, sex, BMI, smoking status, hypertension, CVD, duration of diabetes, date of plasma collection, time between plasma collection and CT exam, LDL-C, statin use, no metabolites replicated in AAs, whereas 7 metabolites replicated in EAs (Additional file 1: Table S17, S18). If we were to increase the p-value threshold of statistical significance to 0.10, it would increase the number of significant metabolites associated with CAC from 7 to 10. Among the pathways represented, the majority were from lipid metabolism.

## Discussion

This study investigated the metabolomic profile of a high-risk population with longstanding T2D and assessed ancestry-specific associations with subclinical atherosclerosis. The number of metabolites associated with CAC were similar in EAs and AAs in a fully adjusted model. In addition, while androgenic steroid metabolism was associated with CAC in both groups, the association of other lipid metabolism subpathways with CAC differed. The number of metabolites from lipid metabolism pathways that were significantly associated with CAC was reduced after adjusting for LDL-C and statin usage for AAs and EAs, highlighting androgenic steroid and fatty acid metabolism.

There are a limited number of studies that have evaluated metabolic pathways involved in subclinical atherosclerosis. In a large analysis with more than 7000 participants from three prospective population-based cohorts, lipid, carbohydrate, BCAA, and aromatic amino acid metabolism, as well as oxidative stress and inflammatory pathways, were significantly associated with CAC and carotid intima-media thickness (cIMT) [13]. A study in 5487 British and Australian individuals assessed subclinical atherosclerosis using cIMT and arterial pulse wave velocity (PWV) [12]. No metabolites were associated with cIMT, while glucose and amino acid metabolism pathways demonstrated modest associations with PWV [12]. Significant alterations in lipid and BCAA metabolism were also associated with PWV and cIMT in a middle-aged Chinese population [14]. Unlike the current report, these studies did not examine the influence of ancestry or study populations at high risk for CVD based on enrichment for T2D.

The present report demonstrates that metabolites linked to the androgenic steroid metabolism subpathway were inversely associated with CAC among AAs and EAs. Many studies have investigated the relationship between testosterone and CVD; however, results have been conflicting [21]. A study of 2416 men demonstrated that high serum testosterone concentrations were inversely associated with the risk of cardiovascular events over 5 years [22]. Another longitudinal study found an association between low serum testosterone and increased mortality in men with coronary heart disease [23]. The Cardiovascular Trial of the Testosterone Trials (TTrials) studied the effects of testosterone therapy on coronary artery plaque volume, assessed by coronary computed tomographic angiography (CCTA) in 138 testosterone-deficient men 65 years and older [24]. The study demonstrated that the treatment with testosterone was associated with a significantly greater increase in coronary artery non-calcified plaque volume compared with placebo at the end of 1 year. However, few studies have examined the association between androgen level and CAC [25, 26]. In male MESA participants, testosterone was inversely associated with the progression of CAC [27]. Interestingly, significant associations were not detected between sex hormones and prevalent CAC among post-menopausal women in MESA [28]. The mechanisms whereby androgens affect atherosclerosis remain unclear. Overall, despite multiple cohort studies demonstrating an association between low testosterone level and increased mortality, it is unknown whether this is a causal relationship or low testosterone level is a marker of poor overall health [29].

We found that metabolites associated with primary and secondary bile acid metabolism subpathways were positively associated with CAC in AAs. Excess cholesterol is converted into bile acids in the liver and excreted from the body [30]. Compared to individuals without coronary artery disease (CAD), patients with CAD reportedly have lower fecal bile acid excretion, and this supports a potential protective effect of bile acid excretion on the development of CAD [31, 32]. A study of 7438 participants who had undergone coronary angiography examined the relationships between fasting serum total bile acids and presence of CAD and MI and the severity of coronary lesions [33]. The study results concluded that the fasting serum total bile acids level was positively associated with the presence and severity of CAD. The conjugated primary bile acids are converted into secondary bile acids (deoxycholic acid and lithocholic acid) after deconjugation and dihydroxylation via intestinal microbiota in the gut [34]. A study of patients with moderate to severe chronic kidney disease demonstrated that higher serum levels of deoxycholic acid were independently associated with higher CAC [35]. We note that metabolites associated with the secondary bile acid metabolism subpathway (glycohyocholate, hyocholate, and taurodeoxycholic acid 3-sulfate) were associated with higher CAC in the present report. The evidence suggests that different components of bile acids perform varying functions and, although the explicit mechanisms remain unclear, bile acids may play an essential role in the pathophysiological process of CAD [33].

The metabolites of BCAA metabolism have been associated with insulin resistance and obesity [36, 37]. A study including 27,041 Women’s Health Study participants examined the association of plasma BCAA metabolites and incident CVD [38]. The study demonstrated a significant positive association between plasma BCAAs with incident CVD. Interestingly, the association was much stronger for the women who developed T2D before the CVD event. The authors concluded that impaired BCAA metabolism might represent a shared pathway of the metabolic pathophysiology that links the risks of T2D and CVD. Metabolites within the BCAA catabolic pathway are also significantly associated with coronary artery disease, independent of their relationship with insulin resistance and diabetes [39, 40]. The present study demonstrated that two BCAA metabolism subpathway metabolites were significantly associated with CAC in EAs, and both were positively associated. A cross-sectional analysis in more than 5000 individuals without CVD demonstrated that a risk score, including BCAA and aromatic amino acids (tyrosine and phenylalanine), was associated with increased cIMT [3]. Another study in 472 Chinese participants demonstrated an association between BCAA and higher cIMT. The explicit mechanisms underlying the association between BCAA and atherosclerosis is unclear. We also found that metabolites of the phenylalanine (aromatic amino acid) metabolism subpathway (N-acetylphenylalanine) were associated with higher CAC. Aromatic amino acids are associated with metabolic risk factors, including insulin resistance and dyslipidemia [41, 42].

Among additional pathways implicated in CVD, we identified a metabolite from the eicosanoid subpathway (leukotriene B4) was associated with higher CAC in EAs and showed replication in the second cohort. Eicosanoids are a group of compounds derived from polyunsaturated fatty acids. Eicosanoids can be pro-inflammatory or anti-inflammatory [43]. Leukotriene B4 (LTB4) is a potent chemoattractant and is known to promote several inflammatory diseases, including atherosclerosis [44]. LTB4 levels were significantly increased in plaque tissue from patients with diabetes undergoing carotid endarterectomy compared to tissue from non-diabetic patients [45]. In addition, a metabolite of diacylglycerol metabolism (linoleoyl-arachidonoyl-glycerol (18:2/20:4)) was associated with lower CAC in EAs, but did not replicate in the second cohort. Diacylglycerol (DAG) functions as a signaling lipid as well as an intermediary in lipid biosynthesis pathways [46]. Studies have demonstrated an association between dietary DAG and plasma lipid concentrations [47–50]. A randomized controlled trial of individuals with T2D demonstrated that ingestion of DAG oil was associated with decreased waist circumference and serum triglyceride concentrations [48]. A recent study, including 658 participants from the Framingham Heart Study (FHS), attempted to identify lipid metabolomic biomarkers associated with metabolic risk factors using plasma lipidomic profiling [51]. Several DAG metabolites were found to be significantly associated with obesity and dyslipidemia. The significant association of DAG metabolites with CAC in our study could be attributed to its effect on lipid metabolism.

We also found metabolites of lysoplasmalogen metabolism pathways were significantly associated with CAC in EAs and demonstrated replication in a second cohort. Plasmalogens are subclasses of glycerophospholipids that are characterized by a cis vinyl ether bond linking an alkyl chain to the sn-1 position of the glycerol backbone and are particularly abundant in neurons, cardiac and skeletal muscle [52]. The physiological functions of plasmalogens are not entirely understood. Plasmalogens have been proposed to be atheroprotective, partly because of their anti-oxidant properties [52]. A study including 3779 patients with T2D demonstrated that plasmalogens containing primarily polyunsaturated fatty acids were inversely associated with future cardiovascular events [16]. It is postulated that plasmalogens are oxidized in situations of heightened oxidative stress, leading to an upregulation of the biosynthetic pathway. Another study including subjects with Glucokinase-maturity onset diabetes of the young (GCK-MODY) and T2D demonstrated a strong positive associations between serum plasmalogen phosphatidylcholines and total HDL in hyperglycemic individuals [53]. The authors concluded that in GCK-MODY, HDL-localized increases in the levels of the proteins adipose triglyceride lipase (ATGL) and choline/ethanolamine phosphotransferase 1 (CEPT1) could lead to higher levels of plasmalogen phosphatidylcholines and provide vascular protection against chronic hyperglycemia. A negative association between circulating plasmalogens and coronary artery disease has been reported previously [54]. In vitro studies have indicated that plasmalogens are capable of reducing the oxidation of cell membrane cholesterol and LDL-C [55]. Thus, plasmalogens may represent a potential therapeutic target for the prevention of atherosclerosis.

An important finding from this study is the notably different metabolite profile association with CAC in AAs and EAs. Multiple studies reveal that AAs have markedly lower levels of CAC than EAs. This observation is consistent in persons with and without T2D [56, 57]. Although not entirely clear, differences have been attributed to calcium metabolism, inflammatory markers, hemostasis, fibrinolysis, or genetic factors related to calcification [58, 59]. A study evaluating participants from MESA, DHS, and FHS demonstrated that genomic regions on chromosomes 1, 2, 4, 8, 9, 11, and 13 identified by admixture mapping appear to contribute to ethnic differences in susceptibility to CAC between AAs and EAs [60]. Our validation cohort also revealed different metabolic pathways associated with CAC among AAs and EAs. To our knowledge, none of the previous studies have examined the racial/ethnic difference in the association of metabolomics profile, and CAC and future studies are warranted to replicate our novel findings.

Given the unique cohort enriched for T2D, it is noteworthy that many of the significant metabolites we found associated with subclinical atherosclerosis have also shown an association with T2D. In a study utilizing a non-targeted approach, metabolites of 3 bile acids were positively associated with incident T2D [61]. A prospective analysis of FHS participants demonstrated a positive association between BCAA metabolites and the development of T2D [62]. Multiple studies have also shown a positive association between metabolites of aromatic amino acids and T2D [63, 64]. Many phospholipids, including sphingomyelin, which showed significant association in our study, have demonstrated an inverse association with T2D in previous studies [63, 65]. Interestingly, a study analyzing 9637 participants from the Mexican Study on Nutritional and Psychosocial Markers of Frailty, aimed to identify clinical and biological predictors of regression to normoglycemia and establish whether adding information from an NMR-based set of plasma metabolites could improve the predictive capability for regression to normoglycemia beyond clinical factors. Results showed that the addition of an NMR-based set of metabolomics information did not improve the capability to predict regression to normoglycemia [66].

This study has several strengths. Our study builds upon previous studies examining the association of expanded profiling of plasma metabolites, beyond lipidomics, with subclinical atherosclerosis represented by CAC in a high-risk cohort enriched for T2D. Data were analyzed in two independent ancestral groups with markedly different propensities for developing calcified atherosclerotic plaque. We detected novel metabolite associations with subclinical atherosclerosis and characterized the underlying pathways associated with metabolites of interest. Limitations included the cross-sectional nature of the association between plasma metabolites and CAC; thus, causal relationships could not be established. In addition, we focused on circulating metabolites and applied an acquisition method that was not ‘completely’ untargeted, as it was restricted to a library of known metabolites. Finally, the overall modest sample size and ethnic-specific sample size differences may have negatively impacted our ability to observe a more modest effect and assess cross-ancestry replication, respectively.

## Conclusion

In conclusion, the metabolic pathways associated with subclinical atherosclerosis differ between AA and EA populations with T2D. The number of metabolites significantly associated with CAC appeared to be higher in EAs compared with AAs. European-derived populations have far higher levels of CAC than AAs and are at increased risk for myocardial infarction when healthcare access is equivalent between populations [67, 68]. Androgenic steroid, fatty acid, and bile acid metabolism subpathways were significantly associated with CAC in AAs, while androgenic steroid, progestin steroid, pregnenolone steroid, lysoplasmalogen, sphingomyelins, and BCAA metabolism subpathways were associated with CAC in EAs. Further studies are required to understand the mechanistic significance of these metabolites in cardiovascular disease.

## Supplementary Information

**Additional file 1.** Additional tables.

## Data Availability

The datasets used and/or analyzed during the current study are available from the corresponding author on reasonable request.

## Abbreviations

AA: African American
BCAA: Branched-chain amino acid
CAC: Coronary artery calcium
CAD: Coronary artery disease
CARDIA: Coronary Artery Risk Development in Young Adults
CCTA: Coronary computed tomographic angiography
cIMT: Carotid intima-media thickness
CVD: Cardiovascular disease
DAG: Diacylglycerol
DHS: Diabetes Heart Study
EA: European American
GEE: Generalized estimating equations
HDL-C: High-density lipoprotein cholesterol
LDL-C: Low-density lipoprotein cholesterol
MESA: Multi-Ethnic Study of Atherosclerosis
NHLBI: National Heart, Lung, and Blood Institute
PWV: Pulse wave velocity
T2D: Type 2 diabetes
