# Supplementary material for: Plasma metabolomic profiling in subclinical atherosclerosis: the Diabetes Heart Study
Source: Cardiovasc Diabetol. 2021 Dec 7;20:231. doi: 10.1186/s12933-021-01419-y (PMC8653597; doi:10.1186/s12933-021-01419-y)
Supplement: Supplementary file 1 — Additional file 1. Additional tables. [file 12933_2021_1419_MOESM1_ESM.docx]

**Additional Material**

**Plasma Metabolomic Profiling in Subclinical Atherosclerosis: the Diabetes Heart Study**

Parag Anilkumar Chevli MBBS, MS^1^, Barry I. Freedman MD^2^, Fang-Chi Hsu PhD^3^, Jianzhao Xu BS^4^, Megan E. Rudock, PhD^4^, Lijun Ma, MD, PhD^2^, John S. Parks PhD^5^, Nicholette D. Palmer PhD^4^, Michael D. Shapiro DO, MCR^6^

1. Section on Hospital Medicine, Department of Internal Medicine, Wake Forest School of Medicine, Winston-Salem, NC
2. Section on Nephrology, Department of Internal Medicine, Wake Forest School of Medicine, Winston-Salem, NC
3. Department of Biostatistics and Data Science, Division of Public Health Sciences, Wake Forest School of Medicine, Winston-Salem, NC
4. Department of Biochemistry, Wake Forest School of Medicine, Winston-Salem, NC
5. Section on Molecular Medicine, Department of Internal Medicine, Wake Forest School of Medicine, Winston-Salem, NC
6. Section of Cardiovascular Medicine, Center for Preventive Cardiology, Wake Forest School of Medicine, Winston-Salem, NC

**Additional file 1: Table SⅠ. List of Known Metabolites Evaluated in the Diabetes Heart Study.**

| **Biochemical** | **Super-pathway** | **Sub-pathway** | **HMDB** |
| --- | --- | --- | --- |
| alanine | Amino Acid | Alanine and Aspartate Metabolism | [HMDB00161](http://www.hmdb.ca/metabolites/HMDB00161) |
| asparagine | Amino Acid | Alanine and Aspartate Metabolism | [HMDB00168](http://www.hmdb.ca/metabolites/HMDB00168) |
| aspartate | Amino Acid | Alanine and Aspartate Metabolism | [HMDB00191](http://www.hmdb.ca/metabolites/HMDB00191) |
| hydroxyasparagine | Amino Acid | Alanine and Aspartate Metabolism | [HMDB32332](http://www.hmdb.ca/metabolites/HMDB32332) |
| N,N-dimethylalanine | Amino Acid | Alanine and Aspartate Metabolism |  |
| N-acetylalanine | Amino Acid | Alanine and Aspartate Metabolism | [HMDB00766](http://www.hmdb.ca/metabolites/HMDB00766) |
| N-acetylasparagine | Amino Acid | Alanine and Aspartate Metabolism | [HMDB06028](http://www.hmdb.ca/metabolites/HMDB06028) |
| N-acetylaspartate (NAA) | Amino Acid | Alanine and Aspartate Metabolism | [HMDB00812](http://www.hmdb.ca/metabolites/HMDB00812) |
| N-carbamoylalanine | Amino Acid | Alanine and Aspartate Metabolism |  |
| creatine | Amino Acid | Creatine Metabolism | [HMDB00064](http://www.hmdb.ca/metabolites/HMDB00064) |
| creatinine | Amino Acid | Creatine Metabolism | [HMDB00562](http://www.hmdb.ca/metabolites/HMDB00562) |
| guanidinoacetate | Amino Acid | Creatine Metabolism | [HMDB00128](http://www.hmdb.ca/metabolites/HMDB00128) |
| 4-hydroxyglutamate | Amino Acid | Glutamate Metabolism | [HMDB01344](http://www.hmdb.ca/metabolites/HMDB01344) |
| alpha-ketoglutaramate* | Amino Acid | Glutamate Metabolism |  |
| beta-citrylglutamate | Amino Acid | Glutamate Metabolism |  |
| carboxyethyl-GABA | Amino Acid | Glutamate Metabolism | [HMDB02201](http://www.hmdb.ca/metabolites/HMDB02201) |
| citramalate | Amino Acid | Glutamate Metabolism | [HMDB00426](http://www.hmdb.ca/metabolites/HMDB00426) |
| gamma-carboxyglutamate | Amino Acid | Glutamate Metabolism | [HMDB41900](http://www.hmdb.ca/metabolites/HMDB41900) |
| glutamate | Amino Acid | Glutamate Metabolism | [HMDB00148](http://www.hmdb.ca/metabolites/HMDB00148) |
| glutamate, gamma-methyl ester | Amino Acid | Glutamate Metabolism | [HMDB61715](http://www.hmdb.ca/metabolites/HMDB61715) |
| glutamine | Amino Acid | Glutamate Metabolism | [HMDB00641](http://www.hmdb.ca/metabolites/HMDB00641) |
| N-acetyl-aspartyl-glutamate (NAAG) | Amino Acid | Glutamate Metabolism | [HMDB01067](http://www.hmdb.ca/metabolites/HMDB01067) |
| N-acetylglutamate | Amino Acid | Glutamate Metabolism | [HMDB01138](http://www.hmdb.ca/metabolites/HMDB01138) |
| N-acetylglutamine | Amino Acid | Glutamate Metabolism | [HMDB06029](http://www.hmdb.ca/metabolites/HMDB06029) |
| pyroglutamine* | Amino Acid | Glutamate Metabolism |  |
| S-1-pyrroline-5-carboxylate | Amino Acid | Glutamate Metabolism | [HMDB01301](http://www.hmdb.ca/metabolites/HMDB01301) |
| 2-aminobutyrate | Amino Acid | Glutathione Metabolism | [HMDB00650](http://www.hmdb.ca/metabolites/HMDB00650) |
| 2-hydroxybutyrate/2-hydroxyisobutyrate | Amino Acid | Glutathione Metabolism |  |
| 5-oxoproline | Amino Acid | Glutathione Metabolism | [HMDB00267](http://www.hmdb.ca/metabolites/HMDB00267) |
| cys-gly, oxidized | Amino Acid | Glutathione Metabolism |  |
| cysteine-glutathione disulfide | Amino Acid | Glutathione Metabolism | [HMDB00656](http://www.hmdb.ca/metabolites/HMDB00656) |
| cysteinylglycine | Amino Acid | Glutathione Metabolism | [HMDB00078](http://www.hmdb.ca/metabolites/HMDB00078) |
| cysteinylglycine disulfide* | Amino Acid | Glutathione Metabolism | [HMDB00709](http://www.hmdb.ca/metabolites/HMDB00709) |
| 2-methylserine | Amino Acid | Glycine, Serine and Threonine Metabolism |  |
| betaine | Amino Acid | Glycine, Serine and Threonine Metabolism | [HMDB00043](http://www.hmdb.ca/metabolites/HMDB00043) |
| dimethylglycine | Amino Acid | Glycine, Serine and Threonine Metabolism | [HMDB00092](http://www.hmdb.ca/metabolites/HMDB00092) |
| glycine | Amino Acid | Glycine, Serine and Threonine Metabolism | [HMDB00123](http://www.hmdb.ca/metabolites/HMDB00123) |
| N-acetylglycine | Amino Acid | Glycine, Serine and Threonine Metabolism | [HMDB00532](http://www.hmdb.ca/metabolites/HMDB00532) |
| N-acetylserine | Amino Acid | Glycine, Serine and Threonine Metabolism | [HMDB02931](http://www.hmdb.ca/metabolites/HMDB02931) |
| N-acetylthreonine | Amino Acid | Glycine, Serine and Threonine Metabolism | [HMDB62557](http://www.hmdb.ca/metabolites/HMDB62557) |
| sarcosine | Amino Acid | Glycine, Serine and Threonine Metabolism | [HMDB00271](http://www.hmdb.ca/metabolites/HMDB00271) |
| serine | Amino Acid | Glycine, Serine and Threonine Metabolism | [HMDB00187](http://www.hmdb.ca/metabolites/HMDB00187) |
| threonine | Amino Acid | Glycine, Serine and Threonine Metabolism | [HMDB00167](http://www.hmdb.ca/metabolites/HMDB00167) |
| 1-methylguanidine | Amino Acid | Guanidino and Acetamido Metabolism | [HMDB01522](http://www.hmdb.ca/metabolites/HMDB01522) |
| 4-guanidinobutanoate | Amino Acid | Guanidino and Acetamido Metabolism | [HMDB03464](http://www.hmdb.ca/metabolites/HMDB03464) |
| 1-methyl-4-imidazoleacetate | Amino Acid | Histidine Metabolism | [HMDB02820](http://www.hmdb.ca/metabolites/HMDB02820) |
| 1-methyl-5-imidazoleacetate | Amino Acid | Histidine Metabolism | [HMDB04988](http://www.hmdb.ca/metabolites/HMDB04988) |
| 1-methylhistidine | Amino Acid | Histidine Metabolism | [HMDB00001](http://www.hmdb.ca/metabolites/HMDB00001) |
| 1-ribosyl-imidazoleacetate* | Amino Acid | Histidine Metabolism | [HMDB02331](http://www.hmdb.ca/metabolites/HMDB02331) |
| 3-methylhistidine | Amino Acid | Histidine Metabolism | [HMDB00479](http://www.hmdb.ca/metabolites/HMDB00479) |
| carnosine | Amino Acid | Histidine Metabolism | [HMDB00033](http://www.hmdb.ca/metabolites/HMDB00033) |
| cis-urocanate | Amino Acid | Histidine Metabolism | [HMDB34174](http://www.hmdb.ca/metabolites/HMDB34174) |
| formiminoglutamate | Amino Acid | Histidine Metabolism | [HMDB00854](http://www.hmdb.ca/metabolites/HMDB00854) |
| histidine | Amino Acid | Histidine Metabolism | [HMDB00177](http://www.hmdb.ca/metabolites/HMDB00177) |
| hydantoin-5-propionate | Amino Acid | Histidine Metabolism | [HMDB01212](http://www.hmdb.ca/metabolites/HMDB01212) |
| imidazole lactate | Amino Acid | Histidine Metabolism | [HMDB02320](http://www.hmdb.ca/metabolites/HMDB02320) |
| imidazole propionate | Amino Acid | Histidine Metabolism | [HMDB02271](http://www.hmdb.ca/metabolites/HMDB02271) |
| N-acetyl-1-methylhistidine* | Amino Acid | Histidine Metabolism |  |
| N-acetyl-3-methylhistidine* | Amino Acid | Histidine Metabolism |  |
| N-acetylcarnosine | Amino Acid | Histidine Metabolism | [HMDB12881](http://www.hmdb.ca/metabolites/HMDB12881) |
| N-acetylhistamine | Amino Acid | Histidine Metabolism | [HMDB13253](http://www.hmdb.ca/metabolites/HMDB13253) |
| N-acetylhistidine | Amino Acid | Histidine Metabolism | [HMDB32055](http://www.hmdb.ca/metabolites/HMDB32055) |
| trans-urocanate | Amino Acid | Histidine Metabolism | [HMDB00301](http://www.hmdb.ca/metabolites/HMDB00301) |
| 1-carboxyethylisoleucine | Amino Acid | Leucine, Isoleucine and Valine Metabolism |  |
| 1-carboxyethylleucine | Amino Acid | Leucine, Isoleucine and Valine Metabolism |  |
| 1-carboxyethylvaline | Amino Acid | Leucine, Isoleucine and Valine Metabolism |  |
| 2,3-dihydroxy-2-methylbutyrate | Amino Acid | Leucine, Isoleucine and Valine Metabolism | [HMDB29576](http://www.hmdb.ca/metabolites/HMDB29576) |
| 2-hydroxy-3-methylvalerate | Amino Acid | Leucine, Isoleucine and Valine Metabolism | [HMDB00317](http://www.hmdb.ca/metabolites/HMDB00317) |
| 2-ketocaprylate | Amino Acid | Leucine, Isoleucine and Valine Metabolism | [HMDB13211](http://www.hmdb.ca/metabolites/HMDB13211) |
| 2-methylbutyrylcarnitine (C5) | Amino Acid | Leucine, Isoleucine and Valine Metabolism | [HMDB00378](http://www.hmdb.ca/metabolites/HMDB00378) |
| 2-methylbutyrylglycine (C5) | Amino Acid | Leucine, Isoleucine and Valine Metabolism | [HMDB00339](http://www.hmdb.ca/metabolites/HMDB00339) |
| 3-hydroxy-2-ethylpropionate | Amino Acid | Leucine, Isoleucine and Valine Metabolism | [HMDB00396](http://www.hmdb.ca/metabolites/HMDB00396) |
| 3-hydroxyisobutyrate | Amino Acid | Leucine, Isoleucine and Valine Metabolism | [HMDB00336](http://www.hmdb.ca/metabolites/HMDB00336) |
| 3-methyl-2-oxobutyrate | Amino Acid | Leucine, Isoleucine and Valine Metabolism | [HMDB00019](http://www.hmdb.ca/metabolites/HMDB00019) |
| 3-methyl-2-oxovalerate | Amino Acid | Leucine, Isoleucine and Valine Metabolism | [HMDB03736](http://www.hmdb.ca/metabolites/HMDB03736) |
| 3-methylcrotonylglycine | Amino Acid | Leucine, Isoleucine and Valine Metabolism | [HMDB00459](http://www.hmdb.ca/metabolites/HMDB00459) |
| 3-methylglutaconate | Amino Acid | Leucine, Isoleucine and Valine Metabolism | [HMDB00522](http://www.hmdb.ca/metabolites/HMDB00522) |
| 3-methylglutarylcarnitine (2) | Amino Acid | Leucine, Isoleucine and Valine Metabolism | [HMDB00552](http://www.hmdb.ca/metabolites/HMDB00552) |
| 4-methyl-2-oxopentanoate | Amino Acid | Leucine, Isoleucine and Valine Metabolism | [HMDB00695](http://www.hmdb.ca/metabolites/HMDB00695) |
| alpha-hydroxyisocaproate | Amino Acid | Leucine, Isoleucine and Valine Metabolism | [HMDB00746](http://www.hmdb.ca/metabolites/HMDB00746) |
| alpha-hydroxyisovalerate | Amino Acid | Leucine, Isoleucine and Valine Metabolism | [HMDB00407](http://www.hmdb.ca/metabolites/HMDB00407) |
| beta-hydroxyisovalerate | Amino Acid | Leucine, Isoleucine and Valine Metabolism | [HMDB00754](http://www.hmdb.ca/metabolites/HMDB00754) |
| beta-hydroxyisovaleroylcarnitine | Amino Acid | Leucine, Isoleucine and Valine Metabolism |  |
| ethylmalonate | Amino Acid | Leucine, Isoleucine and Valine Metabolism | [HMDB00622](http://www.hmdb.ca/metabolites/HMDB00622) |
| isobutyrylcarnitine (C4) | Amino Acid | Leucine, Isoleucine and Valine Metabolism | [HMDB00736](http://www.hmdb.ca/metabolites/HMDB00736) |
| isobutyrylglycine (C4) | Amino Acid | Leucine, Isoleucine and Valine Metabolism | [HMDB00730](http://www.hmdb.ca/metabolites/HMDB00730) |
| isoleucine | Amino Acid | Leucine, Isoleucine and Valine Metabolism | [HMDB00172](http://www.hmdb.ca/metabolites/HMDB00172) |
| isovalerate (C5) | Amino Acid | Leucine, Isoleucine and Valine Metabolism | [HMDB00718](http://www.hmdb.ca/metabolites/HMDB00718) |
| isovalerylcarnitine (C5) | Amino Acid | Leucine, Isoleucine and Valine Metabolism | [HMDB00688](http://www.hmdb.ca/metabolites/HMDB00688) |
| isovalerylglycine | Amino Acid | Leucine, Isoleucine and Valine Metabolism | [HMDB00678](http://www.hmdb.ca/metabolites/HMDB00678) |
| leucine | Amino Acid | Leucine, Isoleucine and Valine Metabolism |  |
| methylsuccinate | Amino Acid | Leucine, Isoleucine and Valine Metabolism | [HMDB01844](http://www.hmdb.ca/metabolites/HMDB01844) |
| methylsuccinoylcarnitine | Amino Acid | Leucine, Isoleucine and Valine Metabolism |  |
| N-acetylisoleucine | Amino Acid | Leucine, Isoleucine and Valine Metabolism | [HMDB61684](http://www.hmdb.ca/metabolites/HMDB61684) |
| N-acetylleucine | Amino Acid | Leucine, Isoleucine and Valine Metabolism | [HMDB11756](http://www.hmdb.ca/metabolites/HMDB11756) |
| N-acetylvaline | Amino Acid | Leucine, Isoleucine and Valine Metabolism | [HMDB11757](http://www.hmdb.ca/metabolites/HMDB11757) |
| tigloylglycine | Amino Acid | Leucine, Isoleucine and Valine Metabolism | [HMDB00959](http://www.hmdb.ca/metabolites/HMDB00959) |
| tiglyl carnitine (C5) | Amino Acid | Leucine, Isoleucine and Valine Metabolism | [HMDB02366](http://www.hmdb.ca/metabolites/HMDB02366) |
| valine | Amino Acid | Leucine, Isoleucine and Valine Metabolism | [HMDB00883](http://www.hmdb.ca/metabolites/HMDB00883) |
| 2-aminoadipate | Amino Acid | Lysine Metabolism | [HMDB00510](http://www.hmdb.ca/metabolites/HMDB00510) |
| 5-(galactosylhydroxy)-L-lysine | Amino Acid | Lysine Metabolism |  |
| 5-hydroxylysine | Amino Acid | Lysine Metabolism | [HMDB00450](http://www.hmdb.ca/metabolites/HMDB00450) |
| 6-oxopiperidine-2-carboxylate | Amino Acid | Lysine Metabolism | [HMDB61705](http://www.hmdb.ca/metabolites/HMDB61705) |
| fructosyllysine | Amino Acid | Lysine Metabolism |  |
| glutarylcarnitine (C5-DC) | Amino Acid | Lysine Metabolism | [HMDB13130](http://www.hmdb.ca/metabolites/HMDB13130) |
| hydroxy-N6,N6,N6-trimethyllysine* | Amino Acid | Lysine Metabolism |  |
| lysine | Amino Acid | Lysine Metabolism | [HMDB00182](http://www.hmdb.ca/metabolites/HMDB00182) |
| N,N,N-trimethyl-5-aminovalerate | Amino Acid | Lysine Metabolism |  |
| N2-acetyl,N6,N6-dimethyllysine | Amino Acid | Lysine Metabolism |  |
| N2-acetyl,N6-methyllysine | Amino Acid | Lysine Metabolism |  |
| N2-acetyllysine | Amino Acid | Lysine Metabolism | [HMDB00446](http://www.hmdb.ca/metabolites/HMDB00446) |
| N6,N6,N6-trimethyllysine | Amino Acid | Lysine Metabolism | [HMDB01325](http://www.hmdb.ca/metabolites/HMDB01325) |
| N6,N6-dimethyllysine | Amino Acid | Lysine Metabolism | [HMDB13287](http://www.hmdb.ca/metabolites/HMDB13287) |
| N6-acetyllysine | Amino Acid | Lysine Metabolism | [HMDB00206](http://www.hmdb.ca/metabolites/HMDB00206) |
| N6-methyllysine | Amino Acid | Lysine Metabolism | [HMDB02038](http://www.hmdb.ca/metabolites/HMDB02038) |
| N-acetyl-cadaverine | Amino Acid | Lysine Metabolism | [HMDB02284](http://www.hmdb.ca/metabolites/HMDB02284) |
| pipecolate | Amino Acid | Lysine Metabolism | [HMDB00070](http://www.hmdb.ca/metabolites/HMDB00070) |
| 2,3-dihydroxy-5-methylthio-4-pentenoate (DMTPA)* | Amino Acid | Methionine, Cysteine, SAM and Taurine Metabolism |  |
| 2-hydroxy-4-(methylthio)butanoic acid | Amino Acid | Methionine, Cysteine, SAM and Taurine Metabolism |  |
| 5-methylthioribose | Amino Acid | Methionine, Cysteine, SAM and Taurine Metabolism | [HMDB01087](http://www.hmdb.ca/metabolites/HMDB01087) |
| alpha-ketobutyrate | Amino Acid | Methionine, Cysteine, SAM and Taurine Metabolism | [HMDB00005](http://www.hmdb.ca/metabolites/HMDB00005) |
| cystathionine | Amino Acid | Methionine, Cysteine, SAM and Taurine Metabolism | [HMDB00099](http://www.hmdb.ca/metabolites/HMDB00099) |
| cysteine | Amino Acid | Methionine, Cysteine, SAM and Taurine Metabolism | [HMDB00574](http://www.hmdb.ca/metabolites/HMDB00574) |
| cysteine s-sulfate | Amino Acid | Methionine, Cysteine, SAM and Taurine Metabolism | [HMDB00731](http://www.hmdb.ca/metabolites/HMDB00731) |
| cysteine sulfinic acid | Amino Acid | Methionine, Cysteine, SAM and Taurine Metabolism | [HMDB00996](http://www.hmdb.ca/metabolites/HMDB00996) |
| cystine | Amino Acid | Methionine, Cysteine, SAM and Taurine Metabolism | [HMDB00192](http://www.hmdb.ca/metabolites/HMDB00192) |
| hypotaurine | Amino Acid | Methionine, Cysteine, SAM and Taurine Metabolism | [HMDB00965](http://www.hmdb.ca/metabolites/HMDB00965) |
| lanthionine | Amino Acid | Methionine, Cysteine, SAM and Taurine Metabolism |  |
| methionine | Amino Acid | Methionine, Cysteine, SAM and Taurine Metabolism | [HMDB00696](http://www.hmdb.ca/metabolites/HMDB00696) |
| methionine sulfone | Amino Acid | Methionine, Cysteine, SAM and Taurine Metabolism |  |
| methionine sulfoxide | Amino Acid | Methionine, Cysteine, SAM and Taurine Metabolism | [HMDB02005](http://www.hmdb.ca/metabolites/HMDB02005) |
| N-acetylmethionine | Amino Acid | Methionine, Cysteine, SAM and Taurine Metabolism | [HMDB11745](http://www.hmdb.ca/metabolites/HMDB11745) |
| N-acetyltaurine | Amino Acid | Methionine, Cysteine, SAM and Taurine Metabolism |  |
| N-formylmethionine | Amino Acid | Methionine, Cysteine, SAM and Taurine Metabolism | [HMDB01015](http://www.hmdb.ca/metabolites/HMDB01015) |
| S-adenosylhomocysteine (SAH) | Amino Acid | Methionine, Cysteine, SAM and Taurine Metabolism | [HMDB00939](http://www.hmdb.ca/metabolites/HMDB00939) |
| S-methylcysteine | Amino Acid | Methionine, Cysteine, SAM and Taurine Metabolism | [HMDB02108](http://www.hmdb.ca/metabolites/HMDB02108) |
| S-methylcysteine sulfoxide | Amino Acid | Methionine, Cysteine, SAM and Taurine Metabolism | [HMDB29432](http://www.hmdb.ca/metabolites/HMDB29432) |
| succinoyltaurine | Amino Acid | Methionine, Cysteine, SAM and Taurine Metabolism |  |
| taurine | Amino Acid | Methionine, Cysteine, SAM and Taurine Metabolism | [HMDB00251](http://www.hmdb.ca/metabolites/HMDB00251) |
| 1-carboxyethylphenylalanine | Amino Acid | Phenylalanine Metabolism |  |
| 2-hydroxyphenylacetate | Amino Acid | Phenylalanine Metabolism | [HMDB00669](http://www.hmdb.ca/metabolites/HMDB00669) |
| 4-hydroxyphenylacetate | Amino Acid | Phenylalanine Metabolism | [HMDB00020](http://www.hmdb.ca/metabolites/HMDB00020) |
| N-acetylphenylalanine | Amino Acid | Phenylalanine Metabolism | [HMDB00512](http://www.hmdb.ca/metabolites/HMDB00512) |
| phenylacetate | Amino Acid | Phenylalanine Metabolism | [HMDB00209](http://www.hmdb.ca/metabolites/HMDB00209) |
| phenylalanine | Amino Acid | Phenylalanine Metabolism | [HMDB00159](http://www.hmdb.ca/metabolites/HMDB00159) |
| phenyllactate (PLA) | Amino Acid | Phenylalanine Metabolism | [HMDB00779](http://www.hmdb.ca/metabolites/HMDB00779) |
| phenylpyruvate | Amino Acid | Phenylalanine Metabolism | [HMDB00205](http://www.hmdb.ca/metabolites/HMDB00205) |
| 4-acetamidobutanoate | Amino Acid | Polyamine Metabolism | [HMDB03681](http://www.hmdb.ca/metabolites/HMDB03681) |
| 5-methylthioadenosine (MTA) | Amino Acid | Polyamine Metabolism | [HMDB01173](http://www.hmdb.ca/metabolites/HMDB01173) |
| acisoga | Amino Acid | Polyamine Metabolism |  |
| N('1)-acetylspermidine | Amino Acid | Polyamine Metabolism | [HMDB01276](http://www.hmdb.ca/metabolites/HMDB01276) |
| N-acetyl-isoputreanine | Amino Acid | Polyamine Metabolism |  |
| N-acetylputrescine | Amino Acid | Polyamine Metabolism | [HMDB02064](http://www.hmdb.ca/metabolites/HMDB02064) |
| spermidine | Amino Acid | Polyamine Metabolism | [HMDB01257](http://www.hmdb.ca/metabolites/HMDB01257) |
| 3-indoxyl sulfate | Amino Acid | Tryptophan Metabolism | [HMDB00682](http://www.hmdb.ca/metabolites/HMDB00682) |
| 5-hydroxyindole sulfate | Amino Acid | Tryptophan Metabolism |  |
| 6-bromotryptophan | Amino Acid | Tryptophan Metabolism |  |
| 8-methoxykynurenate | Amino Acid | Tryptophan Metabolism | [HMDB60426](http://www.hmdb.ca/metabolites/HMDB60426) |
| anthranilate | Amino Acid | Tryptophan Metabolism | [HMDB01123](http://www.hmdb.ca/metabolites/HMDB01123) |
| C-glycosyltryptophan | Amino Acid | Tryptophan Metabolism |  |
| indole-3-carboxylate | Amino Acid | Tryptophan Metabolism | [HMDB03320](http://www.hmdb.ca/metabolites/HMDB03320) |
| indoleacetate | Amino Acid | Tryptophan Metabolism | [HMDB00197](http://www.hmdb.ca/metabolites/HMDB00197) |
| indoleacetoylcarnitine* | Amino Acid | Tryptophan Metabolism |  |
| indoleacetylglutamine | Amino Acid | Tryptophan Metabolism | [HMDB13240](http://www.hmdb.ca/metabolites/HMDB13240) |
| indolelactate | Amino Acid | Tryptophan Metabolism | [HMDB00671](http://www.hmdb.ca/metabolites/HMDB00671) |
| indolepropionate | Amino Acid | Tryptophan Metabolism | [HMDB02302](http://www.hmdb.ca/metabolites/HMDB02302) |
| indoxyl glucuronide | Amino Acid | Tryptophan Metabolism | [HMDB59804](http://www.hmdb.ca/metabolites/HMDB59804) |
| kynurenate | Amino Acid | Tryptophan Metabolism | [HMDB00715](http://www.hmdb.ca/metabolites/HMDB00715) |
| kynurenine | Amino Acid | Tryptophan Metabolism | [HMDB00684](http://www.hmdb.ca/metabolites/HMDB00684) |
| N-acetylkynurenine (2) | Amino Acid | Tryptophan Metabolism |  |
| N-acetyltryptophan | Amino Acid | Tryptophan Metabolism | [HMDB13713](http://www.hmdb.ca/metabolites/HMDB13713) |
| N-formylanthranilic acid | Amino Acid | Tryptophan Metabolism | [HMDB04089](http://www.hmdb.ca/metabolites/HMDB04089) |
| picolinate | Amino Acid | Tryptophan Metabolism | [HMDB02243](http://www.hmdb.ca/metabolites/HMDB02243) |
| serotonin | Amino Acid | Tryptophan Metabolism | [HMDB00259](http://www.hmdb.ca/metabolites/HMDB00259) |
| tryptophan | Amino Acid | Tryptophan Metabolism | [HMDB00929](http://www.hmdb.ca/metabolites/HMDB00929) |
| tryptophan betaine | Amino Acid | Tryptophan Metabolism | [HMDB61115](http://www.hmdb.ca/metabolites/HMDB61115) |
| xanthurenate | Amino Acid | Tryptophan Metabolism | [HMDB00881](http://www.hmdb.ca/metabolites/HMDB00881) |
| 1-carboxyethyltyrosine | Amino Acid | Tyrosine Metabolism |  |
| 3-(4-hydroxyphenyl)lactate (HPLA) | Amino Acid | Tyrosine Metabolism | [HMDB00755](http://www.hmdb.ca/metabolites/HMDB00755) |
| 3-methoxytyrosine | Amino Acid | Tyrosine Metabolism | [HMDB01434](http://www.hmdb.ca/metabolites/HMDB01434) |
| 4-methoxyphenol sulfate | Amino Acid | Tyrosine Metabolism |  |
| 5-hydroxymethyl-2-furoic acid | Amino Acid | Tyrosine Metabolism | [HMDB02432](http://www.hmdb.ca/metabolites/HMDB02432) |
| dopamine 3-O-sulfate | Amino Acid | Tyrosine Metabolism | [HMDB06275](http://www.hmdb.ca/metabolites/HMDB06275) |
| gentisate | Amino Acid | Tyrosine Metabolism | [HMDB00152](http://www.hmdb.ca/metabolites/HMDB00152) |
| homovanillate (HVA) | Amino Acid | Tyrosine Metabolism | [HMDB00118](http://www.hmdb.ca/metabolites/HMDB00118) |
| N-acetyltyrosine | Amino Acid | Tyrosine Metabolism | [HMDB00866](http://www.hmdb.ca/metabolites/HMDB00866) |
| N-formylphenylalanine | Amino Acid | Tyrosine Metabolism |  |
| p-cresol glucuronide* | Amino Acid | Tyrosine Metabolism | [HMDB11686](http://www.hmdb.ca/metabolites/HMDB11686) |
| phenol glucuronide | Amino Acid | Tyrosine Metabolism | [HMDB60014](http://www.hmdb.ca/metabolites/HMDB60014) |
| phenol sulfate | Amino Acid | Tyrosine Metabolism | [HMDB60015](http://www.hmdb.ca/metabolites/HMDB60015) |
| thyroxine | Amino Acid | Tyrosine Metabolism | [HMDB01918](http://www.hmdb.ca/metabolites/HMDB01918) |
| tyramine O-sulfate | Amino Acid | Tyrosine Metabolism | [HMDB06409](http://www.hmdb.ca/metabolites/HMDB06409) |
| tyrosine | Amino Acid | Tyrosine Metabolism | [HMDB00158](http://www.hmdb.ca/metabolites/HMDB00158) |
| vanillactate | Amino Acid | Tyrosine Metabolism | [HMDB00913](http://www.hmdb.ca/metabolites/HMDB00913) |
| vanillylmandelate (VMA) | Amino Acid | Tyrosine Metabolism | [HMDB00291](http://www.hmdb.ca/metabolites/HMDB00291) |
| 2-oxoarginine* | Amino Acid | Urea cycle; Arginine and Proline Metabolism | [HMDB04225](http://www.hmdb.ca/metabolites/HMDB04225) |
| 3-amino-2-piperidone | Amino Acid | Urea cycle; Arginine and Proline Metabolism | [HMDB00323](http://www.hmdb.ca/metabolites/HMDB00323) |
| argininate* | Amino Acid | Urea cycle; Arginine and Proline Metabolism | [HMDB03148](http://www.hmdb.ca/metabolites/HMDB03148) |
| arginine | Amino Acid | Urea cycle; Arginine and Proline Metabolism | [HMDB00517](http://www.hmdb.ca/metabolites/HMDB00517) |
| citrulline | Amino Acid | Urea cycle; Arginine and Proline Metabolism | [HMDB00904](http://www.hmdb.ca/metabolites/HMDB00904) |
| dimethylarginine (ADMA + SDMA) | Amino Acid | Urea cycle; Arginine and Proline Metabolism | [HMDB01539](http://www.hmdb.ca/metabolites/HMDB01539) |
| homoarginine | Amino Acid | Urea cycle; Arginine and Proline Metabolism | [HMDB00670](http://www.hmdb.ca/metabolites/HMDB00670) |
| homocitrulline | Amino Acid | Urea cycle; Arginine and Proline Metabolism | [HMDB00679](http://www.hmdb.ca/metabolites/HMDB00679) |
| hydroxyproline | Amino Acid | Urea cycle; Arginine and Proline Metabolism | [HMDB00725](http://www.hmdb.ca/metabolites/HMDB00725) |
| methylurea | Amino Acid | Urea cycle; Arginine and Proline Metabolism |  |
| N,N,N-trimethyl-alanylproline betaine (TMAP) | Amino Acid | Urea cycle; Arginine and Proline Metabolism |  |
| N2,N5-diacetylornithine | Amino Acid | Urea cycle; Arginine and Proline Metabolism |  |
| N-acetylarginine | Amino Acid | Urea cycle; Arginine and Proline Metabolism | [HMDB04620](http://www.hmdb.ca/metabolites/HMDB04620) |
| N-acetylcitrulline | Amino Acid | Urea cycle; Arginine and Proline Metabolism | [HMDB00856](http://www.hmdb.ca/metabolites/HMDB00856) |
| N-alpha-acetylornithine | Amino Acid | Urea cycle; Arginine and Proline Metabolism | [HMDB03357](http://www.hmdb.ca/metabolites/HMDB03357) |
| N-delta-acetylornithine | Amino Acid | Urea cycle; Arginine and Proline Metabolism |  |
| N-methylhydroxyproline | Amino Acid | Urea cycle; Arginine and Proline Metabolism |  |
| N-methylproline | Amino Acid | Urea cycle; Arginine and Proline Metabolism |  |
| ornithine | Amino Acid | Urea cycle; Arginine and Proline Metabolism | [HMDB03374](http://www.hmdb.ca/metabolites/HMDB03374) |
| proline | Amino Acid | Urea cycle; Arginine and Proline Metabolism | [HMDB00162](http://www.hmdb.ca/metabolites/HMDB00162) |
| prolylhydroxyproline | Amino Acid | Urea cycle; Arginine and Proline Metabolism | [HMDB06695](http://www.hmdb.ca/metabolites/HMDB06695) |
| urea | Amino Acid | Urea cycle; Arginine and Proline Metabolism | [HMDB00294](http://www.hmdb.ca/metabolites/HMDB00294) |
| N6-carboxymethyllysine | Carbohydrate | Advanced Glycation End-product |  |
| erythronate* | Carbohydrate | Aminosugar Metabolism | [HMDB00613](http://www.hmdb.ca/metabolites/HMDB00613) |
| glucuronate | Carbohydrate | Aminosugar Metabolism | [HMDB00127](http://www.hmdb.ca/metabolites/HMDB00127) |
| N-acetylglucosamine/N-acetylgalactosamine | Carbohydrate | Aminosugar Metabolism |  |
| N-acetylglucosaminylasparagine | Carbohydrate | Aminosugar Metabolism | [HMDB00489](http://www.hmdb.ca/metabolites/HMDB00489) |
| N-acetylneuraminate | Carbohydrate | Aminosugar Metabolism | [HMDB00230](http://www.hmdb.ca/metabolites/HMDB00230) |
| lactose | Carbohydrate | Disaccharides and Oligosaccharides | [HMDB00186](http://www.hmdb.ca/metabolites/HMDB00186) |
| sucrose | Carbohydrate | Disaccharides and Oligosaccharides | [HMDB00258](http://www.hmdb.ca/metabolites/HMDB00258) |
| fructose | Carbohydrate | Fructose, Mannose and Galactose Metabolism | [HMDB00660](http://www.hmdb.ca/metabolites/HMDB00660) |
| galactonate | Carbohydrate | Fructose, Mannose and Galactose Metabolism | [HMDB00565](http://www.hmdb.ca/metabolites/HMDB00565) |
| mannitol/sorbitol | Carbohydrate | Fructose, Mannose and Galactose Metabolism | [HMDB00247](http://www.hmdb.ca/metabolites/HMDB00247) |
| mannose | Carbohydrate | Fructose, Mannose and Galactose Metabolism | [HMDB00169](http://www.hmdb.ca/metabolites/HMDB00169) |
| maltose | Carbohydrate | Glycogen Metabolism | [HMDB00163](http://www.hmdb.ca/metabolites/HMDB00163) |
| maltotetraose | Carbohydrate | Glycogen Metabolism | [HMDB01296](http://www.hmdb.ca/metabolites/HMDB01296) |
| maltotriose | Carbohydrate | Glycogen Metabolism | [HMDB01262](http://www.hmdb.ca/metabolites/HMDB01262) |
| 1,5-anhydroglucitol (1,5-AG) | Carbohydrate | Glycolysis, Gluconeogenesis, and Pyruvate Metabolism | [HMDB02712](http://www.hmdb.ca/metabolites/HMDB02712) |
| 3-phosphoglycerate | Carbohydrate | Glycolysis, Gluconeogenesis, and Pyruvate Metabolism | [HMDB00807](http://www.hmdb.ca/metabolites/HMDB00807) |
| glucose | Carbohydrate | Glycolysis, Gluconeogenesis, and Pyruvate Metabolism | [HMDB00122](http://www.hmdb.ca/metabolites/HMDB00122) |
| glycerate | Carbohydrate | Glycolysis, Gluconeogenesis, and Pyruvate Metabolism | [HMDB00139](http://www.hmdb.ca/metabolites/HMDB00139) |
| lactate | Carbohydrate | Glycolysis, Gluconeogenesis, and Pyruvate Metabolism | [HMDB00190](http://www.hmdb.ca/metabolites/HMDB00190) |
| pyruvate | Carbohydrate | Glycolysis, Gluconeogenesis, and Pyruvate Metabolism | [HMDB00243](http://www.hmdb.ca/metabolites/HMDB00243) |
| arabinose | Carbohydrate | Pentose Metabolism | [HMDB00646](http://www.hmdb.ca/metabolites/HMDB00646) |
| arabitol/xylitol | Carbohydrate | Pentose Metabolism |  |
| arabonate/xylonate | Carbohydrate | Pentose Metabolism |  |
| ribitol | Carbohydrate | Pentose Metabolism | [HMDB00508](http://www.hmdb.ca/metabolites/HMDB00508) |
| ribonate | Carbohydrate | Pentose Metabolism | [HMDB00867](http://www.hmdb.ca/metabolites/HMDB00867) |
| ribulonate/xylulonate/lyxonate* | Carbohydrate | Pentose Metabolism |  |
| sedoheptulose | Carbohydrate | Pentose Metabolism | [HMDB03219](http://www.hmdb.ca/metabolites/HMDB03219) |
| xylose | Carbohydrate | Pentose Metabolism | [HMDB00098](http://www.hmdb.ca/metabolites/HMDB00098) |
| 2-O-methylascorbic acid | Cofactors and Vitamins | Ascorbate and Aldarate Metabolism |  |
| ascorbic acid 2-sulfate | Cofactors and Vitamins | Ascorbate and Aldarate Metabolism |  |
| ascorbic acid 3-sulfate* | Cofactors and Vitamins | Ascorbate and Aldarate Metabolism |  |
| gulonate* | Cofactors and Vitamins | Ascorbate and Aldarate Metabolism | [HMDB03290](http://www.hmdb.ca/metabolites/HMDB03290) |
| oxalate (ethanedioate) | Cofactors and Vitamins | Ascorbate and Aldarate Metabolism | [HMDB02329](http://www.hmdb.ca/metabolites/HMDB02329) |
| threonate | Cofactors and Vitamins | Ascorbate and Aldarate Metabolism | [HMDB00943](http://www.hmdb.ca/metabolites/HMDB00943) |
| bilirubin | Cofactors and Vitamins | Hemoglobin and Porphyrin Metabolism | [HMDB00054](http://www.hmdb.ca/metabolites/HMDB00054) |
| bilirubin (E,E)* | Cofactors and Vitamins | Hemoglobin and Porphyrin Metabolism |  |
| bilirubin (E,Z or Z,E)* | Cofactors and Vitamins | Hemoglobin and Porphyrin Metabolism | [HMDB00488](http://www.hmdb.ca/metabolites/HMDB00488) |
| biliverdin | Cofactors and Vitamins | Hemoglobin and Porphyrin Metabolism | [HMDB01008](http://www.hmdb.ca/metabolites/HMDB01008) |
| heme | Cofactors and Vitamins | Hemoglobin and Porphyrin Metabolism | [HMDB03178](http://www.hmdb.ca/metabolites/HMDB03178) |
| I-urobilinogen | Cofactors and Vitamins | Hemoglobin and Porphyrin Metabolism | [HMDB04157](http://www.hmdb.ca/metabolites/HMDB04157) |
| L-urobilin | Cofactors and Vitamins | Hemoglobin and Porphyrin Metabolism | [HMDB04159](http://www.hmdb.ca/metabolites/HMDB04159) |
| 1-methylnicotinamide | Cofactors and Vitamins | Nicotinate and Nicotinamide Metabolism | [HMDB00699](http://www.hmdb.ca/metabolites/HMDB00699) |
| N1-Methyl-2-pyridone-5-carboxamide | Cofactors and Vitamins | Nicotinate and Nicotinamide Metabolism | [HMDB04193](http://www.hmdb.ca/metabolites/HMDB04193) |
| N1-Methyl-4-pyridone-3-carboxamide | Cofactors and Vitamins | Nicotinate and Nicotinamide Metabolism | [HMDB04194](http://www.hmdb.ca/metabolites/HMDB04194) |
| nicotinamide | Cofactors and Vitamins | Nicotinate and Nicotinamide Metabolism | [HMDB01406](http://www.hmdb.ca/metabolites/HMDB01406) |
| nicotinate ribonucleoside | Cofactors and Vitamins | Nicotinate and Nicotinamide Metabolism | [HMDB06809](http://www.hmdb.ca/metabolites/HMDB06809) |
| quinolinate | Cofactors and Vitamins | Nicotinate and Nicotinamide Metabolism | [HMDB00232](http://www.hmdb.ca/metabolites/HMDB00232) |
| trigonelline (N'-methylnicotinate) | Cofactors and Vitamins | Nicotinate and Nicotinamide Metabolism | [HMDB00875](http://www.hmdb.ca/metabolites/HMDB00875) |
| pantoate | Cofactors and Vitamins | Pantothenate and CoA Metabolism |  |
| pantothenate (Vitamin B5) | Cofactors and Vitamins | Pantothenate and CoA Metabolism | [HMDB00210](http://www.hmdb.ca/metabolites/HMDB00210) |
| FAD | Cofactors and Vitamins | Riboflavin Metabolism | [HMDB01248](http://www.hmdb.ca/metabolites/HMDB01248) |
| alpha-CEHC glucuronide* | Cofactors and Vitamins | Tocopherol Metabolism |  |
| alpha-CEHC sulfate | Cofactors and Vitamins | Tocopherol Metabolism |  |
| alpha-CMBHC glucuronide | Cofactors and Vitamins | Tocopherol Metabolism |  |
| alpha-tocopherol | Cofactors and Vitamins | Tocopherol Metabolism | [HMDB01893](http://www.hmdb.ca/metabolites/HMDB01893) |
| delta-CEHC | Cofactors and Vitamins | Tocopherol Metabolism |  |
| delta-CEHC glucuronide | Cofactors and Vitamins | Tocopherol Metabolism |  |
| gamma-CEHC | Cofactors and Vitamins | Tocopherol Metabolism | [HMDB01931](http://www.hmdb.ca/metabolites/HMDB01931) |
| gamma-CEHC glucuronide* | Cofactors and Vitamins | Tocopherol Metabolism |  |
| gamma-tocopherol/beta-tocopherol | Cofactors and Vitamins | Tocopherol Metabolism |  |
| 4-oxo-retinoic acid | Cofactors and Vitamins | Vitamin A Metabolism | [HMDB06285](http://www.hmdb.ca/metabolites/HMDB06285) |
| beta-cryptoxanthin | Cofactors and Vitamins | Vitamin A Metabolism | [HMDB33844](http://www.hmdb.ca/metabolites/HMDB33844) |
| carotene diol (1) | Cofactors and Vitamins | Vitamin A Metabolism |  |
| carotene diol (2) | Cofactors and Vitamins | Vitamin A Metabolism |  |
| carotene diol (3) | Cofactors and Vitamins | Vitamin A Metabolism |  |
| retinal | Cofactors and Vitamins | Vitamin A Metabolism | [HMDB01358](http://www.hmdb.ca/metabolites/HMDB01358) |
| retinol (Vitamin A) | Cofactors and Vitamins | Vitamin A Metabolism | [HMDB00305](http://www.hmdb.ca/metabolites/HMDB00305) |
| pyridoxate | Cofactors and Vitamins | Vitamin B6 Metabolism | [HMDB00017](http://www.hmdb.ca/metabolites/HMDB00017) |
| phosphate | Energy | Oxidative Phosphorylation | [HMDB01429](http://www.hmdb.ca/metabolites/HMDB01429) |
| aconitate [cis or trans] | Energy | TCA Cycle |  |
| alpha-ketoglutarate | Energy | TCA Cycle | [HMDB00208](http://www.hmdb.ca/metabolites/HMDB00208) |
| citraconate/glutaconate | Energy | TCA Cycle |  |
| citrate | Energy | TCA Cycle | [HMDB00094](http://www.hmdb.ca/metabolites/HMDB00094) |
| fumarate | Energy | TCA Cycle | [HMDB00134](http://www.hmdb.ca/metabolites/HMDB00134) |
| malate | Energy | TCA Cycle | [HMDB00156](http://www.hmdb.ca/metabolites/HMDB00156) |
| succinate | Energy | TCA Cycle | [HMDB00254](http://www.hmdb.ca/metabolites/HMDB00254) |
| succinylcarnitine (C4-DC) | Energy | TCA Cycle | [HMDB61717](http://www.hmdb.ca/metabolites/HMDB61717) |
| 11beta-hydroxyandrosterone glucuronide | Lipid | Androgenic Steroids |  |
| 11-ketoetiocholanolone glucuronide | Lipid | Androgenic Steroids |  |
| 16a-hydroxy DHEA 3-sulfate | Lipid | Androgenic Steroids |  |
| 5alpha-androstan-3alpha,17beta-diol disulfate | Lipid | Androgenic Steroids |  |
| 5alpha-androstan-3alpha,17beta-diol monosulfate (1) | Lipid | Androgenic Steroids |  |
| 5alpha-androstan-3alpha,17beta-diol monosulfate (2) | Lipid | Androgenic Steroids |  |
| 5alpha-androstan-3beta,17alpha-diol disulfate | Lipid | Androgenic Steroids |  |
| 5alpha-androstan-3beta,17beta-diol disulfate | Lipid | Androgenic Steroids | [HMDB00493](http://www.hmdb.ca/metabolites/HMDB00493) |
| 5alpha-androstan-3beta,17beta-diol monosulfate (2) | Lipid | Androgenic Steroids |  |
| andro steroid monosulfate C19H28O6S (1)* | Lipid | Androgenic Steroids |  |
| androstenediol (3alpha, 17alpha) monosulfate (2) | Lipid | Androgenic Steroids |  |
| androstenediol (3alpha, 17alpha) monosulfate (3) | Lipid | Androgenic Steroids |  |
| androstenediol (3beta,17beta) disulfate (1) | Lipid | Androgenic Steroids | [HMDB03818](http://www.hmdb.ca/metabolites/HMDB03818) |
| androstenediol (3beta,17beta) disulfate (2) | Lipid | Androgenic Steroids |  |
| androstenediol (3beta,17beta) monosulfate (1) | Lipid | Androgenic Steroids | [HMDB03818](http://www.hmdb.ca/metabolites/HMDB03818) |
| androstenediol (3beta,17beta) monosulfate (2) | Lipid | Androgenic Steroids |  |
| androsterone glucuronide | Lipid | Androgenic Steroids | [HMDB02829](http://www.hmdb.ca/metabolites/HMDB02829) |
| androsterone sulfate | Lipid | Androgenic Steroids | [HMDB02759](http://www.hmdb.ca/metabolites/HMDB02759) |
| dehydroepiandrosterone sulfate (DHEA-S) | Lipid | Androgenic Steroids | [HMDB01032](http://www.hmdb.ca/metabolites/HMDB01032) |
| epiandrosterone sulfate | Lipid | Androgenic Steroids |  |
| etiocholanolone glucuronide | Lipid | Androgenic Steroids | [HMDB04484](http://www.hmdb.ca/metabolites/HMDB04484) |
| testosterone sulfate | Lipid | Androgenic Steroids | [HMDB02833](http://www.hmdb.ca/metabolites/HMDB02833) |
| carnitine | Lipid | Carnitine Metabolism | [HMDB00062](http://www.hmdb.ca/metabolites/HMDB00062) |
| deoxycarnitine | Lipid | Carnitine Metabolism | [HMDB01161](http://www.hmdb.ca/metabolites/HMDB01161) |
| palmitoyl-sphingosine-phosphoethanolamine (d18:1/16:0) | Lipid | Ceramide PEs |  |
| ceramide (d16:1/24:1, d18:1/22:1)* | Lipid | Ceramides |  |
| ceramide (d18:1/14:0, d16:1/16:0)* | Lipid | Ceramides |  |
| ceramide (d18:1/17:0, d17:1/18:0)* | Lipid | Ceramides |  |
| N-behenoyl-sphingadienine (d18:2/22:0)* | Lipid | Ceramides |  |
| N-nervonoyl-sphingadiene (d18:2/24:1)* | Lipid | Ceramides |  |
| N-palmitoyl-heptadecasphingosine (d17:1/16:0)* | Lipid | Ceramides |  |
| N-palmitoyl-sphingadienine (d18:2/16:0)* | Lipid | Ceramides |  |
| N-palmitoyl-sphingosine (d18:1/16:0) | Lipid | Ceramides | [HMDB04949](http://www.hmdb.ca/metabolites/HMDB04949) |
| N-stearoyl-sphingadienine (d18:2/18:0)* | Lipid | Ceramides |  |
| N-stearoyl-sphingosine (d18:1/18:0)* | Lipid | Ceramides | [HMDB04950](http://www.hmdb.ca/metabolites/HMDB04950) |
| corticosterone | Lipid | Corticosteroids | [HMDB01547](http://www.hmdb.ca/metabolites/HMDB01547) |
| cortisol | Lipid | Corticosteroids | [HMDB00063](http://www.hmdb.ca/metabolites/HMDB00063) |
| cortisone | Lipid | Corticosteroids | [HMDB02802](http://www.hmdb.ca/metabolites/HMDB02802) |
| cortolone glucuronide (1) | Lipid | Corticosteroids |  |
| tetrahydrocortisol sulfate (1) | Lipid | Corticosteroids |  |
| linoleoyl-arachidonoyl-glycerol (18:2/20:4) [1]* | Lipid | Diacylglycerol |  |
| linoleoyl-arachidonoyl-glycerol (18:2/20:4) [2]* | Lipid | Diacylglycerol |  |
| linoleoyl-linolenoyl-glycerol (18:2/18:3) [2]* | Lipid | Diacylglycerol |  |
| linoleoyl-linoleoyl-glycerol (18:2/18:2) [1]* | Lipid | Diacylglycerol |  |
| linoleoyl-linoleoyl-glycerol (18:2/18:2) [2]* | Lipid | Diacylglycerol |  |
| oleoyl-arachidonoyl-glycerol (18:1/20:4) [1]* | Lipid | Diacylglycerol |  |
| oleoyl-arachidonoyl-glycerol (18:1/20:4) [2]* | Lipid | Diacylglycerol |  |
| oleoyl-linoleoyl-glycerol (18:1/18:2) [1] | Lipid | Diacylglycerol | [HMDB07219](http://www.hmdb.ca/metabolites/HMDB07219) |
| oleoyl-linoleoyl-glycerol (18:1/18:2) [2] | Lipid | Diacylglycerol | [HMDB07219](http://www.hmdb.ca/metabolites/HMDB07219) |
| oleoyl-oleoyl-glycerol (18:1/18:1) [1]* | Lipid | Diacylglycerol |  |
| oleoyl-oleoyl-glycerol (18:1/18:1) [2]* | Lipid | Diacylglycerol |  |
| palmitoleoyl-linoleoyl-glycerol (16:1/18:2) [1]* | Lipid | Diacylglycerol |  |
| palmitoyl-arachidonoyl-glycerol (16:0/20:4) [1]* | Lipid | Diacylglycerol |  |
| palmitoyl-arachidonoyl-glycerol (16:0/20:4) [2]* | Lipid | Diacylglycerol |  |
| palmitoyl-linoleoyl-glycerol (16:0/18:2) [1]* | Lipid | Diacylglycerol | [HMDB07103](http://www.hmdb.ca/metabolites/HMDB07103) |
| palmitoyl-linoleoyl-glycerol (16:0/18:2) [2]* | Lipid | Diacylglycerol |  |
| palmitoyl-oleoyl-glycerol (16:0/18:1) [1]* | Lipid | Diacylglycerol |  |
| palmitoyl-oleoyl-glycerol (16:0/18:1) [2]* | Lipid | Diacylglycerol |  |
| N-palmitoyl-sphinganine (d18:0/16:0) | Lipid | Dihydroceramides | [HMDB11760](http://www.hmdb.ca/metabolites/HMDB11760) |
| N-stearoyl-sphinganine (d18:0/18:0)* | Lipid | Dihydroceramides |  |
| behenoyl dihydrosphingomyelin (d18:0/22:0)* | Lipid | Dihydrosphingomyelins | [HMDB12091](http://www.hmdb.ca/metabolites/HMDB12091) |
| myristoyl dihydrosphingomyelin (d18:0/14:0)* | Lipid | Dihydrosphingomyelins | [HMDB12085](http://www.hmdb.ca/metabolites/HMDB12085) |
| palmitoyl dihydrosphingomyelin (d18:0/16:0)* | Lipid | Dihydrosphingomyelins |  |
| sphingomyelin (d18:0/18:0, d19:0/17:0)* | Lipid | Dihydrosphingomyelins | [HMDB12087](http://www.hmdb.ca/metabolites/HMDB12087) |
| sphingomyelin (d18:0/20:0, d16:0/22:0)* | Lipid | Dihydrosphingomyelins |  |
| 15-HETE | Lipid | Eicosanoid | [HMDB02110](http://www.hmdb.ca/metabolites/HMDB02110) |
| 5-HEPE | Lipid | Eicosanoid | [HMDB05081](http://www.hmdb.ca/metabolites/HMDB05081) |
| 5-HETE | Lipid | Eicosanoid | [HMDB11134](http://www.hmdb.ca/metabolites/HMDB11134) |
| 5-KETE | Lipid | Eicosanoid | [HMDB10217](http://www.hmdb.ca/metabolites/HMDB10217) |
| leukotriene B4 | Lipid | Eicosanoid | [HMDB01085](http://www.hmdb.ca/metabolites/HMDB01085) |
| leukotriene B5 | Lipid | Eicosanoid | [HMDB05073](http://www.hmdb.ca/metabolites/HMDB05073) |
| linoleoyl ethanolamide | Lipid | Endocannabinoid | [HMDB12252](http://www.hmdb.ca/metabolites/HMDB12252) |
| N-linoleoyltaurine* | Lipid | Endocannabinoid |  |
| N-oleoylserine | Lipid | Endocannabinoid |  |
| N-oleoyltaurine | Lipid | Endocannabinoid |  |
| N-stearoylserine* | Lipid | Endocannabinoid |  |
| N-stearoyltaurine | Lipid | Endocannabinoid |  |
| oleoyl ethanolamide | Lipid | Endocannabinoid | [HMDB02088](http://www.hmdb.ca/metabolites/HMDB02088) |
| palmitoyl ethanolamide | Lipid | Endocannabinoid | [HMDB02100](http://www.hmdb.ca/metabolites/HMDB02100) |
| estrone 3-sulfate | Lipid | Estrogenic Steroids | [HMDB01425](http://www.hmdb.ca/metabolites/HMDB01425) |
| adipoylcarnitine (C6-DC) | Lipid | Fatty Acid Metabolism (Acyl Carnitine, Dicarboxylate) | [HMDB61677](http://www.hmdb.ca/metabolites/HMDB61677) |
| octadecanedioylcarnitine (C18-DC)* | Lipid | Fatty Acid Metabolism (Acyl Carnitine, Dicarboxylate) |  |
| octadecenedioylcarnitine (C18:1-DC)* | Lipid | Fatty Acid Metabolism (Acyl Carnitine, Dicarboxylate) |  |
| pimeloylcarnitine/3-methyladipoylcarnitine (C7-DC) | Lipid | Fatty Acid Metabolism (Acyl Carnitine, Dicarboxylate) |  |
| (R)-3-hydroxybutyrylcarnitine | Lipid | Fatty Acid Metabolism (Acyl Carnitine, Hydroxy) | [HMDB13127](http://www.hmdb.ca/metabolites/HMDB13127) |
| (S)-3-hydroxybutyrylcarnitine | Lipid | Fatty Acid Metabolism (Acyl Carnitine, Hydroxy) |  |
| 3-hydroxydecanoylcarnitine | Lipid | Fatty Acid Metabolism (Acyl Carnitine, Hydroxy) | [HMDB61636](http://www.hmdb.ca/metabolites/HMDB61636) |
| 3-hydroxyhexanoylcarnitine (1) | Lipid | Fatty Acid Metabolism (Acyl Carnitine, Hydroxy) |  |
| 3-hydroxyoleoylcarnitine | Lipid | Fatty Acid Metabolism (Acyl Carnitine, Hydroxy) |  |
| arachidoylcarnitine (C20)* | Lipid | Fatty Acid Metabolism (Acyl Carnitine, Long Chain Saturated) | [HMDB06460](http://www.hmdb.ca/metabolites/HMDB06460) |
| behenoylcarnitine (C22)* | Lipid | Fatty Acid Metabolism (Acyl Carnitine, Long Chain Saturated) |  |
| cerotoylcarnitine (C26)* | Lipid | Fatty Acid Metabolism (Acyl Carnitine, Long Chain Saturated) | [HMDB06347](http://www.hmdb.ca/metabolites/HMDB06347) |
| lignoceroylcarnitine (C24)* | Lipid | Fatty Acid Metabolism (Acyl Carnitine, Long Chain Saturated) |  |
| margaroylcarnitine (C17)* | Lipid | Fatty Acid Metabolism (Acyl Carnitine, Long Chain Saturated) | [HMDB06210](http://www.hmdb.ca/metabolites/HMDB06210) |
| myristoylcarnitine (C14) | Lipid | Fatty Acid Metabolism (Acyl Carnitine, Long Chain Saturated) | [HMDB05066](http://www.hmdb.ca/metabolites/HMDB05066) |
| palmitoylcarnitine (C16) | Lipid | Fatty Acid Metabolism (Acyl Carnitine, Long Chain Saturated) | [HMDB00222](http://www.hmdb.ca/metabolites/HMDB00222) |
| stearoylcarnitine (C18) | Lipid | Fatty Acid Metabolism (Acyl Carnitine, Long Chain Saturated) | [HMDB00848](http://www.hmdb.ca/metabolites/HMDB00848) |
| 4-methylnonanoylcarnitine | Lipid | Fatty Acid Metabolism (Acyl Carnitine, Medium Chain) |  |
| decanoylcarnitine (C10) | Lipid | Fatty Acid Metabolism (Acyl Carnitine, Medium Chain) | [HMDB00651](http://www.hmdb.ca/metabolites/HMDB00651) |
| hexanoylcarnitine (C6) | Lipid | Fatty Acid Metabolism (Acyl Carnitine, Medium Chain) | [HMDB00705](http://www.hmdb.ca/metabolites/HMDB00705) |
| laurylcarnitine (C12) | Lipid | Fatty Acid Metabolism (Acyl Carnitine, Medium Chain) | [HMDB02250](http://www.hmdb.ca/metabolites/HMDB02250) |
| nonanoylcarnitine (C9) | Lipid | Fatty Acid Metabolism (Acyl Carnitine, Medium Chain) | [HMDB13288](http://www.hmdb.ca/metabolites/HMDB13288) |
| octanoylcarnitine (C8) | Lipid | Fatty Acid Metabolism (Acyl Carnitine, Medium Chain) | [HMDB00791](http://www.hmdb.ca/metabolites/HMDB00791) |
| 3,4-methyleneheptanoylcarnitine | Lipid | Fatty Acid Metabolism (Acyl Carnitine, Monounsaturated) |  |
| 3-decenoylcarnitine | Lipid | Fatty Acid Metabolism (Acyl Carnitine, Monounsaturated) |  |
| 5-dodecenoylcarnitine (C12:1) | Lipid | Fatty Acid Metabolism (Acyl Carnitine, Monounsaturated) | [HMDB13326](http://www.hmdb.ca/metabolites/HMDB13326) |
| cis-4-decenoylcarnitine (C10:1) | Lipid | Fatty Acid Metabolism (Acyl Carnitine, Monounsaturated) |  |
| eicosenoylcarnitine (C20:1)* | Lipid | Fatty Acid Metabolism (Acyl Carnitine, Monounsaturated) |  |
| myristoleoylcarnitine (C14:1)* | Lipid | Fatty Acid Metabolism (Acyl Carnitine, Monounsaturated) |  |
| nervonoylcarnitine (C24:1)* | Lipid | Fatty Acid Metabolism (Acyl Carnitine, Monounsaturated) |  |
| oleoylcarnitine (C18:1) | Lipid | Fatty Acid Metabolism (Acyl Carnitine, Monounsaturated) | [HMDB05065](http://www.hmdb.ca/metabolites/HMDB05065) |
| palmitoleoylcarnitine (C16:1)* | Lipid | Fatty Acid Metabolism (Acyl Carnitine, Monounsaturated) |  |
| undecenoylcarnitine (C11:1) | Lipid | Fatty Acid Metabolism (Acyl Carnitine, Monounsaturated) |  |
| ximenoylcarnitine (C26:1)* | Lipid | Fatty Acid Metabolism (Acyl Carnitine, Monounsaturated) |  |
| adrenoylcarnitine (C22:4)* | Lipid | Fatty Acid Metabolism (Acyl Carnitine, Polyunsaturated) |  |
| arachidonoylcarnitine (C20:4) | Lipid | Fatty Acid Metabolism (Acyl Carnitine, Polyunsaturated) |  |
| dihomo-linolenoylcarnitine (C20:3n3 or 6)* | Lipid | Fatty Acid Metabolism (Acyl Carnitine, Polyunsaturated) |  |
| dihomo-linoleoylcarnitine (C20:2)* | Lipid | Fatty Acid Metabolism (Acyl Carnitine, Polyunsaturated) |  |
| docosahexaenoylcarnitine (C22:6)* | Lipid | Fatty Acid Metabolism (Acyl Carnitine, Polyunsaturated) |  |
| linolenoylcarnitine (C18:3)* | Lipid | Fatty Acid Metabolism (Acyl Carnitine, Polyunsaturated) |  |
| linoleoylcarnitine (C18:2)* | Lipid | Fatty Acid Metabolism (Acyl Carnitine, Polyunsaturated) | [HMDB06469](http://www.hmdb.ca/metabolites/HMDB06469) |
| acetylcarnitine (C2) | Lipid | Fatty Acid Metabolism (Acyl Carnitine, Short Chain) | [HMDB00201](http://www.hmdb.ca/metabolites/HMDB00201) |
| arachidonoylcholine | Lipid | Fatty Acid Metabolism (Acyl Choline) |  |
| dihomo-linolenoyl-choline | Lipid | Fatty Acid Metabolism (Acyl Choline) |  |
| docosahexaenoylcholine | Lipid | Fatty Acid Metabolism (Acyl Choline) |  |
| eicosapentaenoylcholine | Lipid | Fatty Acid Metabolism (Acyl Choline) |  |
| linoleoylcholine* | Lipid | Fatty Acid Metabolism (Acyl Choline) |  |
| oleoylcholine | Lipid | Fatty Acid Metabolism (Acyl Choline) |  |
| palmitoylcholine | Lipid | Fatty Acid Metabolism (Acyl Choline) |  |
| stearoylcholine* | Lipid | Fatty Acid Metabolism (Acyl Choline) |  |
| hexanoylglutamine | Lipid | Fatty Acid Metabolism (Acyl Glutamine) |  |
| 2-butenoylglycine | Lipid | Fatty Acid Metabolism (Acyl Glycine) |  |
| 3-hydroxybutyroylglycine | Lipid | Fatty Acid Metabolism (Acyl Glycine) |  |
| hexanoylglycine (C6) | Lipid | Fatty Acid Metabolism (Acyl Glycine) | [HMDB00701](http://www.hmdb.ca/metabolites/HMDB00701) |
| N-palmitoylglycine | Lipid | Fatty Acid Metabolism (Acyl Glycine) | [HMDB13034](http://www.hmdb.ca/metabolites/HMDB13034) |
| picolinoylglycine | Lipid | Fatty Acid Metabolism (Acyl Glycine) | [HMDB59766](http://www.hmdb.ca/metabolites/HMDB59766) |
| trans-2-hexenoylglycine | Lipid | Fatty Acid Metabolism (Acyl Glycine) |  |
| butyrylcarnitine (C4) | Lipid | Fatty Acid Metabolism (also BCAA Metabolism) | [HMDB02013](http://www.hmdb.ca/metabolites/HMDB02013) |
| methylmalonate (MMA) | Lipid | Fatty Acid Metabolism (also BCAA Metabolism) | [HMDB00202](http://www.hmdb.ca/metabolites/HMDB00202) |
| propionylcarnitine (C3) | Lipid | Fatty Acid Metabolism (also BCAA Metabolism) | [HMDB00824](http://www.hmdb.ca/metabolites/HMDB00824) |
| propionylglycine (C3) | Lipid | Fatty Acid Metabolism (also BCAA Metabolism) | [HMDB00783](http://www.hmdb.ca/metabolites/HMDB00783) |
| malonate | Lipid | Fatty Acid Synthesis | [HMDB00691](http://www.hmdb.ca/metabolites/HMDB00691) |
| malonylcarnitine | Lipid | Fatty Acid Synthesis | [HMDB02095](http://www.hmdb.ca/metabolites/HMDB02095) |
| heptadecenamide (17:1)* | Lipid | Fatty Acid, Amide |  |
| margaramide (17:0)* | Lipid | Fatty Acid, Amide |  |
| myristamide (14:0)* | Lipid | Fatty Acid, Amide |  |
| myristoleamide (14:1)* | Lipid | Fatty Acid, Amide |  |
| oleamide | Lipid | Fatty Acid, Amide | [HMDB02117](http://www.hmdb.ca/metabolites/HMDB02117) |
| pentadecanamide (15:0)* | Lipid | Fatty Acid, Amide |  |
| 2-aminoheptanoate | Lipid | Fatty Acid, Amino |  |
| 2-aminooctanoate | Lipid | Fatty Acid, Amino | [HMDB00991](http://www.hmdb.ca/metabolites/HMDB00991) |
| N-acetyl-2-aminooctanoate* | Lipid | Fatty Acid, Amino | [HMDB59745](http://www.hmdb.ca/metabolites/HMDB59745) |
| (14 or 15)-methylpalmitate (a17:0 or i17:0) | Lipid | Fatty Acid, Branched |  |
| (16 or 17)-methylstearate (a19:0 or i19:0) | Lipid | Fatty Acid, Branched | [HMDB37397](http://www.hmdb.ca/metabolites/HMDB37397) |
| 2-hydroxyphytanate* | Lipid | Fatty Acid, Branched |  |
| branched-chain fatty acid 18:0 (2) | Lipid | Fatty Acid, Branched |  |
| 2-hydroxyglutarate | Lipid | Fatty Acid, Dicarboxylate | [HMDB0059655](http://www.hmdb.ca/metabolites/HMDB0059655) |
| 2-hydroxysebacate | Lipid | Fatty Acid, Dicarboxylate | [HMDB00424](http://www.hmdb.ca/metabolites/HMDB00424) |
| 3-carboxy-4-methyl-5-pentyl-2-furanpropionate (3-CMPFP) | Lipid | Fatty Acid, Dicarboxylate |  |
| 3-carboxy-4-methyl-5-propyl-2-furanpropanoate (CMPF) | Lipid | Fatty Acid, Dicarboxylate | [HMDB61112](http://www.hmdb.ca/metabolites/HMDB61112) |
| 3-hydroxyadipate* | Lipid | Fatty Acid, Dicarboxylate | [HMDB00345](http://www.hmdb.ca/metabolites/HMDB00345) |
| 3-hydroxydodecanedioate* | Lipid | Fatty Acid, Dicarboxylate | [HMDB00413](http://www.hmdb.ca/metabolites/HMDB00413) |
| 3-methylglutarate/2-methylglutarate | Lipid | Fatty Acid, Dicarboxylate |  |
| adipate | Lipid | Fatty Acid, Dicarboxylate | [HMDB00448](http://www.hmdb.ca/metabolites/HMDB00448) |
| azelate (C9-DC) | Lipid | Fatty Acid, Dicarboxylate | [HMDB00784](http://www.hmdb.ca/metabolites/HMDB00784) |
| branched chain 14:0 dicarboxylic acid | Lipid | Fatty Acid, Dicarboxylate |  |
| decadienedioic acid (C10:2-DC) | Lipid | Fatty Acid, Dicarboxylate |  |
| docosadioate (C22-DC) | Lipid | Fatty Acid, Dicarboxylate |  |
| dodecadienoate (12:2)* | Lipid | Fatty Acid, Dicarboxylate |  |
| dodecanedioate (C12) | Lipid | Fatty Acid, Dicarboxylate | [HMDB00623](http://www.hmdb.ca/metabolites/HMDB00623) |
| dodecenedioate (C12:1-DC)* | Lipid | Fatty Acid, Dicarboxylate |  |
| eicosanedioate (C20-DC) | Lipid | Fatty Acid, Dicarboxylate |  |
| eicosenedioate (C20:1-DC)* | Lipid | Fatty Acid, Dicarboxylate |  |
| glutarate (C5-DC) | Lipid | Fatty Acid, Dicarboxylate | [HMDB00661](http://www.hmdb.ca/metabolites/HMDB00661) |
| heptenedioate (C7:1-DC)* | Lipid | Fatty Acid, Dicarboxylate |  |
| hexadecanedioate (C16) | Lipid | Fatty Acid, Dicarboxylate | [HMDB00672](http://www.hmdb.ca/metabolites/HMDB00672) |
| hexadecenedioate (C16:1-DC)* | Lipid | Fatty Acid, Dicarboxylate |  |
| hydroxy-CMPF* | Lipid | Fatty Acid, Dicarboxylate |  |
| maleate | Lipid | Fatty Acid, Dicarboxylate | [HMDB00176](http://www.hmdb.ca/metabolites/HMDB00176) |
| octadecadienedioate (C18:2-DC)* | Lipid | Fatty Acid, Dicarboxylate |  |
| octadecanedioate (C18) | Lipid | Fatty Acid, Dicarboxylate | [HMDB00782](http://www.hmdb.ca/metabolites/HMDB00782) |
| octadecenedioate (C18:1-DC) | Lipid | Fatty Acid, Dicarboxylate |  |
| pimelate (C7-DC) | Lipid | Fatty Acid, Dicarboxylate | [HMDB00857](http://www.hmdb.ca/metabolites/HMDB00857) |
| sebacate (C10-DC) | Lipid | Fatty Acid, Dicarboxylate | [HMDB00792](http://www.hmdb.ca/metabolites/HMDB00792) |
| suberate (C8-DC) | Lipid | Fatty Acid, Dicarboxylate | [HMDB00893](http://www.hmdb.ca/metabolites/HMDB00893) |
| tetradecadienedioate (C14:2-DC)* | Lipid | Fatty Acid, Dicarboxylate |  |
| tetradecanedioate (C14) | Lipid | Fatty Acid, Dicarboxylate | [HMDB00872](http://www.hmdb.ca/metabolites/HMDB00872) |
| tridecenedioate (C13:1-DC)* | Lipid | Fatty Acid, Dicarboxylate |  |
| undecanedioate (C11-DC) | Lipid | Fatty Acid, Dicarboxylate | [HMDB00888](http://www.hmdb.ca/metabolites/HMDB00888) |
| 12,13-DiHOME | Lipid | Fatty Acid, Dihydroxy | [HMDB04705](http://www.hmdb.ca/metabolites/HMDB04705) |
| 2R,3R-dihydroxybutyrate | Lipid | Fatty Acid, Dihydroxy | [HMDB00498](http://www.hmdb.ca/metabolites/HMDB00498) |
| 2S,3R-dihydroxybutyrate | Lipid | Fatty Acid, Dihydroxy | [HMDB02453](http://www.hmdb.ca/metabolites/HMDB02453) |
| 3,4-dihydroxybutyrate | Lipid | Fatty Acid, Dihydroxy |  |
| 9,10-DiHOME | Lipid | Fatty Acid, Dihydroxy | [HMDB04704](http://www.hmdb.ca/metabolites/HMDB04704) |
| 13-HODE + 9-HODE | Lipid | Fatty Acid, Monohydroxy |  |
| 16-hydroxypalmitate | Lipid | Fatty Acid, Monohydroxy | [HMDB06294](http://www.hmdb.ca/metabolites/HMDB06294) |
| 2-hydroxyarachidate* | Lipid | Fatty Acid, Monohydroxy |  |
| 2-hydroxybehenate | Lipid | Fatty Acid, Monohydroxy |  |
| 2-hydroxydecanoate | Lipid | Fatty Acid, Monohydroxy |  |
| 2-hydroxyheptanoate* | Lipid | Fatty Acid, Monohydroxy |  |
| 2-hydroxylaurate | Lipid | Fatty Acid, Monohydroxy |  |
| 2-hydroxynervonate* | Lipid | Fatty Acid, Monohydroxy |  |
| 2-hydroxyoctanoate | Lipid | Fatty Acid, Monohydroxy | [HMDB02264](http://www.hmdb.ca/metabolites/HMDB02264) |
| 2-hydroxypalmitate | Lipid | Fatty Acid, Monohydroxy | [HMDB31057](http://www.hmdb.ca/metabolites/HMDB31057) |
| 2-hydroxystearate | Lipid | Fatty Acid, Monohydroxy |  |
| 3-hydroxydecanoate | Lipid | Fatty Acid, Monohydroxy | [HMDB02203](http://www.hmdb.ca/metabolites/HMDB02203) |
| 3-hydroxyhexanoate | Lipid | Fatty Acid, Monohydroxy |  |
| 3-hydroxylaurate | Lipid | Fatty Acid, Monohydroxy | [HMDB00387](http://www.hmdb.ca/metabolites/HMDB00387) |
| 3-hydroxymyristate | Lipid | Fatty Acid, Monohydroxy |  |
| 3-hydroxyoctanoate | Lipid | Fatty Acid, Monohydroxy | [HMDB01954](http://www.hmdb.ca/metabolites/HMDB01954) |
| 3-hydroxysebacate | Lipid | Fatty Acid, Monohydroxy | [HMDB00350](http://www.hmdb.ca/metabolites/HMDB00350) |
| 5-hydroxyhexanoate | Lipid | Fatty Acid, Monohydroxy | [HMDB00525](http://www.hmdb.ca/metabolites/HMDB00525) |
| 8-hydroxyoctanoate | Lipid | Fatty Acid, Monohydroxy | [HMDB61914](http://www.hmdb.ca/metabolites/HMDB61914) |
| 9-hydroxystearate | Lipid | Fatty Acid, Monohydroxy | [HMDB61661](http://www.hmdb.ca/metabolites/HMDB61661) |
| alpha-hydroxycaproate | Lipid | Fatty Acid, Monohydroxy | [HMDB01624](http://www.hmdb.ca/metabolites/HMDB01624) |
| glycerol | Lipid | Glycerolipid Metabolism | [HMDB00131](http://www.hmdb.ca/metabolites/HMDB00131) |
| glycerol 3-phosphate | Lipid | Glycerolipid Metabolism | [HMDB00126](http://www.hmdb.ca/metabolites/HMDB00126) |
| glycerophosphoglycerol | Lipid | Glycerolipid Metabolism |  |
| glycosyl ceramide (d18:1/20:0, d16:1/22:0)* | Lipid | Hexosylceramides (HCER) |  |
| glycosyl ceramide (d18:2/24:1, d18:1/24:2)* | Lipid | Hexosylceramides (HCER) |  |
| glycosyl-N-(2-hydroxynervonoyl)-sphingosine (d18:1/24:1(2OH))* | Lipid | Hexosylceramides (HCER) |  |
| glycosyl-N-behenoyl-sphingadienine (d18:2/22:0)* | Lipid | Hexosylceramides (HCER) |  |
| glycosyl-N-palmitoyl-sphingosine (d18:1/16:0) | Lipid | Hexosylceramides (HCER) |  |
| glycosyl-N-stearoyl-sphingosine (d18:1/18:0) | Lipid | Hexosylceramides (HCER) |  |
| chiro-inositol | Lipid | Inositol Metabolism | [HMDB34220](http://www.hmdb.ca/metabolites/HMDB34220) |
| myo-inositol | Lipid | Inositol Metabolism | [HMDB00211](http://www.hmdb.ca/metabolites/HMDB00211) |
| 3-hydroxybutyrate (BHBA) | Lipid | Ketone Bodies | [HMDB00357](http://www.hmdb.ca/metabolites/HMDB00357) |
| lactosyl-N-behenoyl-sphingosine (d18:1/22:0)* | Lipid | Lactosylceramides (LCER) |  |
| lactosyl-N-nervonoyl-sphingosine (d18:1/24:1)* | Lipid | Lactosylceramides (LCER) |  |
| lactosyl-N-palmitoyl-sphingosine (d18:1/16:0) | Lipid | Lactosylceramides (LCER) |  |
| 10-heptadecenoate (17:1n7) | Lipid | Long Chain Monounsaturated Fatty Acid | [HMDB60038](http://www.hmdb.ca/metabolites/HMDB60038) |
| 10-nonadecenoate (19:1n9) | Lipid | Long Chain Monounsaturated Fatty Acid | [HMDB13622](http://www.hmdb.ca/metabolites/HMDB13622) |
| eicosenoate (20:1n9 or 1n11) | Lipid | Long Chain Monounsaturated Fatty Acid | [HMDB02231](http://www.hmdb.ca/metabolites/HMDB02231) |
| erucate (22:1n9) | Lipid | Long Chain Monounsaturated Fatty Acid | [HMDB02068](http://www.hmdb.ca/metabolites/HMDB02068) |
| myristoleate (14:1n5) | Lipid | Long Chain Monounsaturated Fatty Acid | [HMDB02000](http://www.hmdb.ca/metabolites/HMDB02000) |
| oleate/vaccenate (18:1) | Lipid | Long Chain Monounsaturated Fatty Acid |  |
| palmitoleate (16:1n7) | Lipid | Long Chain Monounsaturated Fatty Acid | [HMDB03229](http://www.hmdb.ca/metabolites/HMDB03229) |
| adrenate (22:4n6) | Lipid | Long Chain Polyunsaturated Fatty Acid (n3 and n6) | [HMDB02226](http://www.hmdb.ca/metabolites/HMDB02226) |
| arachidonate (20:4n6) | Lipid | Long Chain Polyunsaturated Fatty Acid (n3 and n6) | [HMDB01043](http://www.hmdb.ca/metabolites/HMDB01043) |
| dihomolinoleate (20:2n6) | Lipid | Long Chain Polyunsaturated Fatty Acid (n3 and n6) | [HMDB05060](http://www.hmdb.ca/metabolites/HMDB05060) |
| dihomolinolenate (20:3n3 or 3n6) | Lipid | Long Chain Polyunsaturated Fatty Acid (n3 and n6) | [HMDB02925](http://www.hmdb.ca/metabolites/HMDB02925) |
| docosadienoate (22:2n6) | Lipid | Long Chain Polyunsaturated Fatty Acid (n3 and n6) | [HMDB61714](http://www.hmdb.ca/metabolites/HMDB61714) |
| docosahexaenoate (DHA; 22:6n3) | Lipid | Long Chain Polyunsaturated Fatty Acid (n3 and n6) | [HMDB02183](http://www.hmdb.ca/metabolites/HMDB02183) |
| docosapentaenoate (DPA; 22:5n3) | Lipid | Long Chain Polyunsaturated Fatty Acid (n3 and n6) | [HMDB06528](http://www.hmdb.ca/metabolites/HMDB06528) |
| docosapentaenoate (n6 DPA; 22:5n6) | Lipid | Long Chain Polyunsaturated Fatty Acid (n3 and n6) | [HMDB01976](http://www.hmdb.ca/metabolites/HMDB01976) |
| docosatrienoate (22:3n3) | Lipid | Long Chain Polyunsaturated Fatty Acid (n3 and n6) | [HMDB02823](http://www.hmdb.ca/metabolites/HMDB02823) |
| docosatrienoate (22:3n6)* | Lipid | Long Chain Polyunsaturated Fatty Acid (n3 and n6) |  |
| eicosapentaenoate (EPA; 20:5n3) | Lipid | Long Chain Polyunsaturated Fatty Acid (n3 and n6) | [HMDB01999](http://www.hmdb.ca/metabolites/HMDB01999) |
| heneicosapentaenoate (21:5n3) | Lipid | Long Chain Polyunsaturated Fatty Acid (n3 and n6) |  |
| hexadecadienoate (16:2n6) | Lipid | Long Chain Polyunsaturated Fatty Acid (n3 and n6) | [HMDB00477](http://www.hmdb.ca/metabolites/HMDB00477) |
| linoleate (18:2n6) | Lipid | Long Chain Polyunsaturated Fatty Acid (n3 and n6) | [HMDB00673](http://www.hmdb.ca/metabolites/HMDB00673) |
| linolenate (18:3n3 or 3n6) | Lipid | Long Chain Polyunsaturated Fatty Acid (n3 and n6) | [HMDB03073](http://www.hmdb.ca/metabolites/HMDB03073) |
| nisinate (24:6n3) | Lipid | Long Chain Polyunsaturated Fatty Acid (n3 and n6) | [HMDB02007](http://www.hmdb.ca/metabolites/HMDB02007) |
| stearidonate (18:4n3) | Lipid | Long Chain Polyunsaturated Fatty Acid (n3 and n6) | [HMDB06547](http://www.hmdb.ca/metabolites/HMDB06547) |
| tetradecadienoate (14:2)* | Lipid | Long Chain Polyunsaturated Fatty Acid (n3 and n6) | [HMDB00560](http://www.hmdb.ca/metabolites/HMDB00560) |
| arachidate (20:0) | Lipid | Long Chain Saturated Fatty Acid | [HMDB02212](http://www.hmdb.ca/metabolites/HMDB02212) |
| margarate (17:0) | Lipid | Long Chain Saturated Fatty Acid | [HMDB02259](http://www.hmdb.ca/metabolites/HMDB02259) |
| myristate (14:0) | Lipid | Long Chain Saturated Fatty Acid | [HMDB00806](http://www.hmdb.ca/metabolites/HMDB00806) |
| nonadecanoate (19:0) | Lipid | Long Chain Saturated Fatty Acid | [HMDB00772](http://www.hmdb.ca/metabolites/HMDB00772) |
| palmitate (16:0) | Lipid | Long Chain Saturated Fatty Acid | [HMDB00220](http://www.hmdb.ca/metabolites/HMDB00220) |
| pentadecanoate (15:0) | Lipid | Long Chain Saturated Fatty Acid | [HMDB00826](http://www.hmdb.ca/metabolites/HMDB00826) |
| stearate (18:0) | Lipid | Long Chain Saturated Fatty Acid | [HMDB00827](http://www.hmdb.ca/metabolites/HMDB00827) |
| 1-arachidonoyl-GPA (20:4) | Lipid | Lysophospholipid |  |
| 1-arachidonoyl-GPC* (20:4)* | Lipid | Lysophospholipid | [HMDB10395](http://www.hmdb.ca/metabolites/HMDB10395) |
| 1-arachidonoyl-GPE (20:4n6)* | Lipid | Lysophospholipid | [HMDB11517](http://www.hmdb.ca/metabolites/HMDB11517) |
| 1-arachidonoyl-GPI* (20:4)* | Lipid | Lysophospholipid | [HMDB61690](http://www.hmdb.ca/metabolites/HMDB61690) |
| 1-cerotoyl-GPC (26:0)* | Lipid | Lysophospholipid | [HMDB29205](http://www.hmdb.ca/metabolites/HMDB29205) |
| 1-lignoceroyl-GPC (24:0) | Lipid | Lysophospholipid | [HMDB10405](http://www.hmdb.ca/metabolites/HMDB10405) |
| 1-linolenoyl-GPC (18:3)* | Lipid | Lysophospholipid | [HMDB10388](http://www.hmdb.ca/metabolites/HMDB10388) |
| 1-linoleoyl-GPA (18:2)* | Lipid | Lysophospholipid | [HMDB07856](http://www.hmdb.ca/metabolites/HMDB07856) |
| 1-linoleoyl-GPC (18:2) | Lipid | Lysophospholipid | [HMDB10386](http://www.hmdb.ca/metabolites/HMDB10386) |
| 1-linoleoyl-GPE (18:2)* | Lipid | Lysophospholipid | [HMDB11507](http://www.hmdb.ca/metabolites/HMDB11507) |
| 1-linoleoyl-GPG (18:2)* | Lipid | Lysophospholipid |  |
| 1-linoleoyl-GPI* (18:2)* | Lipid | Lysophospholipid |  |
| 1-oleoylglycerophosphate (18:1) | Lipid | Lysophospholipid |  |
| 1-oleoyl-GPC (18:1) | Lipid | Lysophospholipid | [HMDB02815](http://www.hmdb.ca/metabolites/HMDB02815) |
| 1-oleoyl-GPE (18:1) | Lipid | Lysophospholipid | [HMDB11506](http://www.hmdb.ca/metabolites/HMDB11506) |
| 1-oleoyl-GPI (18:1) | Lipid | Lysophospholipid |  |
| 1-oleoyl-GPS (18:1) | Lipid | Lysophospholipid | [HMDB61694](http://www.hmdb.ca/metabolites/HMDB61694) |
| 1-palmitoleoyl-GPC* (16:1)* | Lipid | Lysophospholipid | [HMDB10383](http://www.hmdb.ca/metabolites/HMDB10383) |
| 1-palmitoyl-GPA (16:0) | Lipid | Lysophospholipid | [HMDB00327](http://www.hmdb.ca/metabolites/HMDB00327) |
| 1-palmitoyl-GPC (16:0) | Lipid | Lysophospholipid | [HMDB10382](http://www.hmdb.ca/metabolites/HMDB10382) |
| 1-palmitoyl-GPE (16:0) | Lipid | Lysophospholipid | [HMDB11503](http://www.hmdb.ca/metabolites/HMDB11503) |
| 1-palmitoyl-GPG (16:0)* | Lipid | Lysophospholipid |  |
| 1-palmitoyl-GPI* (16:0) | Lipid | Lysophospholipid | [HMDB61695](http://www.hmdb.ca/metabolites/HMDB61695) |
| 1-stearoyl-GPC (18:0) | Lipid | Lysophospholipid | [HMDB10384](http://www.hmdb.ca/metabolites/HMDB10384) |
| 1-stearoyl-GPE (18:0) | Lipid | Lysophospholipid | [HMDB11130](http://www.hmdb.ca/metabolites/HMDB11130) |
| 1-stearoyl-GPG (18:0) | Lipid | Lysophospholipid |  |
| 1-stearoyl-GPI (18:0) | Lipid | Lysophospholipid | [HMDB61696](http://www.hmdb.ca/metabolites/HMDB61696) |
| 1-stearoyl-GPS (18:0)* | Lipid | Lysophospholipid |  |
| 2-palmitoyl-GPC* (16:0)* | Lipid | Lysophospholipid | [HMDB61702](http://www.hmdb.ca/metabolites/HMDB61702) |
| 2-stearoyl-GPE (18:0)* | Lipid | Lysophospholipid | [HMDB11129](http://www.hmdb.ca/metabolites/HMDB11129) |
| 1-(1-enyl-oleoyl)-GPE (P-18:1)* | Lipid | Lysoplasmalogen |  |
| 1-(1-enyl-palmitoyl)-GPC (P-16:0)* | Lipid | Lysoplasmalogen | [HMDB10407](http://www.hmdb.ca/metabolites/HMDB10407) |
| 1-(1-enyl-palmitoyl)-GPE (P-16:0)* | Lipid | Lysoplasmalogen |  |
| 1-(1-enyl-stearoyl)-GPE (P-18:0)* | Lipid | Lysoplasmalogen |  |
| (2 or 3)-decenoate (10:1n7 or n8) | Lipid | Medium Chain Fatty Acid |  |
| 10-undecenoate (11:1n1) | Lipid | Medium Chain Fatty Acid | [HMDB33724](http://www.hmdb.ca/metabolites/HMDB33724) |
| 5-dodecenoate (12:1n7) | Lipid | Medium Chain Fatty Acid | [HMDB00529](http://www.hmdb.ca/metabolites/HMDB00529) |
| caprate (10:0) | Lipid | Medium Chain Fatty Acid | [HMDB00511](http://www.hmdb.ca/metabolites/HMDB00511) |
| caproate (6:0) | Lipid | Medium Chain Fatty Acid | [HMDB00535](http://www.hmdb.ca/metabolites/HMDB00535) |
| caprylate (8:0) | Lipid | Medium Chain Fatty Acid | [HMDB00482](http://www.hmdb.ca/metabolites/HMDB00482) |
| cis-4-decenoate (10:1n6)* | Lipid | Medium Chain Fatty Acid |  |
| heptanoate (7:0) | Lipid | Medium Chain Fatty Acid | [HMDB00666](http://www.hmdb.ca/metabolites/HMDB00666) |
| laurate (12:0) | Lipid | Medium Chain Fatty Acid | [HMDB00638](http://www.hmdb.ca/metabolites/HMDB00638) |
| pelargonate (9:0) | Lipid | Medium Chain Fatty Acid | [HMDB00847](http://www.hmdb.ca/metabolites/HMDB00847) |
| 3-hydroxy-3-methylglutarate | Lipid | Mevalonate Metabolism | [HMDB00355](http://www.hmdb.ca/metabolites/HMDB00355) |
| 1-arachidonylglycerol (20:4) | Lipid | Monoacylglycerol | [HMDB11549](http://www.hmdb.ca/metabolites/HMDB11549) |
| 1-dihomo-linolenylglycerol (20:3) | Lipid | Monoacylglycerol |  |
| 1-docosahexaenoylglycerol (22:6) | Lipid | Monoacylglycerol | [HMDB11587](http://www.hmdb.ca/metabolites/HMDB11587) |
| 1-linolenoylglycerol (18:3) | Lipid | Monoacylglycerol | [HMDB11569](http://www.hmdb.ca/metabolites/HMDB11569) |
| 1-linoleoylglycerol (18:2) | Lipid | Monoacylglycerol |  |
| 1-myristoylglycerol (14:0) | Lipid | Monoacylglycerol | [HMDB11561](http://www.hmdb.ca/metabolites/HMDB11561) |
| 1-oleoylglycerol (18:1) | Lipid | Monoacylglycerol | [HMDB11567](http://www.hmdb.ca/metabolites/HMDB11567) |
| 1-palmitoleoylglycerol (16:1)* | Lipid | Monoacylglycerol |  |
| 1-palmitoylglycerol (16:0) | Lipid | Monoacylglycerol | [HMDB31074](http://www.hmdb.ca/metabolites/HMDB31074) |
| 2-linoleoylglycerol (18:2) | Lipid | Monoacylglycerol | [HMDB11538](http://www.hmdb.ca/metabolites/HMDB11538) |
| 2-oleoylglycerol (18:1) | Lipid | Monoacylglycerol | [HMDB11537](http://www.hmdb.ca/metabolites/HMDB11537) |
| 2-palmitoylglycerol (16:0) | Lipid | Monoacylglycerol | [HMDB11533](http://www.hmdb.ca/metabolites/HMDB11533) |
| 1,2-dilinoleoyl-GPC (18:2/18:2) | Lipid | Phosphatidylcholine (PC) | [HMDB08138](http://www.hmdb.ca/metabolites/HMDB08138) |
| 1,2-dipalmitoyl-GPC (16:0/16:0) | Lipid | Phosphatidylcholine (PC) | [HMDB00564](http://www.hmdb.ca/metabolites/HMDB00564) |
| 1-linoleoyl-2-arachidonoyl-GPC (18:2/20:4n6)* | Lipid | Phosphatidylcholine (PC) | [HMDB08147](http://www.hmdb.ca/metabolites/HMDB08147) |
| 1-linoleoyl-2-linolenoyl-GPC (18:2/18:3)* | Lipid | Phosphatidylcholine (PC) | [HMDB08141](http://www.hmdb.ca/metabolites/HMDB08141) |
| 1-myristoyl-2-arachidonoyl-GPC (14:0/20:4)* | Lipid | Phosphatidylcholine (PC) | [HMDB07883](http://www.hmdb.ca/metabolites/HMDB07883) |
| 1-myristoyl-2-palmitoyl-GPC (14:0/16:0) | Lipid | Phosphatidylcholine (PC) | [HMDB07869](http://www.hmdb.ca/metabolites/HMDB07869) |
| 1-oleoyl-2-docosahexaenoyl-GPC (18:1/22:6)* | Lipid | Phosphatidylcholine (PC) | [HMDB08123](http://www.hmdb.ca/metabolites/HMDB08123) |
| 1-palmitoleoyl-2-linolenoyl-GPC (16:1/18:3)* | Lipid | Phosphatidylcholine (PC) | [HMDB08008](http://www.hmdb.ca/metabolites/HMDB08008) |
| 1-palmitoleoyl-2-linoleoyl-GPC (16:1/18:2)* | Lipid | Phosphatidylcholine (PC) | [HMDB08006](http://www.hmdb.ca/metabolites/HMDB08006) |
| 1-palmitoyl-2-arachidonoyl-GPC (16:0/20:4n6) | Lipid | Phosphatidylcholine (PC) | [HMDB07982](http://www.hmdb.ca/metabolites/HMDB07982) |
| 1-palmitoyl-2-dihomo-linolenoyl-GPC (16:0/20:3n3 or 6)* | Lipid | Phosphatidylcholine (PC) |  |
| 1-palmitoyl-2-docosahexaenoyl-GPC (16:0/22:6) | Lipid | Phosphatidylcholine (PC) | [HMDB07991](http://www.hmdb.ca/metabolites/HMDB07991) |
| 1-palmitoyl-2-linoleoyl-GPC (16:0/18:2) | Lipid | Phosphatidylcholine (PC) | [HMDB07973](http://www.hmdb.ca/metabolites/HMDB07973) |
| 1-palmitoyl-2-oleoyl-GPC (16:0/18:1) | Lipid | Phosphatidylcholine (PC) | [HMDB07972](http://www.hmdb.ca/metabolites/HMDB07972) |
| 1-palmitoyl-2-palmitoleoyl-GPC (16:0/16:1)* | Lipid | Phosphatidylcholine (PC) |  |
| 1-palmitoyl-2-stearoyl-GPC (16:0/18:0) | Lipid | Phosphatidylcholine (PC) | [HMDB07970](http://www.hmdb.ca/metabolites/HMDB07970) |
| 1-stearoyl-2-arachidonoyl-GPC (18:0/20:4) | Lipid | Phosphatidylcholine (PC) | [HMDB08048](http://www.hmdb.ca/metabolites/HMDB08048) |
| 1-stearoyl-2-docosahexaenoyl-GPC (18:0/22:6) | Lipid | Phosphatidylcholine (PC) | [HMDB08057](http://www.hmdb.ca/metabolites/HMDB08057) |
| 1-stearoyl-2-linoleoyl-GPC (18:0/18:2)* | Lipid | Phosphatidylcholine (PC) |  |
| 1-stearoyl-2-oleoyl-GPC (18:0/18:1) | Lipid | Phosphatidylcholine (PC) | [HMDB08038](http://www.hmdb.ca/metabolites/HMDB08038) |
| 1,2-dipalmitoyl-GPE (16:0/16:0)* | Lipid | Phosphatidylethanolamine (PE) | [HMDB08923](http://www.hmdb.ca/metabolites/HMDB08923) |
| 1-oleoyl-2-docosahexaenoyl-GPE (18:1/22:6)* | Lipid | Phosphatidylethanolamine (PE) |  |
| 1-oleoyl-2-linoleoyl-GPE (18:1/18:2)* | Lipid | Phosphatidylethanolamine (PE) | [HMDB05349](http://www.hmdb.ca/metabolites/HMDB05349) |
| 1-palmitoyl-2-arachidonoyl-GPE (16:0/20:4)* | Lipid | Phosphatidylethanolamine (PE) | [HMDB05323](http://www.hmdb.ca/metabolites/HMDB05323) |
| 1-palmitoyl-2-docosahexaenoyl-GPE (16:0/22:6)* | Lipid | Phosphatidylethanolamine (PE) | [HMDB05324](http://www.hmdb.ca/metabolites/HMDB05324) |
| 1-palmitoyl-2-linoleoyl-GPE (16:0/18:2) | Lipid | Phosphatidylethanolamine (PE) | [HMDB05322](http://www.hmdb.ca/metabolites/HMDB05322) |
| 1-palmitoyl-2-oleoyl-GPE (16:0/18:1) | Lipid | Phosphatidylethanolamine (PE) | [HMDB05320](http://www.hmdb.ca/metabolites/HMDB05320) |
| 1-palmitoyl-2-stearoyl-GPE (16:0/18:0)* | Lipid | Phosphatidylethanolamine (PE) | [HMDB08925](http://www.hmdb.ca/metabolites/HMDB08925) |
| 1-stearoyl-2-arachidonoyl-GPE (18:0/20:4) | Lipid | Phosphatidylethanolamine (PE) | [HMDB09003](http://www.hmdb.ca/metabolites/HMDB09003) |
| 1-stearoyl-2-docosahexaenoyl-GPE (18:0/22:6)* | Lipid | Phosphatidylethanolamine (PE) | [HMDB05334](http://www.hmdb.ca/metabolites/HMDB05334) |
| 1-stearoyl-2-linoleoyl-GPE (18:0/18:2)* | Lipid | Phosphatidylethanolamine (PE) | [HMDB08994](http://www.hmdb.ca/metabolites/HMDB08994) |
| 1-stearoyl-2-oleoyl-GPE (18:0/18:1) | Lipid | Phosphatidylethanolamine (PE) | [HMDB08993](http://www.hmdb.ca/metabolites/HMDB08993) |
| 1-palmitoyl-2-arachidonoyl-GPI (16:0/20:4)* | Lipid | Phosphatidylinositol (PI) | [HMDB09789](http://www.hmdb.ca/metabolites/HMDB09789) |
| 1-palmitoyl-2-linoleoyl-GPI (16:0/18:2) | Lipid | Phosphatidylinositol (PI) | [HMDB09784](http://www.hmdb.ca/metabolites/HMDB09784) |
| 1-palmitoyl-2-oleoyl-GPI (16:0/18:1)* | Lipid | Phosphatidylinositol (PI) | [HMDB09783](http://www.hmdb.ca/metabolites/HMDB09783) |
| 1-stearoyl-2-arachidonoyl-GPI (18:0/20:4) | Lipid | Phosphatidylinositol (PI) | [HMDB09815](http://www.hmdb.ca/metabolites/HMDB09815) |
| 1-stearoyl-2-linoleoyl-GPI (18:0/18:2) | Lipid | Phosphatidylinositol (PI) | [HMDB09809](http://www.hmdb.ca/metabolites/HMDB09809) |
| 1-stearoyl-2-oleoyl-GPI (18:0/18:1)* | Lipid | Phosphatidylinositol (PI) |  |
| 1-stearoyl-2-arachidonoyl-GPS (18:0/20:4) | Lipid | Phosphatidylserine (PS) | [HMDB12383](http://www.hmdb.ca/metabolites/HMDB12383) |
| 1-stearoyl-2-oleoyl-GPS (18:0/18:1) | Lipid | Phosphatidylserine (PS) | [HMDB10163](http://www.hmdb.ca/metabolites/HMDB10163) |
| choline | Lipid | Phospholipid Metabolism | [HMDB00097](http://www.hmdb.ca/metabolites/HMDB00097) |
| glycerophosphoethanolamine | Lipid | Phospholipid Metabolism | [HMDB00114](http://www.hmdb.ca/metabolites/HMDB00114) |
| glycerophosphoinositol* | Lipid | Phospholipid Metabolism |  |
| glycerophosphorylcholine (GPC) | Lipid | Phospholipid Metabolism | [HMDB00086](http://www.hmdb.ca/metabolites/HMDB00086) |
| phosphocholine | Lipid | Phospholipid Metabolism | [HMDB01565](http://www.hmdb.ca/metabolites/HMDB01565) |
| phosphoethanolamine (PE) | Lipid | Phospholipid Metabolism | [HMDB00224](http://www.hmdb.ca/metabolites/HMDB00224) |
| trimethylamine N-oxide | Lipid | Phospholipid Metabolism | [HMDB00925](http://www.hmdb.ca/metabolites/HMDB00925) |
| 1-(1-enyl-palmitoyl)-2-arachidonoyl-GPC (P-16:0/20:4)* | Lipid | Plasmalogen | [HMDB11220](http://www.hmdb.ca/metabolites/HMDB11220) |
| 1-(1-enyl-palmitoyl)-2-arachidonoyl-GPE (P-16:0/20:4)* | Lipid | Plasmalogen | [HMDB11352](http://www.hmdb.ca/metabolites/HMDB11352) |
| 1-(1-enyl-palmitoyl)-2-linoleoyl-GPC (P-16:0/18:2)* | Lipid | Plasmalogen | [HMDB11211](http://www.hmdb.ca/metabolites/HMDB11211) |
| 1-(1-enyl-palmitoyl)-2-linoleoyl-GPE (P-16:0/18:2)* | Lipid | Plasmalogen | [HMDB11343](http://www.hmdb.ca/metabolites/HMDB11343) |
| 1-(1-enyl-palmitoyl)-2-oleoyl-GPC (P-16:0/18:1)* | Lipid | Plasmalogen |  |
| 1-(1-enyl-palmitoyl)-2-oleoyl-GPE (P-16:0/18:1)* | Lipid | Plasmalogen | [HMDB11342](http://www.hmdb.ca/metabolites/HMDB11342) |
| 1-(1-enyl-palmitoyl)-2-palmitoleoyl-GPC (P-16:0/16:1)* | Lipid | Plasmalogen | [HMDB11207](http://www.hmdb.ca/metabolites/HMDB11207) |
| 1-(1-enyl-palmitoyl)-2-palmitoyl-GPC (P-16:0/16:0)* | Lipid | Plasmalogen | [HMDB11206](http://www.hmdb.ca/metabolites/HMDB11206) |
| 1-(1-enyl-stearoyl)-2-arachidonoyl-GPE (P-18:0/20:4)* | Lipid | Plasmalogen | [HMDB05779](http://www.hmdb.ca/metabolites/HMDB05779) |
| 1-(1-enyl-stearoyl)-2-linoleoyl-GPE (P-18:0/18:2)* | Lipid | Plasmalogen | [HMDB11376](http://www.hmdb.ca/metabolites/HMDB11376) |
| 1-(1-enyl-stearoyl)-2-oleoyl-GPE (P-18:0/18:1) | Lipid | Plasmalogen | [HMDB11375](http://www.hmdb.ca/metabolites/HMDB11375) |
| 17alpha-hydroxypregnanolone glucuronide | Lipid | Pregnenolone Steroids |  |
| 17alpha-hydroxypregnenolone 3-sulfate | Lipid | Pregnenolone Steroids | [HMDB00416](http://www.hmdb.ca/metabolites/HMDB00416) |
| 21-hydroxypregnenolone disulfate | Lipid | Pregnenolone Steroids |  |
| pregnen-diol disulfate* | Lipid | Pregnenolone Steroids |  |
| pregnenediol sulfate (C21H34O5S)* | Lipid | Pregnenolone Steroids |  |
| pregnenetriol disulfate* | Lipid | Pregnenolone Steroids |  |
| pregnenetriol sulfate* | Lipid | Pregnenolone Steroids |  |
| pregnenolone sulfate | Lipid | Pregnenolone Steroids | [HMDB00774](http://www.hmdb.ca/metabolites/HMDB00774) |
| chenodeoxycholate | Lipid | Primary Bile Acid Metabolism | [HMDB00518](http://www.hmdb.ca/metabolites/HMDB00518) |
| chenodeoxycholic acid sulfate (1) | Lipid | Primary Bile Acid Metabolism |  |
| cholate | Lipid | Primary Bile Acid Metabolism | [HMDB00619](http://www.hmdb.ca/metabolites/HMDB00619) |
| cholic acid glucuronide | Lipid | Primary Bile Acid Metabolism |  |
| glyco-beta-muricholate | Lipid | Primary Bile Acid Metabolism |  |
| glycochenodeoxycholate | Lipid | Primary Bile Acid Metabolism | [HMDB00637](http://www.hmdb.ca/metabolites/HMDB00637) |
| glycochenodeoxycholate 3-sulfate | Lipid | Primary Bile Acid Metabolism |  |
| glycochenodeoxycholate glucuronide (1) | Lipid | Primary Bile Acid Metabolism |  |
| glycocholate | Lipid | Primary Bile Acid Metabolism | [HMDB00138](http://www.hmdb.ca/metabolites/HMDB00138) |
| glycocholate glucuronide (1) | Lipid | Primary Bile Acid Metabolism |  |
| glycocholate sulfate | Lipid | Primary Bile Acid Metabolism |  |
| tauro-beta-muricholate | Lipid | Primary Bile Acid Metabolism | [HMDB00932](http://www.hmdb.ca/metabolites/HMDB00932) |
| taurochenodeoxycholate | Lipid | Primary Bile Acid Metabolism | [HMDB00951](http://www.hmdb.ca/metabolites/HMDB00951) |
| taurocholate | Lipid | Primary Bile Acid Metabolism | [HMDB00036](http://www.hmdb.ca/metabolites/HMDB00036) |
| 5alpha-pregnan-3beta,20alpha-diol disulfate | Lipid | Progestin Steroids |  |
| 5alpha-pregnan-3beta,20alpha-diol monosulfate (2) | Lipid | Progestin Steroids |  |
| 5alpha-pregnan-3beta,20beta-diol monosulfate (1) | Lipid | Progestin Steroids |  |
| 5alpha-pregnan-diol disulfate | Lipid | Progestin Steroids |  |
| pregnanediol-3-glucuronide | Lipid | Progestin Steroids | [HMDB10318](http://www.hmdb.ca/metabolites/HMDB10318) |
| 3b-hydroxy-5-cholenoic acid | Lipid | Secondary Bile Acid Metabolism | [HMDB00308](http://www.hmdb.ca/metabolites/HMDB00308) |
| 7-ketodeoxycholate | Lipid | Secondary Bile Acid Metabolism | [HMDB00391](http://www.hmdb.ca/metabolites/HMDB00391) |
| deoxycholate | Lipid | Secondary Bile Acid Metabolism | [HMDB00626](http://www.hmdb.ca/metabolites/HMDB00626) |
| deoxycholic acid 12-sulfate* | Lipid | Secondary Bile Acid Metabolism |  |
| deoxycholic acid glucuronide | Lipid | Secondary Bile Acid Metabolism |  |
| glycocholenate sulfate* | Lipid | Secondary Bile Acid Metabolism |  |
| glycodeoxycholate | Lipid | Secondary Bile Acid Metabolism | [HMDB00631](http://www.hmdb.ca/metabolites/HMDB00631) |
| glycodeoxycholate 3-sulfate | Lipid | Secondary Bile Acid Metabolism |  |
| glycohyocholate | Lipid | Secondary Bile Acid Metabolism |  |
| glycolithocholate | Lipid | Secondary Bile Acid Metabolism | [HMDB00698](http://www.hmdb.ca/metabolites/HMDB00698) |
| glycolithocholate sulfate* | Lipid | Secondary Bile Acid Metabolism | [HMDB02639](http://www.hmdb.ca/metabolites/HMDB02639) |
| glycoursodeoxycholate | Lipid | Secondary Bile Acid Metabolism | [HMDB00708](http://www.hmdb.ca/metabolites/HMDB00708) |
| glycoursodeoxycholic acid sulfate (1) | Lipid | Secondary Bile Acid Metabolism |  |
| hyocholate | Lipid | Secondary Bile Acid Metabolism | [HMDB00760](http://www.hmdb.ca/metabolites/HMDB00760) |
| isoursodeoxycholate | Lipid | Secondary Bile Acid Metabolism | [HMDB00686](http://www.hmdb.ca/metabolites/HMDB00686) |
| isoursodeoxycholate sulfate (1) | Lipid | Secondary Bile Acid Metabolism |  |
| lithocholate sulfate (1) | Lipid | Secondary Bile Acid Metabolism |  |
| lithocholic acid sulfate (2) | Lipid | Secondary Bile Acid Metabolism |  |
| taurochenodeoxycholic acid 3-sulfate | Lipid | Secondary Bile Acid Metabolism | [HMDB02486](http://www.hmdb.ca/metabolites/HMDB02486) |
| taurocholenate sulfate* | Lipid | Secondary Bile Acid Metabolism |  |
| taurodeoxycholate | Lipid | Secondary Bile Acid Metabolism | [HMDB00896](http://www.hmdb.ca/metabolites/HMDB00896) |
| taurodeoxycholic acid 3-sulfate | Lipid | Secondary Bile Acid Metabolism |  |
| taurohyocholate* | Lipid | Secondary Bile Acid Metabolism |  |
| taurolithocholate 3-sulfate | Lipid | Secondary Bile Acid Metabolism | [HMDB02580](http://www.hmdb.ca/metabolites/HMDB02580) |
| tauroursodeoxycholate | Lipid | Secondary Bile Acid Metabolism | [HMDB00874](http://www.hmdb.ca/metabolites/HMDB00874) |
| tauroursodeoxycholic acid sulfate (1) | Lipid | Secondary Bile Acid Metabolism |  |
| ursodeoxycholate | Lipid | Secondary Bile Acid Metabolism | [HMDB00946](http://www.hmdb.ca/metabolites/HMDB00946) |
| butyrate/isobutyrate (4:0) | Lipid | Short Chain Fatty Acid | [HMDB00039](http://www.hmdb.ca/metabolites/HMDB00039) |
| valerate (5:0) | Lipid | Short Chain Fatty Acid | [HMDB00892](http://www.hmdb.ca/metabolites/HMDB00892) |
| sphingadienine | Lipid | Sphingolipid Synthesis |  |
| sphinganine | Lipid | Sphingolipid Synthesis | [HMDB00269](http://www.hmdb.ca/metabolites/HMDB00269) |
| sphinganine-1-phosphate | Lipid | Sphingolipid Synthesis | [HMDB01383](http://www.hmdb.ca/metabolites/HMDB01383) |
| behenoyl sphingomyelin (d18:1/22:0)* | Lipid | Sphingomyelins | [HMDB12103](http://www.hmdb.ca/metabolites/HMDB12103) |
| hydroxypalmitoyl sphingomyelin (d18:1/16:0(OH)) | Lipid | Sphingomyelins |  |
| lignoceroyl sphingomyelin (d18:1/24:0) | Lipid | Sphingomyelins |  |
| palmitoyl sphingomyelin (d18:1/16:0) | Lipid | Sphingomyelins |  |
| sphingomyelin (d17:1/14:0, d16:1/15:0)* | Lipid | Sphingomyelins |  |
| sphingomyelin (d17:1/16:0, d18:1/15:0, d16:1/17:0)* | Lipid | Sphingomyelins |  |
| sphingomyelin (d17:2/16:0, d18:2/15:0)* | Lipid | Sphingomyelins |  |
| sphingomyelin (d18:1/14:0, d16:1/16:0)* | Lipid | Sphingomyelins | [HMDB12097](http://www.hmdb.ca/metabolites/HMDB12097) |
| sphingomyelin (d18:1/17:0, d17:1/18:0, d19:1/16:0) | Lipid | Sphingomyelins |  |
| sphingomyelin (d18:1/18:1, d18:2/18:0) | Lipid | Sphingomyelins | [HMDB12101](http://www.hmdb.ca/metabolites/HMDB12101) |
| sphingomyelin (d18:1/19:0, d19:1/18:0)* | Lipid | Sphingomyelins |  |
| sphingomyelin (d18:1/20:0, d16:1/22:0)* | Lipid | Sphingomyelins | [HMDB12102](http://www.hmdb.ca/metabolites/HMDB12102) |
| sphingomyelin (d18:1/20:1, d18:2/20:0)* | Lipid | Sphingomyelins |  |
| sphingomyelin (d18:1/20:2, d18:2/20:1, d16:1/22:2)* | Lipid | Sphingomyelins |  |
| sphingomyelin (d18:1/21:0, d17:1/22:0, d16:1/23:0)* | Lipid | Sphingomyelins |  |
| sphingomyelin (d18:1/22:1, d18:2/22:0, d16:1/24:1)* | Lipid | Sphingomyelins | [HMDB12104](http://www.hmdb.ca/metabolites/HMDB12104) |
| sphingomyelin (d18:1/22:2, d18:2/22:1, d16:1/24:2)* | Lipid | Sphingomyelins |  |
| sphingomyelin (d18:1/24:1, d18:2/24:0)* | Lipid | Sphingomyelins | [HMDB12107](http://www.hmdb.ca/metabolites/HMDB12107) |
| sphingomyelin (d18:2/14:0, d18:1/14:1)* | Lipid | Sphingomyelins |  |
| sphingomyelin (d18:2/16:0, d18:1/16:1)* | Lipid | Sphingomyelins |  |
| sphingomyelin (d18:2/18:1)* | Lipid | Sphingomyelins |  |
| sphingomyelin (d18:2/21:0, d16:2/23:0)* | Lipid | Sphingomyelins |  |
| sphingomyelin (d18:2/23:0, d18:1/23:1, d17:1/24:1)* | Lipid | Sphingomyelins |  |
| sphingomyelin (d18:2/23:1)* | Lipid | Sphingomyelins |  |
| sphingomyelin (d18:2/24:1, d18:1/24:2)* | Lipid | Sphingomyelins |  |
| sphingomyelin (d18:2/24:2)* | Lipid | Sphingomyelins |  |
| stearoyl sphingomyelin (d18:1/18:0) | Lipid | Sphingomyelins | [HMDB01348](http://www.hmdb.ca/metabolites/HMDB01348) |
| tricosanoyl sphingomyelin (d18:1/23:0)* | Lipid | Sphingomyelins | [HMDB12105](http://www.hmdb.ca/metabolites/HMDB12105) |
| hexadecasphingosine (d16:1)* | Lipid | Sphingosines |  |
| sphingosine | Lipid | Sphingosines | [HMDB00252](http://www.hmdb.ca/metabolites/HMDB00252) |
| sphingosine 1-phosphate | Lipid | Sphingosines | [HMDB00277](http://www.hmdb.ca/metabolites/HMDB00277) |
| 25-hydroxycholesterol sulfate | Lipid | Sterol |  |
| 3beta,7alpha-dihydroxy-5-cholestenoate | Lipid | Sterol |  |
| 3beta-hydroxy-5-cholestenoate | Lipid | Sterol |  |
| 4-cholesten-3-one | Lipid | Sterol | [HMDB00921](http://www.hmdb.ca/metabolites/HMDB00921) |
| 7-HOCA | Lipid | Sterol | [HMDB12458](http://www.hmdb.ca/metabolites/HMDB12458) |
| 7-hydroxycholesterol (alpha or beta) | Lipid | Sterol | [HMDB06119](http://www.hmdb.ca/metabolites/HMDB06119) |
| campesterol | Lipid | Sterol | [HMDB02869](http://www.hmdb.ca/metabolites/HMDB02869) |
| cholesterol | Lipid | Sterol | [HMDB00067](http://www.hmdb.ca/metabolites/HMDB00067) |
| allantoin | Nucleotide | Purine Metabolism, (Hypo)Xanthine/Inosine containing | [HMDB00462](http://www.hmdb.ca/metabolites/HMDB00462) |
| hypoxanthine | Nucleotide | Purine Metabolism, (Hypo)Xanthine/Inosine containing | [HMDB00157](http://www.hmdb.ca/metabolites/HMDB00157) |
| inosine | Nucleotide | Purine Metabolism, (Hypo)Xanthine/Inosine containing | [HMDB00195](http://www.hmdb.ca/metabolites/HMDB00195) |
| inosine 5'-monophosphate (IMP) | Nucleotide | Purine Metabolism, (Hypo)Xanthine/Inosine containing | [HMDB00175](http://www.hmdb.ca/metabolites/HMDB00175) |
| N1-methylinosine | Nucleotide | Purine Metabolism, (Hypo)Xanthine/Inosine containing | [HMDB02721](http://www.hmdb.ca/metabolites/HMDB02721) |
| urate | Nucleotide | Purine Metabolism, (Hypo)Xanthine/Inosine containing | [HMDB00289](http://www.hmdb.ca/metabolites/HMDB00289) |
| xanthine | Nucleotide | Purine Metabolism, (Hypo)Xanthine/Inosine containing | [HMDB00292](http://www.hmdb.ca/metabolites/HMDB00292) |
| xanthosine | Nucleotide | Purine Metabolism, (Hypo)Xanthine/Inosine containing | [HMDB00299](http://www.hmdb.ca/metabolites/HMDB00299) |
| 1-methyladenosine | Nucleotide | Purine Metabolism, Adenine containing | [HMDB03331](http://www.hmdb.ca/metabolites/HMDB03331) |
| adenine | Nucleotide | Purine Metabolism, Adenine containing | [HMDB00034](http://www.hmdb.ca/metabolites/HMDB00034) |
| AMP | Nucleotide | Purine Metabolism, Adenine containing | [HMDB00045](http://www.hmdb.ca/metabolites/HMDB00045) |
| N6-carbamoylthreonyladenosine | Nucleotide | Purine Metabolism, Adenine containing | [HMDB41623](http://www.hmdb.ca/metabolites/HMDB41623) |
| N6-methyladenosine | Nucleotide | Purine Metabolism, Adenine containing | [HMDB04044](http://www.hmdb.ca/metabolites/HMDB04044) |
| N6-succinyladenosine | Nucleotide | Purine Metabolism, Adenine containing | [HMDB00912](http://www.hmdb.ca/metabolites/HMDB00912) |
| 7-methylguanine | Nucleotide | Purine Metabolism, Guanine containing | [HMDB00897](http://www.hmdb.ca/metabolites/HMDB00897) |
| N2,N2-dimethylguanosine | Nucleotide | Purine Metabolism, Guanine containing | [HMDB04824](http://www.hmdb.ca/metabolites/HMDB04824) |
| 2'-O-methylcytidine | Nucleotide | Pyrimidine Metabolism, Cytidine containing |  |
| cytidine | Nucleotide | Pyrimidine Metabolism, Cytidine containing | [HMDB00089](http://www.hmdb.ca/metabolites/HMDB00089) |
| cytosine | Nucleotide | Pyrimidine Metabolism, Cytidine containing | [HMDB00630](http://www.hmdb.ca/metabolites/HMDB00630) |
| N4-acetylcytidine | Nucleotide | Pyrimidine Metabolism, Cytidine containing | [HMDB05923](http://www.hmdb.ca/metabolites/HMDB05923) |
| orotate | Nucleotide | Pyrimidine Metabolism, Orotate containing | [HMDB00226](http://www.hmdb.ca/metabolites/HMDB00226) |
| orotidine | Nucleotide | Pyrimidine Metabolism, Orotate containing | [HMDB00788](http://www.hmdb.ca/metabolites/HMDB00788) |
| 3-aminoisobutyrate | Nucleotide | Pyrimidine Metabolism, Thymine containing | [HMDB03911](http://www.hmdb.ca/metabolites/HMDB03911) |
| 5,6-dihydrothymine | Nucleotide | Pyrimidine Metabolism, Thymine containing | [HMDB00079](http://www.hmdb.ca/metabolites/HMDB00079) |
| thymine | Nucleotide | Pyrimidine Metabolism, Thymine containing | [HMDB00262](http://www.hmdb.ca/metabolites/HMDB00262) |
| 2'-O-methyluridine | Nucleotide | Pyrimidine Metabolism, Uracil containing |  |
| 3-(3-amino-3-carboxypropyl)uridine* | Nucleotide | Pyrimidine Metabolism, Uracil containing |  |
| 3-ureidopropionate | Nucleotide | Pyrimidine Metabolism, Uracil containing | [HMDB00026](http://www.hmdb.ca/metabolites/HMDB00026) |
| 5,6-dihydrouridine | Nucleotide | Pyrimidine Metabolism, Uracil containing |  |
| 5-methyluridine (ribothymidine) | Nucleotide | Pyrimidine Metabolism, Uracil containing | [HMDB00884](http://www.hmdb.ca/metabolites/HMDB00884) |
| beta-alanine | Nucleotide | Pyrimidine Metabolism, Uracil containing | [HMDB00056](http://www.hmdb.ca/metabolites/HMDB00056) |
| N-acetyl-beta-alanine | Nucleotide | Pyrimidine Metabolism, Uracil containing |  |
| pseudouridine | Nucleotide | Pyrimidine Metabolism, Uracil containing | [HMDB00767](http://www.hmdb.ca/metabolites/HMDB00767) |
| uracil | Nucleotide | Pyrimidine Metabolism, Uracil containing | [HMDB00300](http://www.hmdb.ca/metabolites/HMDB00300) |
| uridine | Nucleotide | Pyrimidine Metabolism, Uracil containing | [HMDB00296](http://www.hmdb.ca/metabolites/HMDB00296) |
| 4-hydroxyphenylacetylglutamine | Peptide | Acetylated Peptides |  |
| phenylacetylcarnitine | Peptide | Acetylated Peptides |  |
| phenylacetylglutamate | Peptide | Acetylated Peptides | [HMDB59772](http://www.hmdb.ca/metabolites/HMDB59772) |
| phenylacetylglutamine | Peptide | Acetylated Peptides | [HMDB06344](http://www.hmdb.ca/metabolites/HMDB06344) |
| cyclo(gly-pro) | Peptide | Dipeptide |  |
| glycylleucine | Peptide | Dipeptide | [HMDB00759](http://www.hmdb.ca/metabolites/HMDB00759) |
| glycylvaline | Peptide | Dipeptide | [HMDB28854](http://www.hmdb.ca/metabolites/HMDB28854) |
| histidylalanine | Peptide | Dipeptide | [HMDB28878](http://www.hmdb.ca/metabolites/HMDB28878) |
| isoleucylglycine | Peptide | Dipeptide | [HMDB28907](http://www.hmdb.ca/metabolites/HMDB28907) |
| leucylglutamine* | Peptide | Dipeptide | [HMDB28927](http://www.hmdb.ca/metabolites/HMDB28927) |
| leucylglycine | Peptide | Dipeptide | [HMDB28929](http://www.hmdb.ca/metabolites/HMDB28929) |
| lysylleucine | Peptide | Dipeptide | [HMDB28955](http://www.hmdb.ca/metabolites/HMDB28955) |
| phenylalanylalanine | Peptide | Dipeptide |  |
| phenylalanylglycine | Peptide | Dipeptide | [HMDB28995](http://www.hmdb.ca/metabolites/HMDB28995) |
| phenylalanylhydroxyproline* | Peptide | Dipeptide | [HMDB28996,HMDB11176](http://www.hmdb.ca/metabolites/HMDB28996,HMDB11176) |
| prolylglycine | Peptide | Dipeptide | [HMDB11178](http://www.hmdb.ca/metabolites/HMDB11178) |
| threonylphenylalanine | Peptide | Dipeptide | [HMDB29068](http://www.hmdb.ca/metabolites/HMDB29068) |
| tryptophylglycine | Peptide | Dipeptide | [HMDB29083](http://www.hmdb.ca/metabolites/HMDB29083) |
| tyrosylglycine | Peptide | Dipeptide | [HMDB29105](http://www.hmdb.ca/metabolites/HMDB29105) |
| valylglutamine | Peptide | Dipeptide | [HMDB29125](http://www.hmdb.ca/metabolites/HMDB29125) |
| valylglycine | Peptide | Dipeptide | [HMDB29127](http://www.hmdb.ca/metabolites/HMDB29127) |
| valylleucine | Peptide | Dipeptide | [HMDB29131](http://www.hmdb.ca/metabolites/HMDB29131) |
| ADpSGEGDFXAEGGGVR* | Peptide | Fibrinogen Cleavage Peptide |  |
| ADSGEGDFXAEGGGVR* | Peptide | Fibrinogen Cleavage Peptide |  |
| DSGEGDFXAEGGGVR* | Peptide | Fibrinogen Cleavage Peptide |  |
| Fibrinopeptide A (3-16) | Peptide | Fibrinogen Cleavage Peptide |  |
| Fibrinopeptide B | Peptide | Fibrinogen Cleavage Peptide |  |
| Fibrinopeptide B (1-11) | Peptide | Fibrinogen Cleavage Peptide |  |
| Fibrinopeptide B (1-13) | Peptide | Fibrinogen Cleavage Peptide |  |
| gamma-glutamyl-alpha-lysine | Peptide | Gamma-glutamyl Amino Acid |  |
| gamma-glutamylcitrulline* | Peptide | Gamma-glutamyl Amino Acid |  |
| gamma-glutamylglutamate | Peptide | Gamma-glutamyl Amino Acid | [HMDB11737](http://www.hmdb.ca/metabolites/HMDB11737) |
| gamma-glutamylglutamine | Peptide | Gamma-glutamyl Amino Acid | [HMDB11738](http://www.hmdb.ca/metabolites/HMDB11738) |
| gamma-glutamylglycine | Peptide | Gamma-glutamyl Amino Acid | [HMDB11667](http://www.hmdb.ca/metabolites/HMDB11667) |
| gamma-glutamylhistidine | Peptide | Gamma-glutamyl Amino Acid |  |
| gamma-glutamylisoleucine* | Peptide | Gamma-glutamyl Amino Acid | [HMDB11170](http://www.hmdb.ca/metabolites/HMDB11170) |
| gamma-glutamylleucine | Peptide | Gamma-glutamyl Amino Acid | [HMDB11171](http://www.hmdb.ca/metabolites/HMDB11171) |
| gamma-glutamylmethionine | Peptide | Gamma-glutamyl Amino Acid | [HMDB29155](http://www.hmdb.ca/metabolites/HMDB29155) |
| gamma-glutamylphenylalanine | Peptide | Gamma-glutamyl Amino Acid | [HMDB00594](http://www.hmdb.ca/metabolites/HMDB00594) |
| gamma-glutamylthreonine | Peptide | Gamma-glutamyl Amino Acid | [HMDB29159](http://www.hmdb.ca/metabolites/HMDB29159) |
| gamma-glutamyltyrosine | Peptide | Gamma-glutamyl Amino Acid | [HMDB11741](http://www.hmdb.ca/metabolites/HMDB11741) |
| gamma-glutamylvaline | Peptide | Gamma-glutamyl Amino Acid | [HMDB11172](http://www.hmdb.ca/metabolites/HMDB11172) |
| bradykinin | Peptide | Polypeptide | [HMDB04246](http://www.hmdb.ca/metabolites/HMDB04246) |
| bradykinin, des-arg(9) | Peptide | Polypeptide | [HMDB04246](http://www.hmdb.ca/metabolites/HMDB04246) |
| HWESASLLR | Peptide | Polypeptide |  |
| HWESASXX* | Peptide | Polypeptide |  |
| HXGXA* | Peptide | Polypeptide |  |
| XHWESASXXR* | Peptide | Polypeptide |  |

HMDB, Human Metabolome Database

*Indicates compounds that have not been officially confirmed based on a standard, but identified by virtue of their recurrent chromatographic and spectral nature

**Additional file 1: Table SⅠI. Association between plasma metabolites and CAC in AAs**

| **Biochemical** | **Super-pathway** | **Sub-pathway** | **β-Estimate** | **SE** | **P_FDR_** |
| --- | --- | --- | --- | --- | --- |
| androstenediol (3alpha, 17alpha) monosulfate (3) | Lipid | Androgenic Steroids | -0.52 | 0.13 | 0.007 |
| dehydroepiandrosterone sulfate (DHEA-S) | Lipid | Androgenic Steroids | -0.45 | 0.12 | 0.018 |
| androstenediol (3beta,17beta) monosulfate (1) | Lipid | Androgenic Steroids | -0.45 | 0.12 | 0.018 |
| androsterone sulfate | Lipid | Androgenic Steroids | -0.43 | 0.12 | 0.03 |
| epiandrosterone sulfate | Lipid | Androgenic Steroids | -0.42 | 0.12 | 0.034 |
| androstenediol (3beta,17beta) disulfate (1) | Lipid | Androgenic Steroids | -0.43 | 0.13 | 0.036 |
| decanoylcarnitine (C10) | Lipid | Fatty Acid Metabolism (Acyl Carnitine, Medium Chain) | -0.65 | 0.14 | 0.0026 |
| nonanoylcarnitine (C9) | Lipid | Fatty Acid Metabolism (Acyl Carnitine, Medium Chain) | -0.71 | 0.16 | 0.0026 |
| hexanoylcarnitine (C6) | Lipid | Fatty Acid Metabolism (Acyl Carnitine, Medium Chain) | -0.59 | 0.14 | 0.0035 |
| octanoylcarnitine (C8) | Lipid | Fatty Acid Metabolism (Acyl Carnitine, Medium Chain) | -0.56 | 0.13 | 0.0038 |
| laurylcarnitine (C12) | Lipid | Fatty Acid Metabolism (Acyl Carnitine, Medium Chain) | -0.43 | 0.12 | 0.018 |
| 5-dodecenoylcarnitine (C12:1) | Lipid | Fatty Acid Metabolism (Acyl Carnitine, Monounsaturated) | -0.44 | 0.13 | 0.022 |
| 3-hydroxymyristate | Lipid | Fatty Acid, Monohydroxy | -0.42 | 0.12 | 0.018 |
| 1-palmitoyl-2-docosahexaenoyl-GPC (16:0/22:6) | Lipid | Phosphatidylcholine (PC) | -0.57 | 0.15 | 0.016 |
| pregnenetriol sulfate* | Lipid | Pregnenolone Steroids | -0.43 | 0.13 | 0.046 |
| glyco-beta-muricholate | Lipid | Primary Bile Acid Metabolism | 0.51 | 0.11 | 0.0026 |
| hyocholate | Lipid | Secondary Bile Acid Metabolism | 0.45 | 0.13 | 0.031 |
| tryptophylglycine | Peptide | Dipeptide | 1.99 | 0.62 | 0.049^†^ |
| gamma-glutamylleucine | Peptide | Gamma-glutamyl Amino Acid | -0.64 | 0.15 | 0.0037 |
| gamma-glutamyl-alpha-lysine | Peptide | Gamma-glutamyl Amino Acid | -0.64 | 0.16 | 0.0093 |
| gamma-glutamylmethionine | Peptide | Gamma-glutamyl Amino Acid | -0.61 | 0.17 | 0.018 |
| gamma-glutamylglycine | Peptide | Gamma-glutamyl Amino Acid | -0.6 | 0.17 | 0.025 |

AA, African American; BMI, Body mass index; CAC, Coronary artery calcium; SE, Standard error

Model adjusted for age, sex, BMI, smoking status, hypertension status, CVD, duration of diabetes, date of plasma collection, time between plasma collection and CT exam

*Indicates compounds that have not been officially confirmed based on a standard, but identified by virtue of their recurrent chromatographic and spectral nature

^†^Indicates results derived from dichotomized metabolite values, i.e. presence vs absence, when >50% of the data were missing

**Additional file 1: Table SⅡI. Association between plasma metabolites and CAC in AAs (Additionally adjusted for LDL-C)**

| **Biochemical** | **Super-pathway** | **Sub-pathway** | **β-Estimate** | **SE** | **P_FDR_** |
| --- | --- | --- | --- | --- | --- |
| 3-hydroxy-2-ethylpropionate | Amino Acid | Leucine, Isoleucine and Valine Metabolism | -0.42 | 0.13 | 0.027 |
| androstenediol (3alpha, 17alpha) monosulfate (3) | Lipid | Androgenic Steroids | -0.56 | 0.14 | 0.0038 |
| dehydroepiandrosterone sulfate (DHEA-S) | Lipid | Androgenic Steroids | -0.49 | 0.13 | 0.0072 |
| androstenediol (3beta,17beta) monosulfate (1) | Lipid | Androgenic Steroids | -0.48 | 0.13 | 0.0091 |
| androstenediol (3beta,17beta) disulfate (1) | Lipid | Androgenic Steroids | -0.46 | 0.13 | 0.016 |
| androsterone sulfate | Lipid | Androgenic Steroids | -0.45 | 0.13 | 0.019 |
| epiandrosterone sulfate | Lipid | Androgenic Steroids | -0.43 | 0.12 | 0.023 |
| 5alpha-androstan-3alpha,17beta-diol monosulfate (1) | Lipid | Androgenic Steroids | -0.41 | 0.13 | 0.046 |
| androstenediol (3beta,17beta) monosulfate (2) | Lipid | Androgenic Steroids | -0.37 | 0.12 | 0.048 |
| 3-hydroxydecanoylcarnitine | Lipid | Fatty Acid Metabolism (Acyl Carnitine, Hydroxy) | -0.41 | 0.13 | 0.039 |
| myristoylcarnitine (C14) | Lipid | Fatty Acid Metabolism (Acyl Carnitine, Long Chain Saturated) | -0.51 | 0.15 | 0.022 |
| palmitoylcarnitine (C16) | Lipid | Fatty Acid Metabolism (Acyl Carnitine, Long Chain Saturated) | -0.44 | 0.14 | 0.048 |
| octanoylcarnitine (C8) | Lipid | Fatty Acid Metabolism (Acyl Carnitine, Medium Chain) | -0.63 | 0.13 | 0.0011 |
| decanoylcarnitine (C10) | Lipid | Fatty Acid Metabolism (Acyl Carnitine, Medium Chain) | -0.69 | 0.14 | 0.0011 |
| hexanoylcarnitine (C6) | Lipid | Fatty Acid Metabolism (Acyl Carnitine, Medium Chain) | -0.61 | 0.14 | 0.0011 |
| laurylcarnitine (C12) | Lipid | Fatty Acid Metabolism (Acyl Carnitine, Medium Chain) | -0.50 | 0.11 | 0.0022 |
| nonanoylcarnitine (C9) | Lipid | Fatty Acid Metabolism (Acyl Carnitine, Medium Chain) | -0.70 | 0.16 | 0.0026 |
| 5-dodecenoylcarnitine (C12:1) | Lipid | Fatty Acid Metabolism (Acyl Carnitine, Monounsaturated) | -0.47 | 0.12 | 0.011 |
| myristoleoylcarnitine (C14:1)* | Lipid | Fatty Acid Metabolism (Acyl Carnitine, Monounsaturated) | -0.40 | 0.12 | 0.023 |
| cis-4-decenoylcarnitine (C10:1) | Lipid | Fatty Acid Metabolism (Acyl Carnitine, Monounsaturated) | -0.41 | 0.12 | 0.027 |
| dodecadienoate (12:2)* | Lipid | Fatty Acid, Dicarboxylate | -0.47 | 0.13 | 0.016 |
| 3-hydroxymyristate | Lipid | Fatty Acid, Monohydroxy | -0.49 | 0.12 | 0.0030 |
| 3-hydroxylaurate | Lipid | Fatty Acid, Monohydroxy | -0.49 | 0.12 | 0.0044 |
| 3-hydroxydecanoate | Lipid | Fatty Acid, Monohydroxy | -0.40 | 0.12 | 0.033 |
| 5-dodecenoate (12:1n7) | Lipid | Medium Chain Fatty Acid | -0.41 | 0.12 | 0.027 |
| 1-palmitoyl-2-docosahexaenoyl-GPC (16:0/22:6) | Lipid | Phosphatidylcholine (PC) | -0.69 | 0.15 | 0.0011 |
| 1-palmitoyl-2-dihomo-linolenoyl-GPC (16:0/20:3n3 or 6)* | Lipid | Phosphatidylcholine (PC) | -0.41 | 0.13 | 0.047 |
| 1-myristoyl-2-arachidonoyl-GPC (14:0/20:4)* | Lipid | Phosphatidylcholine (PC) | -0.55 | 0.18 | 0.048 |
| pregnenetriol sulfate* | Lipid | Pregnenolone Steroids | -0.46 | 0.13 | 0.024 |
| pregnenediol sulfate (C21H34O5S)* | Lipid | Pregnenolone Steroids | -0.45 | 0.15 | 0.048 |
| glyco-beta-muricholate | Lipid | Primary Bile Acid Metabolism | 0.52 | 0.12 | 0.0011 |
| hyocholate | Lipid | Secondary Bile Acid Metabolism | 0.44 | 0.13 | 0.027 |
| gamma-glutamylleucine | Peptide | Gamma-glutamyl Amino Acid | -0.58 | 0.15 | 0.0072 |
| gamma-glutamyl-alpha-lysine | Peptide | Gamma-glutamyl Amino Acid | -0.59 | 0.17 | 0.020 |
| gamma-glutamylmethionine | Peptide | Gamma-glutamyl Amino Acid | -0.55 | 0.17 | 0.035 |
| gamma-glutamylvaline | Peptide | Gamma-glutamyl Amino Acid | -0.37 | 0.12 | 0.043 |

AA, African American; BMI, Body mass index; CAC, Coronary artery calcium; LDL-C, Low-density lipoprotein cholesterol; SE, Standard error

Model adjusted for age, sex, BMI, smoking status, hypertension status, CVD, duration of diabetes, date of plasma collection, time between plasma collection and CT exam, LDL-C

*Indicates compounds that have not been officially confirmed based on a standard, but identified by virtue of their recurrent chromatographic and spectral nature

**Additional file 1: Table SIV. Association between plasma metabolites and CAC in AAs (Additionally adjusted for LDL-C and Statin)**

| **Biochemical** | **Super-pathway** | **Sub-pathway** | **β-Estimate** | **SE** | **P_FDR_** |
| --- | --- | --- | --- | --- | --- |
| 3-hydroxy-2-ethylpropionate | Amino Acid | Leucine, Isoleucine and Valine Metabolism | -0.41 | 0.12 | 0.033 |
| androstenediol (3alpha, 17alpha) monosulfate (3) | Lipid | Androgenic Steroids | -0.52 | 0.13 | 0.0094 |
| dehydroepiandrosterone sulfate (DHEA-S) | Lipid | Androgenic Steroids | -0.45 | 0.13 | 0.018 |
| androstenediol (3beta,17beta) monosulfate (1) | Lipid | Androgenic Steroids | -0.45 | 0.13 | 0.021 |
| androsterone sulfate | Lipid | Androgenic Steroids | -0.43 | 0.12 | 0.026 |
| epiandrosterone sulfate | Lipid | Androgenic Steroids | -0.42 | 0.12 | 0.026 |
| androstenediol (3beta,17beta) disulfate (1) | Lipid | Androgenic Steroids | -0.42 | 0.13 | 0.038 |
| 5alpha-androstan-3alpha,17beta-diol monosulfate (1) | Lipid | Androgenic Steroids | -0.40 | 0.13 | 0.047 |
| myristoylcarnitine (C14) | Lipid | Fatty Acid Metabolism (Acyl Carnitine, Long Chain Saturated) | -0.51 | 0.15 | 0.026 |
| decanoylcarnitine (C10) | Lipid | Fatty Acid Metabolism (Acyl Carnitine, Medium Chain) | -0.68 | 0.14 | 0.0008 |
| octanoylcarnitine (C8) | Lipid | Fatty Acid Metabolism (Acyl Carnitine, Medium Chain) | -0.61 | 0.13 | 0.0009 |
| hexanoylcarnitine (C6) | Lipid | Fatty Acid Metabolism (Acyl Carnitine, Medium Chain) | -0.59 | 0.14 | 0.0024 |
| laurylcarnitine (C12) | Lipid | Fatty Acid Metabolism (Acyl Carnitine, Medium Chain) | -0.48 | 0.12 | 0.0039 |
| nonanoylcarnitine (C9) | Lipid | Fatty Acid Metabolism (Acyl Carnitine, Medium Chain) | -0.68 | 0.16 | 0.0039 |
| 5-dodecenoylcarnitine (C12:1) | Lipid | Fatty Acid Metabolism (Acyl Carnitine, Monounsaturated) | -0.46 | 0.13 | 0.016 |
| cis-4-decenoylcarnitine (C10:1) | Lipid | Fatty Acid Metabolism (Acyl Carnitine, Monounsaturated) | -0.41 | 0.12 | 0.029 |
| myristoleoylcarnitine (C14:1)* | Lipid | Fatty Acid Metabolism (Acyl Carnitine, Monounsaturated) | -0.40 | 0.12 | 0.029 |
| dodecadienoate (12:2)* | Lipid | Fatty Acid, Dicarboxylate | -0.47 | 0.13 | 0.018 |
| 3-hydroxymyristate | Lipid | Fatty Acid, Monohydroxy | -0.46 | 0.12 | 0.0094 |
| 3-hydroxylaurate | Lipid | Fatty Acid, Monohydroxy | -0.45 | 0.12 | 0.016 |
| 5-dodecenoate (12:1n7) | Lipid | Medium Chain Fatty Acid | -0.38 | 0.12 | 0.047 |
| 1-palmitoyl-2-docosahexaenoyl-GPC (16:0/22:6) | Lipid | Phosphatidylcholine (PC) | -0.69 | 0.15 | 0.0008 |
| 1-oleoyl-2-docosahexaenoyl-GPC (18:1/22:6)* | Lipid | Phosphatidylcholine (PC) | -0.38 | 0.12 | 0.047 |
| 1-myristoyl-2-arachidonoyl-GPC (14:0/20:4)* | Lipid | Phosphatidylcholine (PC) | -0.55 | 0.18 | 0.047 |
| pregnenetriol sulfate* | Lipid | Pregnenolone Steroids | -0.41 | 0.13 | 0.050 |
| glyco-beta-muricholate | Lipid | Primary Bile Acid Metabolism | 0.54 | 0.11 | 0.0008 |
| tauro-beta-muricholate | Lipid | Primary Bile Acid Metabolism | 0.40 | 0.13 | 0.047 |
| hyocholate | Lipid | Secondary Bile Acid Metabolism | 0.45 | 0.13 | 0.022 |
| glycohyocholate | Lipid | Secondary Bile Acid Metabolism | 0.44 | 0.14 | 0.038 |
| gamma-glutamylleucine | Peptide | Gamma-glutamyl Amino Acid | -0.57 | 0.15 | 0.0098 |
| gamma-glutamyl-alpha-lysine | Peptide | Gamma-glutamyl Amino Acid | -0.59 | 0.17 | 0.022 |
| gamma-glutamylvaline | Peptide | Gamma-glutamyl Amino Acid | -0.37 | 0.12 | 0.047 |
| gamma-glutamylmethionine | Peptide | Gamma-glutamyl Amino Acid | -0.53 | 0.17 | 0.047 |

AA, African American; BMI, Body mass index; CAC, Coronary artery calcium; LDL-C, Low-density lipoprotein cholesterol; SE, Standard error

Model adjusted for age, sex, BMI, smoking status, hypertension status, CVD, duration of diabetes, date of plasma collection, time between plasma collection and CT exam, LDL-C, statin use

*Indicates compounds that have not been officially confirmed based on a standard, but identified by virtue of their recurrent chromatographic and spectral nature

**Additional file 1: Table SV. Association between plasma metabolites and CAC in EAs**

| **Biochemical** | **Super-pathway** | **Sub-pathway** | **β-Estimate** | **SE** | **P_FDR_** |
| --- | --- | --- | --- | --- | --- |
| 3-methylglutaconate | Amino Acid | Leucine, Isoleucine and Valine Metabolism | 0.57 | 0.15 | 0.029 |
| N-acetylleucine | Amino Acid | Leucine, Isoleucine and Valine Metabolism | 0.56 | 0.15 | 0.030 |
| N6-acetyllysine | Amino Acid | Lysine Metabolism | 0.55 | 0.14 | 0.023 |
| N-acetylphenylalanine | Amino Acid | Phenylalanine Metabolism | 0.51 | 0.15 | 0.043 |
| erythronate* | Carbohydrate | Aminosugar Metabolism | 0.60 | 0.15 | 0.023 |
| dehydroepiandrosterone sulfate (DHEA-S) | Lipid | Androgenic Steroids | -0.54 | 0.16 | 0.043 |
| androstenediol (3beta,17beta) monosulfate (2) | Lipid | Androgenic Steroids | -0.54 | 0.16 | 0.043 |
| 3-hydroxy-3-methylglutarate | Lipid | Mevalonate Metabolism | 0.80 | 0.15 | 6.44E-05 |
| pregnen-diol disulfate* | Lipid | Pregnenolone Steroids | -0.58 | 0.16 | 0.036 |
| 5alpha-pregnan-3beta,20alpha-diol disulfate | Lipid | Progestin Steroids | -0.47 | 0.13 | 0.035 |
| 5alpha-pregnan-diol disulfate | Lipid | Progestin Steroids | -1.02 | 0.28 | 0.035^†^ |
| campesterol | Lipid | Sterol | 1.07 | 0.31 | 0.043^†^ |

BMI, Body mass index; CAC, Coronary artery calcium; EA, European American; SE, Standard error; TCA, tricarboxylic acid cycle

Model adjusted for age, sex, BMI, smoking status, hypertension status, CVD, duration of diabetes, date of plasma collection, time between plasma collection and CT exam

*Indicates compounds that have not been officially confirmed based on a standard, but identified by virtue of their recurrent chromatographic and spectral nature

^†^Indicates results derived from dichotomized metabolite values, i.e. presence vs absence, when >50% of the data were missing

**Additional file 1: Table SⅤI. Association between plasma metabolites and CAC in EAs (Additionally adjusted for LDL-C)**

| **Biochemical** | **Super-pathway** | **Sub-pathway** | **β-Estimate** | **SE** | **P_FDR_** |
| --- | --- | --- | --- | --- | --- |
| N-acetylserine | Amino Acid | Glycine, Serine and Threonine Metabolism | 0.51 | 0.15 | 0.023 |
| 1-methyl-4-imidazoleacetate | Amino Acid | Histidine Metabolism | 0.42 | 0.14 | 0.043 |
| 3-methylglutaconate | Amino Acid | Leucine, Isoleucine and Valine Metabolism | 0.63 | 0.16 | 0.013 |
| N-acetylleucine | Amino Acid | Leucine, Isoleucine and Valine Metabolism | 0.60 | 0.17 | 0.020 |
| isobutyrylcarnitine (C4) | Amino Acid | Leucine, Isoleucine and Valine Metabolism | 0.49 | 0.15 | 0.024 |
| ethylmalonate | Amino Acid | Leucine, Isoleucine and Valine Metabolism | 0.42 | 0.14 | 0.043 |
| N6-acetyllysine | Amino Acid | Lysine Metabolism | 0.54 | 0.14 | 0.017 |
| 5-hydroxylysine | Amino Acid | Lysine Metabolism | 0.54 | 0.17 | 0.028 |
| methionine sulfoxide | Amino Acid | Methionine, Cysteine, SAM and Taurine Metabolism | 0.65 | 0.18 | 0.019 |
| 2,3-dihydroxy-5-methylthio-4-pentenoate (DMTPA)* | Amino Acid | Methionine, Cysteine, SAM and Taurine Metabolism | 0.55 | 0.15 | 0.019 |
| lanthionine | Amino Acid | Methionine, Cysteine, SAM and Taurine Metabolism | 0.50 | 0.16 | 0.039 |
| N-acetylphenylalanine | Amino Acid | Phenylalanine Metabolism | 0.62 | 0.15 | 0.013 |
| N-acetyl-isoputreanine | Amino Acid | Polyamine Metabolism | 0.50 | 0.14 | 0.020 |
| 4-acetamidobutanoate | Amino Acid | Polyamine Metabolism | 0.45 | 0.14 | 0.035 |
| picolinate | Amino Acid | Tryptophan Metabolism | 0.43 | 0.14 | 0.039 |
| glucuronate | Carbohydrate | Aminosugar Metabolism | 0.55 | 0.16 | 0.023 |
| erythronate* | Carbohydrate | Aminosugar Metabolism | 0.55 | 0.16 | 0.023 |
| arabinose | Carbohydrate | Pentose Metabolism | 0.46 | 0.15 | 0.045 |
| bilirubin (E,E)* | Cofactors and Vitamins | Hemoglobin and Porphyrin Metabolism | -0.57 | 0.17 | 0.023 |
| quinolinate | Cofactors and Vitamins | Nicotinate and Nicotinamide Metabolism | 0.45 | 0.15 | 0.039 |
| citraconate/glutaconate | Energy | TCA Cycle | 0.50 | 0.16 | 0.039 |
| androstenediol (3beta,17beta) monosulfate (2) | Lipid | Androgenic Steroids | -0.58 | 0.16 | 0.020 |
| dehydroepiandrosterone sulfate (DHEA-S) | Lipid | Androgenic Steroids | -0.54 | 0.16 | 0.023 |
| 16a-hydroxy DHEA 3-sulfate | Lipid | Androgenic Steroids | -0.49 | 0.15 | 0.023 |
| 5alpha-androstan-3beta,17beta-diol disulfate | Lipid | Androgenic Steroids | -0.57 | 0.17 | 0.024 |
| androsterone sulfate | Lipid | Androgenic Steroids | -0.47 | 0.15 | 0.031 |
| androstenediol (3alpha, 17alpha) monosulfate (3) | Lipid | Androgenic Steroids | -0.49 | 0.15 | 0.033 |
| androstenediol (3beta,17beta) disulfate (1) | Lipid | Androgenic Steroids | -0.52 | 0.17 | 0.039 |
| N-palmitoyl-sphinganine (d18:0/16:0) | Lipid | Dihydroceramides | 0.55 | 0.16 | 0.023 |
| palmitoyl dihydrosphingomyelin (d18:0/16:0)* | Lipid | Dihydrosphingomyelins | 0.55 | 0.17 | 0.027 |
| leukotriene B4 | Lipid | Eicosanoid | 0.63 | 0.18 | 0.023 |
| 5-HETE | Lipid | Eicosanoid | 0.56 | 0.18 | 0.042 |
| oleoyl ethanolamide | Lipid | Endocannabinoid | 0.48 | 0.16 | 0.042 |
| picolinoylglycine | Lipid | Fatty Acid Metabolism (Acyl Glycine) | 0.47 | 0.14 | 0.023 |
| N-acetyl-2-aminooctanoate* | Lipid | Fatty Acid, Amino | 0.54 | 0.15 | 0.020 |
| 3,4-dihydroxybutyrate | Lipid | Fatty Acid, Dihydroxy | 0.48 | 0.16 | 0.039 |
| glycerol | Lipid | Glycerolipid Metabolism | 0.50 | 0.16 | 0.042 |
| docosatrienoate (22:3n6)* | Lipid | Long Chain Polyunsaturated Fatty Acid (n3 and n6) | 0.55 | 0.15 | 0.023 |
| 1-(1-enyl-oleoyl)-GPE (P-18:1)* | Lipid | Lysoplasmalogen | 0.61 | 0.18 | 0.023 |
| 1-(1-enyl-palmitoyl)-GPE (P-16:0)* | Lipid | Lysoplasmalogen | 0.62 | 0.19 | 0.028 |
| 10-undecenoate (11:1n1) | Lipid | Medium Chain Fatty Acid | -0.48 | 0.16 | 0.039 |
| 3-hydroxy-3-methylglutarate | Lipid | Mevalonate Metabolism | 0.93 | 0.16 | 4.64E-06 |
| 1-linolenoylglycerol (18:3) | Lipid | Monoacylglycerol | -0.47 | 0.16 | 0.042 |
| glycerophosphoinositol* | Lipid | Phospholipid Metabolism | 0.54 | 0.16 | 0.024 |
| pregnen-diol disulfate* | Lipid | Pregnenolone Steroids | -0.63 | 0.16 | 0.013 |
| pregnenolone sulfate | Lipid | Pregnenolone Steroids | -0.55 | 0.16 | 0.023 |
| pregnenetriol disulfate* | Lipid | Pregnenolone Steroids | -0.52 | 0.16 | 0.025 |
| pregnenetriol sulfate* | Lipid | Pregnenolone Steroids | -0.55 | 0.17 | 0.027 |
| 5alpha-pregnan-3beta,20alpha-diol disulfate | Lipid | Progestin Steroids | -0.52 | 0.13 | 0.013 |
| 5alpha-pregnan-diol disulfate | Lipid | Progestin Steroids | -1.06 | 0.29 | 0.019^†^ |
| campesterol | Lipid | Sterol | 1.14 | 0.33 | 0.023^†^ |
| N6-carbamoylthreonyladenosine | Nucleotide | Purine Metabolism, Adenine containing | 0.45 | 0.14 | 0.036 |
| N2,N2-dimethylguanosine | Nucleotide | Purine Metabolism, Guanine containing | 0.59 | 0.15 | 0.013 |
| 7-methylguanine | Nucleotide | Purine Metabolism, Guanine containing | 0.61 | 0.17 | 0.023 |
| 2'-O-methyluridine | Nucleotide | Pyrimidine Metabolism, Uracil containing | 0.53 | 0.16 | 0.028 |
| phenylacetylglutamate | Peptide | Acetylated Peptides | 0.48 | 0.16 | 0.047 |
| bradykinin | Peptide | Polypeptide | 0.50 | 0.16 | 0.039 |

BMI, Body mass index; CAC, Coronary artery calcium; EA, European American; LDL-C, Low-density lipoprotein cholesterol; SE, Standard error; TCA, tricarboxylic acid cycle

Model adjusted for age, sex, BMI, smoking status, hypertension status, CVD, duration of diabetes, date of plasma collection, time between plasma collection and CT exam, LDL-C

*Indicates compounds that have not been officially confirmed based on a standard, but identified by virtue of their recurrent chromatographic and spectral nature

^†^Indicates results derived from dichotomized metabolite values, i.e. presence vs absence, when >50% of the data were missing

**Additional file 1: Table SⅦ. Association between plasma metabolites and CAC in EAs (Additionally adjusted for LDL-C and Statin)**

| **Biochemical** | **Super-pathway** | **Sub-pathway** | **β-Estimate** | **SE** | **P_FDR_** |
| --- | --- | --- | --- | --- | --- |
| 1-methyl-4-imidazoleacetate | Amino Acid | Histidine Metabolism | 0.43 | 0.14 | 0.049 |
| N-acetylleucine | Amino Acid | Leucine, Isoleucine and Valine Metabolism | 0.50 | 0.16 | 0.048 |
| isobutyrylcarnitine (C4) | Amino Acid | Leucine, Isoleucine and Valine Metabolism | 0.44 | 0.14 | 0.049 |
| N6-acetyllysine | Amino Acid | Lysine Metabolism | 0.45 | 0.14 | 0.045 |
| 5-hydroxylysine | Amino Acid | Lysine Metabolism | 0.51 | 0.16 | 0.048 |
| 2,3-dihydroxy-5-methylthio-4-pentenoate (DMTPA)* | Amino Acid | Methionine, Cysteine, SAM and Taurine Metabolism | 0.50 | 0.14 | 0.039 |
| methionine sulfoxide | Amino Acid | Methionine, Cysteine, SAM and Taurine Metabolism | 0.54 | 0.17 | 0.047 |
| N-acetylphenylalanine | Amino Acid | Phenylalanine Metabolism | 0.58 | 0.14 | 0.0088 |
| N-acetyl-isoputreanine | Amino Acid | Polyamine Metabolism | 0.46 | 0.14 | 0.045 |
| picolinate | Amino Acid | Tryptophan Metabolism | 0.42 | 0.12 | 0.040 |
| glucuronate | Carbohydrate | Aminosugar Metabolism | 0.60 | 0.15 | 0.012 |
| erythronate* | Carbohydrate | Aminosugar Metabolism | 0.50 | 0.16 | 0.048 |
| arabinose | Carbohydrate | Pentose Metabolism | 0.48 | 0.15 | 0.049 |
| ascorbic acid 2-sulfate | Cofactors and Vitamins | Ascorbate and Aldarate Metabolism | 0.50 | 0.16 | 0.049 |
| androstenediol (3beta,17beta) monosulfate (2) | Lipid | Androgenic Steroids | -0.52 | 0.16 | 0.047 |
| N-palmitoyl-sphinganine (d18:0/16:0) | Lipid | Dihydroceramides | 0.56 | 0.16 | 0.039 |
| palmitoyl dihydrosphingomyelin (d18:0/16:0)* | Lipid | Dihydrosphingomyelins | 0.68 | 0.16 | 0.0068 |
| picolinoylglycine | Lipid | Fatty Acid Metabolism (Acyl Glycine) | 0.44 | 0.12 | 0.039 |
| N-acetyl-2-aminooctanoate* | Lipid | Fatty Acid, Amino | 0.49 | 0.14 | 0.040 |
| 3,4-dihydroxybutyrate | Lipid | Fatty Acid, Dihydroxy | 0.48 | 0.15 | 0.047 |
| 13-HODE + 9-HODE | Lipid | Fatty Acid, Monohydroxy | 0.56 | 0.17 | 0.047 |
| 1-(1-enyl-oleoyl)-GPE (P-18:1)* | Lipid | Lysoplasmalogen | 0.58 | 0.16 | 0.039 |
| 1-(1-enyl-palmitoyl)-GPE (P-16:0)* | Lipid | Lysoplasmalogen | 0.57 | 0.18 | 0.047 |
| 10-undecenoate (11:1n1) | Lipid | Medium Chain Fatty Acid | -0.48 | 0.15 | 0.048 |
| 3-hydroxy-3-methylglutarate | Lipid | Mevalonate Metabolism | 0.80 | 0.16 | 3.20E-04 |
| 1-linolenoylglycerol (18:3) | Lipid | Monoacylglycerol | -0.62 | 0.15 | 0.0088 |
| glycerophosphoinositol* | Lipid | Phospholipid Metabolism | 0.57 | 0.15 | 0.026 |
| 1-(1-enyl-palmitoyl)-2-palmitoleoyl-GPC (P-16:0/16:1)* | Lipid | Plasmalogen | 0.51 | 0.16 | 0.049 |
| pregnen-diol disulfate* | Lipid | Pregnenolone Steroids | -0.50 | 0.16 | 0.047 |
| pregnenolone sulfate | Lipid | Pregnenolone Steroids | -0.48 | 0.15 | 0.050 |
| 5alpha-pregnan-3beta,20alpha-diol disulfate | Lipid | Progestin Steroids | -0.44 | 0.13 | 0.040 |
| 5alpha-pregnan-diol disulfate | Lipid | Progestin Steroids | -0.90 | 0.28 | 0.047^†^ |
| campesterol | Lipid | Sterol | 0.97 | 0.30 | 0.047^†^ |
| N2,N2-dimethylguanosine | Nucleotide | Purine Metabolism, Guanine containing | 0.55 | 0.14 | 0.012 |
| 7-methylguanine | Nucleotide | Purine Metabolism, Guanine containing | 0.53 | 0.16 | 0.045 |
| 2'-O-methyluridine | Nucleotide | Pyrimidine Metabolism, Uracil containing | 0.48 | 0.15 | 0.048 |

BMI, Body mass index; CAC, Coronary artery calcium; EA, European American; LDL-C, Low-density lipoprotein cholesterol; SE,

Standard error; TCA, tricarboxylic acid cycle

Model adjusted for age, sex, BMI, smoking status, hypertension status, CVD, duration of diabetes, date of plasma collection, time between plasma collection and CT exam, LDL-C, statin use

*Indicates compounds that have not been officially confirmed based on a standard, but identified by virtue of their recurrent chromatographic and spectral nature

^†^Indicates results derived from dichotomized metabolite values, i.e. presence vs absence, when >50% of the data were missing

**Additional file 1: Table SVIII. Association between plasma metabolites and CAC in AAs (Additionally adjusted for HbA1C)**

| **Biochemical** | **Super-pathway** | **Sub-pathway** | **β-Estimate** | **SE** | **P_FDR_** |
| --- | --- | --- | --- | --- | --- |
| ornithine | Amino Acid | Urea cycle; Arginine and Proline Metabolism | 0.38 | 0.12 | 0.046 |
| androstenediol (3alpha, 17alpha) monosulfate (3) | Lipid | Androgenic Steroids | -0.50 | 0.13 | 0.020 |
| dehydroepiandrosterone sulfate (DHEA-S) | Lipid | Androgenic Steroids | -0.43 | 0.12 | 0.029 |
| androstenediol (3beta,17beta) monosulfate (1) | Lipid | Androgenic Steroids | -0.44 | 0.12 | 0.029 |
| androsterone sulfate | Lipid | Androgenic Steroids | -0.42 | 0.12 | 0.033 |
| androstenediol (3beta,17beta) disulfate (1) | Lipid | Androgenic Steroids | -0.43 | 0.13 | 0.035 |
| epiandrosterone sulfate | Lipid | Androgenic Steroids | -0.41 | 0.12 | 0.036 |
| 5alpha-androstan-3alpha,17beta-diol monosulfate (1) | Lipid | Androgenic Steroids | -0.40 | 0.13 | 0.050 |
| myristoylcarnitine (C14) | Lipid | Fatty Acid Metabolism (Acyl Carnitine, Long Chain Saturated) | -0.49 | 0.15 | 0.038 |
| decanoylcarnitine (C10) | Lipid | Fatty Acid Metabolism (Acyl Carnitine, Medium Chain) | -0.64 | 0.14 | 0.002 |
| hexanoylcarnitine (C6) | Lipid | Fatty Acid Metabolism (Acyl Carnitine, Medium Chain) | -0.60 | 0.14 | 0.002 |
| octanoylcarnitine (C8) | Lipid | Fatty Acid Metabolism (Acyl Carnitine, Medium Chain) | -0.58 | 0.13 | 0.003 |
| laurylcarnitine (C12) | Lipid | Fatty Acid Metabolism (Acyl Carnitine, Medium Chain) | -0.46 | 0.12 | 0.012 |
| nonanoylcarnitine (C9) | Lipid | Fatty Acid Metabolism (Acyl Carnitine, Medium Chain) | -0.64 | 0.16 | 0.012 |
| 5-dodecenoylcarnitine (C12:1) | Lipid | Fatty Acid Metabolism (Acyl Carnitine, Monounsaturated) | -0.44 | 0.12 | 0.029 |
| cis-4-decenoylcarnitine (C10:1) | Lipid | Fatty Acid Metabolism (Acyl Carnitine, Monounsaturated) | -0.39 | 0.12 | 0.041 |
| myristoleoylcarnitine (C14:1)* | Lipid | Fatty Acid Metabolism (Acyl Carnitine, Monounsaturated) | -0.38 | 0.12 | 0.046 |
| dodecadienoate (12:2)* | Lipid | Fatty Acid, Dicarboxylate | -0.45 | 0.13 | 0.029 |
| 3-hydroxymyristate | Lipid | Fatty Acid, Monohydroxy | -0.42 | 0.12 | 0.029 |
| 3-hydroxylaurate | Lipid | Fatty Acid, Monohydroxy | -0.41 | 0.12 | 0.035 |
| docosapentaenoate (n6 DPA; 22:5n6) | Lipid | Long Chain Polyunsaturated Fatty Acid (n3 and n6) | -0.47 | 0.14 | 0.037 |
| 5-dodecenoate (12:1n7) | Lipid | Medium Chain Fatty Acid | -0.41 | 0.12 | 0.035 |
| 1-palmitoyl-2-docosahexaenoyl-GPC (16:0/22:6) | Lipid | Phosphatidylcholine (PC) | -0.67 | 0.15 | 0.002 |
| 1-myristoyl-2-arachidonoyl-GPC (14:0/20:4)* | Lipid | Phosphatidylcholine (PC) | -0.60 | 0.17 | 0.029 |
| glyco-beta-muricholate | Lipid | Primary Bile Acid Metabolism | 0.57 | 0.11 | 0.001 |
| tauro-beta-muricholate | Lipid | Primary Bile Acid Metabolism | 0.45 | 0.13 | 0.033 |
| hyocholate | Lipid | Secondary Bile Acid Metabolism | 0.45 | 0.13 | 0.029 |
| glycohyocholate | Lipid | Secondary Bile Acid Metabolism | 0.46 | 0.14 | 0.033 |
| gamma-glutamylleucine | Peptide | Gamma-glutamyl Amino Acid | -0.54 | 0.16 | 0.029 |
| gamma-glutamylmethionine | Peptide | Gamma-glutamyl Amino Acid | -0.52 | 0.17 | 0.049 |

AA, African American; BMI, Body mass index; BP, blood pressure; CAC, Coronary artery calcium; CVD, Cardiovascular diseases;

LDL-C, Low-density lipoprotein cholesterol SE, Standard error

Model adjusted for age, sex, BMI, smoking status, hypertension status, CVD, date of plasma collection, time between plasma collection and CT exam, HbA1C, LDL-C, statin use

*Indicates compounds that have not been officially confirmed based on a standard, but identified by virtue of their recurrent chromatographic and spectral nature

**Additional file 1: Table SIX. Association between plasma metabolites and CAC in EAs (Additionally adjusted for HbA1C)**

| **Biochemical** | **Super-pathway** | **Sub-pathway** | **β-Estimate** | **SE** | **P_FDR_** |
| --- | --- | --- | --- | --- | --- |
| N-acetylserine | Amino Acid | Glycine, Serine and Threonine Metabolism | 0.47 | 0.14 | 0.039 |
| 1-methyl-4-imidazoleacetate | Amino Acid | Histidine Metabolism | 0.45 | 0.14 | 0.046 |
| isobutyrylcarnitine (C4) | Amino Acid | Leucine, Isoleucine and Valine Metabolism | 0.46 | 0.14 | 0.046 |
| N6-acetyllysine | Amino Acid | Lysine Metabolism | 0.51 | 0.15 | 0.039 |
| 5-hydroxylysine | Amino Acid | Lysine Metabolism | 0.53 | 0.16 | 0.045 |
| 2,3-dihydroxy-5-methylthio-4-pentenoate (DMTPA)* | Amino Acid | Methionine, Cysteine, SAM and Taurine Metabolism | 0.50 | 0.15 | 0.039 |
| lanthionine | Amino Acid | Methionine, Cysteine, SAM and Taurine Metabolism | 0.49 | 0.15 | 0.046 |
| N-acetylphenylalanine | Amino Acid | Phenylalanine Metabolism | 0.56 | 0.15 | 0.017 |
| N-acetyl-isoputreanine | Amino Acid | Polyamine Metabolism | 0.45 | 0.14 | 0.046 |
| glucuronate | Carbohydrate | Aminosugar Metabolism | 0.62 | 0.15 | 0.0067 |
| ascorbic acid 2-sulfate | Cofactors and Vitamins | Ascorbate and Aldarate Metabolism | 0.51 | 0.16 | 0.046 |
| androstenediol (3beta,17beta) monosulfate (2) | Lipid | Androgenic Steroids | -0.52 | 0.16 | 0.046 |
| palmitoleoyl-linoleoyl-glycerol (16:1/18:2) [1]* | Lipid | Diacylglycerol | -0.51 | 0.15 | 0.039 |
| palmitoyl-linoleoyl-glycerol (16:0/18:2) [1]* | Lipid | Diacylglycerol | -0.51 | 0.15 | 0.039 |
| N-palmitoyl-sphinganine (d18:0/16:0) | Lipid | Dihydroceramides | 0.49 | 0.15 | 0.046 |
| palmitoyl dihydrosphingomyelin (d18:0/16:0)* | Lipid | Dihydrosphingomyelins | 0.67 | 0.16 | 0.0067 |
| picolinoylglycine | Lipid | Fatty Acid Metabolism (Acyl Glycine) | 0.43 | 0.13 | 0.045 |
| N-acetyl-2-aminooctanoate* | Lipid | Fatty Acid, Amino | 0.48 | 0.14 | 0.042 |
| 3,4-dihydroxybutyrate | Lipid | Fatty Acid, Dihydroxy | 0.47 | 0.15 | 0.048 |
| 10-undecenoate (11:1n1) | Lipid | Medium Chain Fatty Acid | -0.47 | 0.15 | 0.046 |
| 3-hydroxy-3-methylglutarate | Lipid | Mevalonate Metabolism | 0.80 | 0.16 | 2.85E-04 |
| 1-linolenoylglycerol (18:3) | Lipid | Monoacylglycerol | -0.62 | 0.15 | 0.0067 |
| glycerophosphoinositol* | Lipid | Phospholipid Metabolism | 0.53 | 0.15 | 0.039 |
| 1-(1-enyl-palmitoyl)-2-palmitoleoyl-GPC (P-16:0/16:1)* | Lipid | Plasmalogen | 0.58 | 0.16 | 0.039 |
| campesterol | Lipid | Sterol | 0.96 | 0.30 | 0.046^†^ |
| N6-carbamoylthreonyladenosine | Nucleotide | Purine Metabolism, Adenine containing | 0.44 | 0.14 | 0.046 |
| N2,N2-dimethylguanosine | Nucleotide | Purine Metabolism, Guanine containing | 0.59 | 0.14 | 0.0067 |
| 7-methylguanine | Nucleotide | Purine Metabolism, Guanine containing | 0.58 | 0.16 | 0.039 |
| 2'-O-methyluridine | Nucleotide | Pyrimidine Metabolism, Uracil containing | 0.50 | 0.15 | 0.046 |

BMI, Body mass index; BP, blood pressure; CAC, Coronary artery calcium; CVD, Cardiovascular diseases; EA, European American; LDL-C, Low-density lipoprotein cholesterol SE, Standard error

Model adjusted for age, sex, BMI, smoking status, hypertension status, CVD, date of plasma collection, time between plasma collection and CT exam, HbA1C, LDL-C, statin use

*Indicates compounds that have not been officially confirmed based on a standard, but identified by virtue of their recurrent chromatographic and spectral nature

^†^Indicates results derived from dichotomized metabolite values, i.e. presence vs absence, when >50% of the data were missing

**Additional file 1: Table SX. Significant plasma metabolites in AAs with cross-ancestry replication in EAs (Additionally adjusted for LDL-C and Statin)**

|  |  |  | **African Americans (AAs)** | | |  | **European Americans (EAs)** | | |
| --- | --- | --- | --- | --- | --- | --- | --- | --- | --- |
| **Biochemical** | **Super-pathway** | **Sub-pathway** | **β-Estimate** | **SE** | **P_FDR_** |  | **β-Estimate** | **SE** | **P** |
| dehydroepiandrosterone sulfate (DHEA-S) | Lipid | Androgenic Steroids | -0.45 | 0.13 | 0.018 |  | -0.44 | 0.16 | 0.0052 |
| androsterone sulfate | Lipid | Androgenic Steroids | -0.43 | 0.12 | 0.026 |  | -0.38 | 0.15 | 0.010 |
| androstenediol (3beta,17beta) disulfate (1) | Lipid | Androgenic Steroids | -0.42 | 0.13 | 0.038 |  | -0.41 | 0.16 | 0.013 |
| androstenediol (3alpha, 17alpha) monosulfate (3) | Lipid | Androgenic Steroids | -0.52 | 0.13 | 0.0094 |  | -0.38 | 0.15 | 0.015 |
| androstenediol (3beta,17beta) monosulfate (1) | Lipid | Androgenic Steroids | -0.45 | 0.13 | 0.021 |  | -0.37 | 0.16 | 0.018 |
| epiandrosterone sulfate | Lipid | Androgenic Steroids | -0.42 | 0.12 | 0.026 |  | -0.35 | 0.15 | 0.018 |
| 5alpha-androstan-3alpha,17beta-diol monosulfate (1) | Lipid | Androgenic Steroids | -0.40 | 0.13 | 0.047 |  | -0.36 | 0.16 | 0.024 |
| 1-palmitoyl-2-docosahexaenoyl-GPC (16:0/22:6) | Lipid | Phosphatidylcholine (PC) | -0.69 | 0.15 | 0.0008 |  | -0.31 | 0.16 | 0.049 |
| pregnenetriol sulfate* | Lipid | Pregnenolone Steroids | -0.41 | 0.13 | 0.050 |  | -0.42 | 0.17 | 0.013 |
| glycohyocholate | Lipid | Secondary Bile Acid Metabolism | 0.44 | 0.14 | 0.038 |  | 0.32 | 0.14 | 0.017 |

AA, African American; BMI, Body mass index; CAC, Coronary artery calcium; LDL-C, Low-density lipoprotein cholesterol; SE, Standard error

Model adjusted for age, sex, BMI, smoking status, hypertension status, CVD, duration of diabetes, date of plasma collection, time between plasma collection and CT exam, LDL-C, statin use

*Indicates compounds that have not been officially confirmed based on a standard, but identified by virtue of their recurrent chromatographic and spectral nature

**Additional file 1: Table SXI. Significant plasma metabolites in EAs with cross-ancestry replication in AAs (Additionally adjusted for LDL-C and Statin)**

|  |  |  | **European Americans (EAs)** | | |  | **African Americans (AAs)** | | |
| --- | --- | --- | --- | --- | --- | --- | --- | --- | --- |
| **Biochemical** | **Super-pathway** | **Sub-pathway** | **β-Estimate** | **SE** | **P_FDR_** |  | Estimate | Stderr | fdr_p |
| androstenediol (3beta,17beta) monosulfate (2) | Lipid | Androgenic Steroids | -0.52 | 0.16 | 0.047 |  | -0.33 | 0.12 | 0.0065 |
| 10-undecenoate (11:1n1) | Lipid | Medium Chain Fatty Acid | -0.48 | 0.15 | 0.048 |  | -0.31 | 0.13 | 0.016 |
| pregnen-diol disulfate* | Lipid | Pregnenolone Steroids | -0.50 | 0.16 | 0.047 |  | -0.34 | 0.13 | 0.012 |
| pregnenolone sulfate | Lipid | Pregnenolone Steroids | -0.48 | 0.15 | 0.050 |  | -0.35 | 0.15 | 0.021 |
| 2'-O-methyluridine | Nucleotide | Pyrimidine Metabolism, Uracil containing | 0.48 | 0.15 | 0.048 |  | 0.38 | 0.19 | 0.041 |

BMI, Body mass index; CAC, Coronary artery calcium; EA, European American; LDL-C, Low-density lipoprotein cholesterol; SE, Standard error

Model adjusted for age, sex, BMI, smoking status, hypertension status, CVD, date of plasma collection, time between plasma collection and CT exam, duration of diabetes, LDL-C, statin use

*Indicates compounds that have not been officially confirmed based on a standard, but identified by virtue of their recurrent chromatographic and spectral nature

**Additional file 1: Table SXII. Association between plasma metabolites and CAC (CAC<10 versus CAC≥10) in AAs (Additionally adjusted for LDL-C and Statin)**

| **Biochemical** | **Super-pathway** | **Sub-pathway** | **β-Estimate** | **SE** | **P_FDR_** |
| --- | --- | --- | --- | --- | --- |
| 3-hydroxy-2-ethylpropionate | Amino Acid | Leucine, Isoleucine and Valine Metabolism | -0.50 | 0.13 | 0.015 |
| 5-methylthioadenosine (MTA) | Amino Acid | Polyamine Metabolism | -0.43 | 0.14 | 0.049 |
| maltotriose | Carbohydrate | Glycogen Metabolism | -0.50 | 0.14 | 0.018 |
| bilirubin (E,E)* | Cofactors and Vitamins | Hemoglobin and Porphyrin Metabolism | -0.46 | 0.14 | 0.032 |
| bilirubin (E,Z or Z,E)* | Cofactors and Vitamins | Hemoglobin and Porphyrin Metabolism | -0.45 | 0.15 | 0.046 |
| carotene diol (1) | Cofactors and Vitamins | Vitamin A Metabolism | -0.42 | 0.14 | 0.042 |
| retinal | Cofactors and Vitamins | Vitamin A Metabolism | 0.47 | 0.16 | 0.046 |
| 5alpha-androstan-3beta,17beta-diol disulfate | Lipid | Androgenic Steroids | -0.47 | 0.16 | 0.049 |
| sphingomyelin (d18:0/18:0, d19:0/17:0)* | Lipid | Dihydrosphingomyelins | -0.50 | 0.15 | 0.032 |
| 5-HEPE | Lipid | Eicosanoid | 0.55 | 0.17 | 0.030 |
| oleoyl ethanolamide | Lipid | Endocannabinoid | -0.40 | 0.13 | 0.042 |
| palmitoyl ethanolamide | Lipid | Endocannabinoid | -0.42 | 0.14 | 0.046 |
| (R)-3-hydroxybutyrylcarnitine | Lipid | Fatty Acid Metabolism (Acyl Carnitine, Hydroxy) | -0.38 | 0.12 | 0.036 |
| 3-hydroxydecanoylcarnitine | Lipid | Fatty Acid Metabolism (Acyl Carnitine, Hydroxy) | -0.47 | 0.13 | 0.018 |
| myristoylcarnitine (C14) | Lipid | Fatty Acid Metabolism (Acyl Carnitine, Long Chain Saturated) | -0.50 | 0.14 | 0.019 |
| palmitoylcarnitine (C16) | Lipid | Fatty Acid Metabolism (Acyl Carnitine, Long Chain Saturated) | -0.52 | 0.14 | 0.015 |
| decanoylcarnitine (C10) | Lipid | Fatty Acid Metabolism (Acyl Carnitine, Medium Chain) | -0.58 | 0.14 | 0.011 |
| hexanoylcarnitine (C6) | Lipid | Fatty Acid Metabolism (Acyl Carnitine, Medium Chain) | -0.58 | 0.14 | 0.011 |
| laurylcarnitine (C12) | Lipid | Fatty Acid Metabolism (Acyl Carnitine, Medium Chain) | -0.46 | 0.13 | 0.018 |
| nonanoylcarnitine (C9) | Lipid | Fatty Acid Metabolism (Acyl Carnitine, Medium Chain) | -0.54 | 0.15 | 0.015 |
| octanoylcarnitine (C8) | Lipid | Fatty Acid Metabolism (Acyl Carnitine, Medium Chain) | -0.56 | 0.14 | 0.011 |
| 5-dodecenoylcarnitine (C12:1) | Lipid | Fatty Acid Metabolism (Acyl Carnitine, Monounsaturated) | -0.53 | 0.14 | 0.015 |
| cis-4-decenoylcarnitine (C10:1) | Lipid | Fatty Acid Metabolism (Acyl Carnitine, Monounsaturated) | -0.51 | 0.14 | 0.015 |
| myristoleoylcarnitine (C14:1)* | Lipid | Fatty Acid Metabolism (Acyl Carnitine, Monounsaturated) | -0.39 | 0.13 | 0.043 |
| oleoylcarnitine (C18:1) | Lipid | Fatty Acid Metabolism (Acyl Carnitine, Monounsaturated) | -0.43 | 0.13 | 0.032 |
| palmitoleoylcarnitine (C16:1)* | Lipid | Fatty Acid Metabolism (Acyl Carnitine, Monounsaturated) | -0.42 | 0.14 | 0.045 |
| acetylcarnitine (C2) | Lipid | Fatty Acid Metabolism (Acyl Carnitine, Short Chain) | -0.56 | 0.14 | 0.011 |
| propionylcarnitine (C3) | Lipid | Fatty Acid Metabolism (also BCAA Metabolism) | -0.50 | 0.16 | 0.040 |
| dodecadienoate (12:2)* | Lipid | Fatty Acid, Dicarboxylate | -0.47 | 0.15 | 0.032 |
| 3-hydroxylaurate | Lipid | Fatty Acid, Monohydroxy | -0.44 | 0.13 | 0.030 |
| 3-hydroxymyristate | Lipid | Fatty Acid, Monohydroxy | -0.43 | 0.13 | 0.030 |
| 1-arachidonoyl-GPA (20:4) | Lipid | Lysophospholipid | 0.59 | 0.18 | 0.026 |
| 1-oleoylglycerophosphate (18:1) | Lipid | Lysophospholipid | 0.61 | 0.17 | 0.018 |
| 1-palmitoyl-2-arachidonoyl-GPC (16:0/20:4n6) | Lipid | Phosphatidylcholine (PC) | -0.48 | 0.15 | 0.031 |
| 1-palmitoyl-2-docosahexaenoyl-GPC (16:0/22:6) | Lipid | Phosphatidylcholine (PC) | -0.55 | 0.14 | 0.011 |
| 1-stearoyl-2-arachidonoyl-GPC (18:0/20:4) | Lipid | Phosphatidylcholine (PC) | -0.43 | 0.14 | 0.036 |
| 1-stearoyl-2-arachidonoyl-GPI (18:0/20:4) | Lipid | Phosphatidylinositol (PI) | -0.42 | 0.14 | 0.043 |
| glyco-beta-muricholate | Lipid | Primary Bile Acid Metabolism | 0.43 | 0.13 | 0.032 |
| 7-HOCA | Lipid | Sterol | -0.45 | 0.14 | 0.032 |
| 1-methyladenosine | Nucleotide | Purine Metabolism, Adenine containing | -0.43 | 0.14 | 0.042 |
| phenylacetylcarnitine | Peptide | Acetylated Peptides | -0.50 | 0.14 | 0.019 |
| cyclo(gly-pro) | Peptide | Dipeptide | 0.49 | 0.14 | 0.020 |
| glycylleucine | Peptide | Dipeptide | 0.53 | 0.15 | 0.018 |
| gamma-glutamyl-alpha-lysine | Peptide | Gamma-glutamyl Amino Acid | -0.58 | 0.15 | 0.015 |
| gamma-glutamylglycine | Peptide | Gamma-glutamyl Amino Acid | -0.50 | 0.14 | 0.019 |
| gamma-glutamylhistidine | Peptide | Gamma-glutamyl Amino Acid | -0.51 | 0.14 | 0.018 |
| gamma-glutamylleucine | Peptide | Gamma-glutamyl Amino Acid | -0.58 | 0.16 | 0.015 |
| gamma-glutamylmethionine | Peptide | Gamma-glutamyl Amino Acid | -0.58 | 0.15 | 0.015 |
| gamma-glutamylphenylalanine | Peptide | Gamma-glutamyl Amino Acid | -0.52 | 0.15 | 0.019 |

AA, African American; BMI, Body mass index; CAC, Coronary artery calcium; LDL-C, Low-density lipoprotein cholesterol; SE, Standard error

Model adjusted for age, sex, BMI, smoking status, hypertension status, CVD, duration of diabetes, LDL-C, statin use

*Indicates compounds that have not been officially confirmed based on a standard, but identified by virtue of their recurrent chromatographic and spectral nature

**Additional file 1: Table SXIII. Association between plasma metabolites and CAC (CAC<10 versus CAC≥10) in EAs (Additionally adjusted for LDL-C and Statin)**

| **Biochemical** | **Super-pathway** | **Sub-pathway** | **β-Estimate** | **SE** | **P_FDR_** |
| --- | --- | --- | --- | --- | --- |
| glycerophosphoinositol* | Lipid | Phospholipid Metabolism | 1.10 | 0.28 | 0.035 |
| palmitoyl dihydrosphingomyelin (d18:0/16:0)* | Lipid | Dihydrosphingomyelins | 1.32 | 0.32 | 0.029 |

BMI, Body mass index; CAC, Coronary artery calcium; EA, European American; LDL-C, Low-density lipoprotein cholesterol; SE, Standard error; TCA, tricarboxylic acid cycle

Model adjusted for age, sex, BMI, smoking status, hypertension status, CVD, duration of diabetes, LDL-C, statin use

*Indicates compounds that have not been officially confirmed based on a standard, but identified by virtue of their recurrent chromatographic and spectral nature

**Additional file 1: Table SⅩⅣ. Mediation analysis to delineate the effects of diabetes duration in AAs (Additionally adjusted for LDL-C and Statin)**

|  |  |  | Model Including Diabetes Duration | | |  | Model Excluding Diabetes Duration | | | Mediation Effect |
| --- | --- | --- | --- | --- | --- | --- | --- | --- | --- | --- |
| **Biochemical** | **Super-pathway** | **Sub-pathway** | Estimate | Stderr | P_FDR |  | Estimate | Stderr | P_FDR |  |
| 3-hydroxy-2-ethylpropionate | Amino Acid | Leucine, Isoleucine and Valine Metabolism | -0.41 | 0.12 | 0.033 |  | -0.37 | 0.12 | 0.064 | -10.16 |
| 5alpha-androstan-3alpha,17beta-diol monosulfate (1) | Lipid | Androgenic Steroids | -0.40 | 0.13 | 0.047 |  | -0.39 | 0.13 | 0.064 | -2.71 |
| androstenediol (3alpha, 17alpha) monosulfate (3) | Lipid | Androgenic Steroids | -0.52 | 0.13 | 0.0094 |  | -0.49 | 0.14 | 0.018 | -6.90 |
| androstenediol (3beta,17beta) disulfate (1) | Lipid | Androgenic Steroids | -0.42 | 0.13 | 0.038 |  | -0.42 | 0.13 | 0.040 | 0.35 |
| androstenediol (3beta,17beta) monosulfate (1) | Lipid | Androgenic Steroids | -0.45 | 0.13 | 0.021 |  | -0.46 | 0.13 | 0.018 | 2.01 |
| androsterone sulfate | Lipid | Androgenic Steroids | -0.43 | 0.12 | 0.026 |  | -0.41 | 0.13 | 0.045 | -5.25 |
| dehydroepiandrosterone sulfate (DHEA-S) | Lipid | Androgenic Steroids | -0.45 | 0.13 | 0.018 |  | -0.46 | 0.12 | 0.018 | 0.64 |
| epiandrosterone sulfate | Lipid | Androgenic Steroids | -0.42 | 0.12 | 0.026 |  | -0.40 | 0.12 | 0.045 | -5.41 |
| myristoylcarnitine (C14) | Lipid | Fatty Acid Metabolism (Acyl Carnitine, Long Chain Saturated) | -0.51 | 0.15 | 0.026 |  | -0.51 | 0.15 | 0.032 | 0.13 |
| decanoylcarnitine (C10) | Lipid | Fatty Acid Metabolism (Acyl Carnitine, Medium Chain) | -0.68 | 0.14 | 7.97E-04 |  | -0.66 | 0.14 | 0.0016 | -3.50 |
| hexanoylcarnitine (C6) | Lipid | Fatty Acid Metabolism (Acyl Carnitine, Medium Chain) | -0.59 | 0.14 | 0.0024 |  | -0.59 | 0.14 | 0.0030 | -0.68 |
| laurylcarnitine (C12) | Lipid | Fatty Acid Metabolism (Acyl Carnitine, Medium Chain) | -0.48 | 0.12 | 0.0039 |  | -0.48 | 0.12 | 0.0065 | -1.36 |
| nonanoylcarnitine (C9) | Lipid | Fatty Acid Metabolism (Acyl Carnitine, Medium Chain) | -0.68 | 0.16 | 0.0039 |  | -0.66 | 0.17 | 0.0076 | -2.41 |
| octanoylcarnitine (C8) | Lipid | Fatty Acid Metabolism (Acyl Carnitine, Medium Chain) | -0.61 | 0.13 | 8.68E-04 |  | -0.59 | 0.14 | 0.0030 | -4.04 |
| 5-dodecenoylcarnitine (C12:1) | Lipid | Fatty Acid Metabolism (Acyl Carnitine, Monounsaturated) | -0.46 | 0.13 | 0.016 |  | -0.44 | 0.13 | 0.030 | -6.19 |
| cis-4-decenoylcarnitine (C10:1) | Lipid | Fatty Acid Metabolism (Acyl Carnitine, Monounsaturated) | -0.41 | 0.12 | 0.029 |  | -0.39 | 0.12 | 0.045 | -4.04 |
| myristoleoylcarnitine (C14:1)* | Lipid | Fatty Acid Metabolism (Acyl Carnitine, Monounsaturated) | -0.40 | 0.12 | 0.029 |  | -0.38 | 0.12 | 0.045 | -4.33 |
| dodecadienoate (12:2)* | Lipid | Fatty Acid, Dicarboxylate | -0.47 | 0.13 | 0.018 |  | -0.47 | 0.13 | 0.019 | 0.04 |
| 3-hydroxylaurate | Lipid | Fatty Acid, Monohydroxy | -0.45 | 0.12 | 0.016 |  | -0.44 | 0.12 | 0.018 | -0.92 |
| 3-hydroxymyristate | Lipid | Fatty Acid, Monohydroxy | -0.46 | 0.12 | 0.0094 |  | -0.45 | 0.12 | 0.017 | -1.89 |
| 5-dodecenoate (12:1n7) | Lipid | Medium Chain Fatty Acid | -0.38 | 0.12 | 0.047 |  | -0.39 | 0.12 | 0.045 | 0.73 |
| 1-myristoyl-2-arachidonoyl-GPC (14:0/20:4)* | Lipid | Phosphatidylcholine (PC) | -0.55 | 0.18 | 0.047 |  | -0.58 | 0.18 | 0.039 | 5.14 |
| 1-oleoyl-2-docosahexaenoyl-GPC (18:1/22:6)* | Lipid | Phosphatidylcholine (PC) | -0.38 | 0.12 | 0.047 |  | -0.34 | 0.12 | 0.100 | -12.51 |
| 1-palmitoyl-2-docosahexaenoyl-GPC (16:0/22:6) | Lipid | Phosphatidylcholine (PC) | -0.69 | 0.15 | 7.97E-04 |  | -0.70 | 0.15 | 7.89E-04 | 0.88 |
| pregnenetriol sulfate* | Lipid | Pregnenolone Steroids | -0.41 | 0.13 | 0.050 |  | -0.42 | 0.13 | 0.040 | 2.93 |
| glyco-beta-muricholate | Lipid | Primary Bile Acid Metabolism | 0.54 | 0.11 | 7.97E-04 |  | 0.56 | 0.11 | 7.04E-04 | 4.56 |
| tauro-beta-muricholate | Lipid | Primary Bile Acid Metabolism | 0.40 | 0.13 | 0.047 |  | 0.41 | 0.13 | 0.045 | 3.20 |
| glycohyocholate | Lipid | Secondary Bile Acid Metabolism | 0.44 | 0.14 | 0.038 |  | 0.45 | 0.14 | 0.040 | 2.92 |
| hyocholate | Lipid | Secondary Bile Acid Metabolism | 0.45 | 0.13 | 0.022 |  | 0.46 | 0.13 | 0.018 | 1.24 |
| gamma-glutamyl-alpha-lysine | Peptide | Gamma-glutamyl Amino Acid | -0.59 | 0.17 | 0.022 |  | -0.57 | 0.17 | 0.032 | -3.14 |
| gamma-glutamylleucine | Peptide | Gamma-glutamyl Amino Acid | -0.57 | 0.15 | 0.0098 |  | -0.58 | 0.15 | 0.012 | 0.71 |
| gamma-glutamylmethionine | Peptide | Gamma-glutamyl Amino Acid | -0.53 | 0.17 | 0.047 |  | -0.55 | 0.17 | 0.040 | 3.06 |
| gamma-glutamylvaline | Peptide | Gamma-glutamyl Amino Acid | -0.37 | 0.12 | 0.047 |  | -0.38 | 0.12 | 0.045 | 2.33 |

BMI, Body mass index; CAC, Coronary artery calcium; EA, European American; LDL-C, Low-density lipoprotein cholesterol; SE, Standard error; TCA, tricarboxylic acid cycle

Model adjusted for age, sex, BMI, smoking status, hypertension status, CVD, duration of diabetes, date of plasma collection, time between plasma collection and CT exam, LDL-C, statin use

*Indicates compounds that have not been officially confirmed based on a standard, but identified by virtue of their recurrent chromatographic and spectral nature

**Additional file 1: Table SⅩⅤ. Mediation analysis to delineate the effects of diabetes duration in EAs (Additionally adjusted for LDL-C and Statin)**

|  |  |  | Model Including Diabetes Duration | | |  | Model Excluding Diabetes Duration | | |  |
| --- | --- | --- | --- | --- | --- | --- | --- | --- | --- | --- |
| **Biochemical** | **Super-pathway** | **Sub-pathway** | Estimate | Stderr | P_FDR |  | Estimate | Stderr | P_FDR | Mediation Effect |
| 1-methyl-4-imidazoleacetate | Amino Acid | Histidine Metabolism | 0.43 | 0.14 | 0.049 |  | 0.46 | 0.14 | 0.046 | 7.72 |
| isobutyrylcarnitine (C4) | Amino Acid | Leucine, Isoleucine and Valine Metabolism | 0.44 | 0.14 | 0.049 |  | 0.43 | 0.14 | 0.073 | -2.00 |
| N-acetylleucine | Amino Acid | Leucine, Isoleucine and Valine Metabolism | 0.50 | 0.16 | 0.048 |  | 0.50 | 0.16 | 0.070 | -1.46 |
| 5-hydroxylysine | Amino Acid | Lysine Metabolism | 0.51 | 0.16 | 0.048 |  | 0.55 | 0.16 | 0.038 | 8.18 |
| N6-acetyllysine | Amino Acid | Lysine Metabolism | 0.45 | 0.14 | 0.045 |  | 0.45 | 0.14 | 0.046 | 0.83 |
| 2,3-dihydroxy-5-methylthio-4-pentenoate (DMTPA)* | Amino Acid | Methionine, Cysteine, SAM and Taurine Metabolism | 0.50 | 0.14 | 0.039 |  | 0.51 | 0.14 | 0.034 | 1.72 |
| methionine sulfoxide | Amino Acid | Methionine, Cysteine, SAM and Taurine Metabolism | 0.54 | 0.17 | 0.047 |  | 0.49 | 0.17 | 0.079 | -11.00 |
| N-acetylphenylalanine | Amino Acid | Phenylalanine Metabolism | 0.58 | 0.14 | 0.0088 |  | 0.59 | 0.15 | 0.0084 | 2.17 |
| N-acetyl-isoputreanine | Amino Acid | Polyamine Metabolism | 0.46 | 0.14 | 0.045 |  | 0.49 | 0.14 | 0.033 | 6.40 |
| picolinate | Amino Acid | Tryptophan Metabolism | 0.42 | 0.12 | 0.040 |  | 0.41 | 0.12 | 0.046 | -3.67 |
| erythronate* | Carbohydrate | Aminosugar Metabolism | 0.50 | 0.16 | 0.048 |  | 0.55 | 0.15 | 0.029 | 7.73 |
| glucuronate | Carbohydrate | Aminosugar Metabolism | 0.60 | 0.15 | 0.012 |  | 0.62 | 0.15 | 0.0084 | 4.29 |
| arabinose | Carbohydrate | Pentose Metabolism | 0.48 | 0.15 | 0.049 |  | 0.47 | 0.15 | 0.058 | -1.08 |
| ascorbic acid 2-sulfate | Cofactors and Vitamins | Ascorbate and Aldarate Metabolism | 0.50 | 0.16 | 0.049 |  | 0.52 | 0.16 | 0.058 | 3.23 |
| androstenediol (3beta,17beta) monosulfate (2) | Lipid | Androgenic Steroids | -0.52 | 0.16 | 0.047 |  | -0.51 | 0.16 | 0.058 | -1.91 |
| N-palmitoyl-sphinganine (d18:0/16:0) | Lipid | Dihydroceramides | 0.56 | 0.16 | 0.039 |  | 0.53 | 0.15 | 0.038 | -4.99 |
| palmitoyl dihydrosphingomyelin (d18:0/16:0)* | Lipid | Dihydrosphingomyelins | 0.68 | 0.16 | 0.0068 |  | 0.66 | 0.16 | 0.0084 | -3.04 |
| picolinoylglycine | Lipid | Fatty Acid Metabolism (Acyl Glycine) | 0.44 | 0.12 | 0.039 |  | 0.43 | 0.13 | 0.042 | -2.38 |
| N-acetyl-2-aminooctanoate* | Lipid | Fatty Acid, Amino | 0.49 | 0.14 | 0.040 |  | 0.54 | 0.14 | 0.022 | 9.83 |
| 3,4-dihydroxybutyrate | Lipid | Fatty Acid, Dihydroxy | 0.48 | 0.15 | 0.047 |  | 0.52 | 0.15 | 0.036 | 7.17 |
| 13-HODE + 9-HODE | Lipid | Fatty Acid, Monohydroxy | 0.56 | 0.17 | 0.047 |  | 0.51 | 0.17 | 0.079 | -9.60 |
| 1-(1-enyl-oleoyl)-GPE (P-18:1)* | Lipid | Lysoplasmalogen | 0.58 | 0.16 | 0.039 |  | 0.53 | 0.17 | 0.058 | -9.29 |
| 1-(1-enyl-palmitoyl)-GPE (P-16:0)* | Lipid | Lysoplasmalogen | 0.57 | 0.18 | 0.047 |  | 0.48 | 0.18 | 0.10 | -17.31 |
| 10-undecenoate (11:1n1) | Lipid | Medium Chain Fatty Acid | -0.48 | 0.15 | 0.048 |  | -0.47 | 0.15 | 0.046 | -0.66 |
| 3-hydroxy-3-methylglutarate | Lipid | Mevalonate Metabolism | 0.80 | 0.16 | 3.20E-04 |  | 0.81 | 0.15 | 1.24E-04 | 2.35 |
| 1-linolenoylglycerol (18:3) | Lipid | Monoacylglycerol | -0.62 | 0.15 | 0.0088 |  | -0.61 | 0.15 | 0.011 | -1.03 |
| glycerophosphoinositol* | Lipid | Phospholipid Metabolism | 0.57 | 0.15 | 0.026 |  | 0.53 | 0.16 | 0.046 | -7.42 |
| 1-(1-enyl-palmitoyl)-2-palmitoleoyl-GPC (P-16:0/16:1)* | Lipid | Plasmalogen | 0.51 | 0.16 | 0.049 |  | 0.44 | 0.16 | 0.10 | -16.13 |
| pregnen-diol disulfate* | Lipid | Pregnenolone Steroids | -0.50 | 0.16 | 0.047 |  | -0.46 | 0.16 | 0.079 | -10.05 |
| pregnenolone sulfate | Lipid | Pregnenolone Steroids | -0.48 | 0.15 | 0.050 |  | -0.47 | 0.16 | 0.078 | -2.21 |
| 5alpha-pregnan-3beta,20alpha-diol disulfate | Lipid | Progestin Steroids | -0.44 | 0.13 | 0.040 |  | -0.39 | 0.13 | 0.076 | -14.71 |
| 5alpha-pregnan-diol disulfate | Lipid | Progestin Steroids | -0.90 | 0.28 | 0.047^†^ |  | -0.80 | 0.28 | 0.086 | -11.72 |
| campesterol | Lipid | Sterol | 0.97 | 0.30 | 0.047^†^ |  | 0.95 | 0.31 | 0.062 | -1.86 |
| 7-methylguanine | Nucleotide | Purine Metabolism, Guanine containing | 0.53 | 0.16 | 0.045 |  | 0.55 | 0.16 | 0.042 | 3.67 |
| N2,N2-dimethylguanosine | Nucleotide | Purine Metabolism, Guanine containing | 0.55 | 0.14 | 0.012 |  | 0.57 | 0.14 | 0.0084 | 4.06 |
| 2'-O-methyluridine | Nucleotide | Pyrimidine Metabolism, Uracil containing | 0.48 | 0.15 | 0.048 |  | 0.48 | 0.16 | 0.066 | 0.21 |

BMI, Body mass index; CAC, Coronary artery calcium; EA, European American; LDL-C, Low-density lipoprotein cholesterol; SE, Standard error; TCA, tricarboxylic acid cycle

Model adjusted for age, sex, BMI, smoking status, hypertension status, CVD, duration of diabetes, date of plasma collection, time between plasma collection and CT exam, LDL-C, statin use

*Indicates compounds that have not been officially confirmed based on a standard, but identified by virtue of their recurrent chromatographic and spectral nature

^†^Indicates results derived from dichotomized metabolite values, i.e. presence vs absence, when >50% of the data were missing

**Additional file 1: Table SXⅥ. Characteristics of DHS Replication Cohort**

| **Characteristic** | **African American** | | **European American** | | ***P*-value^a^** |
| --- | --- | --- | --- | --- | --- |
|  | N | Mean ± SD or N (%) | N | Mean ± SD or N (%) |  |
| Male, N (%) | 186 | 74, (39.8) | 514 | 238 (46.3) | 0.148 |
| Age (years) | 186 | 51.9 ± 8.9 | 514 | 62.5 ± 9.3 | **<0.001** |
| Education, N (%) | 182 |  | 508 |  | **<0.001** |
| Less Than High School |  | 27 (14.8) |  | 130 (25.6) |  |
| High School Graduated |  | 88 (48.4) |  | 261 (51.4) |  |
| Above High School |  | 67 (36.8) |  | 117 (23.0) |  |
| Smoking Status, N (%) | 186 |  | 513 |  | 0.744 |
| Never |  | 83 (44.6) |  | 205 (40.0) |  |
| Former |  | 61 (32.8) |  | 211 (41.1) |  |
| Current |  | 42 (22.6) |  | 97 (18.9) |  |
| BMI (kg/m^2^) | 186 | 37.2 9.7) | 514 | 31.5 (6.3) | **<0.001** |
| Total Cholesterol (mg/dL) | 183 | 187.9 ± 46.3 | 507 | 185.0 ± 41.4 | 0.454 |
| LDL-C (mg/dL) | 175 | 113.1 ± 36.0 | 481 | 103.3 ± 31.7 | **<0.001** |
| HDL-C (mg/dL) | 183 | 48.0 ± 13.4 | 507 | 43.2 ± 12.1 | **0.001** |
| Triglyceride^b^ (mg/dL) | 183 | 110 (81, 149) | 507 | 169 (116, 235) | **0.002** |
| Systolic Blood Pressure (mm Hg) | 186 | 131.8 ± 20.2 | 512 | 138.9 ± 20.0 | **<0.001** |
| Diastolic Blood Pressure (mm Hg) | 186 | 79.8 ± 11.3 | 512 | 72.6 ± 10.7 | **<0.001** |
| Hypertension, N (%) | 186 | 147 (79.0) | 514 | 427 (83.1) | 0.219 |
| Diabetes duration (years) | 186 | 9.0 ± 6.5 | 509 | 8.5 ± 7.3 | 0.375 |
| Fasting glucose (mg/dL) | 183 | 149.8 ± 65.0 | 509 | 135.1 ± 60.0 | 0.006 |
| HbA1C (%) | 180 | 8.1 ± 2.1 | 507 | 7.2 ± 1.6 | **<0.001** |
| CVD (%) | 186 | 58 (31.2) | 514 | 51 (9.9) | **<0.001** |
| CAC^b^ | 181 | 7 (0, 95) | 492 | 434.5 (41, 7333.5) | **<0.001** |

BMI, Body Mass Index; CAC, Coronary artery calcium; CVD, Cardiovascular disease; DHS, Diabetes Heart Study; HDL-C, High-density lipoprotein cholesterol; LDL-C, Low-density lipoprotein cholesterol

^a^P-value by a marginal model with generalized estimating equations

^b^Median (Interquartile range)

**Additional file 1: Table SXVII. Replication of plasma metabolites associated in AAs (Additionally adjusted for LDL-C and Statin)**

|  |  |  | **African Americans (AAs)** | | |  | **Replication** | | |  | **Joint Analysis** | | |
| --- | --- | --- | --- | --- | --- | --- | --- | --- | --- | --- | --- | --- | --- |
| **Biochemical** | **Super-pathway** | **Sub-pathway** | **β-Estimate** | **SE** | **P_FDR_** |  | **β-Estimate** | **SE** | **P** |  | **β-Estimate** | **SE** | **P** |
| dehydroepiandrosterone sulfate (DHEA-S) | Lipid | Androgenic Steroids | -0.45 | 0.13 | **0.018** |  | -0.22 | 0.21 | 0.31 |  | -0.38 | 0.12 | **0.0011** |
| androsterone sulfate | Lipid | Androgenic Steroids | -0.43 | 0.12 | **0.026** |  | 0.04 | 0.20 | 0.83 |  | -0.26 | 0.11 | 0.017 |
| androstenediol (3beta,17beta) disulfate (1) | Lipid | Androgenic Steroids | -0.42 | 0.13 | **0.038** |  | -0.15 | 0.20 | 0.44 |  | -0.33 | 0.11 | 0.0040 |
| androstenediol (3alpha, 17alpha) monosulfate (3) | Lipid | Androgenic Steroids | -0.52 | 0.13 | **0.0094** |  | 0.17 | 0.21 | 0.44 |  | -0.26 | 0.12 | 0.026 |
| androstenediol (3beta,17beta) monosulfate (1) | Lipid | Androgenic Steroids | -0.45 | 0.13 | **0.021** |  | -0.17 | 0.21 | 0.42 |  | -0.36 | 0.12 | 0.0023 |
| epiandrosterone sulfate | Lipid | Androgenic Steroids | -0.42 | 0.12 | **0.026** |  | -0.02 | 0.21 | 0.91 |  | -0.29 | 0.11 | 0.0072 |
| 5alpha-androstan-3alpha,17beta-diol monosulfate (1) | Lipid | Androgenic Steroids | -0.40 | 0.13 | **0.047** |  | 0.12 | 0.20 | 0.54 |  | -0.25 | 0.11 | 0.023 |
| 1-palmitoyl-2-docosahexaenoyl-GPC (16:0/22:6) | Lipid | Phosphatidylcholine (PC) | -0.69 | 0.15 | **0.0008** |  | -0.23 | 0.22 | 0.29 |  | -0.69 | 0.13 | **1.46E-07** |
| pregnenetriol sulfate* | Lipid | Pregnenolone Steroids | -0.41 | 0.13 | **0.050** |  | -0.08 | 0.20 | 0.69 |  | -0.32 | 0.12 | 0.0070 |
| glycohyocholate | Lipid | Secondary Bile Acid Metabolism | 0.44 | 0.14 | **0.038** |  | -0.06 | 0.20 | 0.75 |  | 0.22 | 0.11 | 0.050 |
| tauro-beta-muricholate | Lipid | Primary Bile Acid Metabolism | 0.40 | 0.13 | **0.047** |  | -0.27 | 0.22 | 0.21 |  | 0.09 | 0.11 | 0.41 |
| myristoylcarnitine (C14) | Lipid | Fatty Acid Metabolism (Acyl Carnitine, Long Chain Saturated) | -0.51 | 0.15 | **0.026** |  | -0.14 | 0.19 | 0.47 |  | -0.47 | 0.12 | **1.63E-04** |
| 1-myristoyl-2-arachidonoyl-GPC (14:0/20:4)* | Lipid | Phosphatidylcholine (PC) | -0.55 | 0.18 | **0.047** |  | -0.20 | 0.20 | 0.33 |  | -0.39 | 0.14 | 0.0040 |
| 1-oleoyl-2-docosahexaenoyl-GPC (18:1/22:6)* | Lipid | Phosphatidylcholine (PC) | -0.38 | 0.12 | **0.047** |  | -0.35 | 0.20 | 0.080 |  | -0.33 | 0.11 | 0.0024 |
| 5-dodecenoate (12:1n7) | Lipid | Medium Chain Fatty Acid | -0.38 | 0.12 | **0.047** |  | -0.09 | 0.19 | 0.65 |  | -0.28 | 0.10 | 0.0068 |
| glyco-beta-muricholate | Lipid | Primary Bile Acid Metabolism | 0.54 | 0.11 | **0.0008** |  | -0.27 | 0.20 | 0.17 |  | 0.27 | 0.10 | 0.0079 |
| 3-hydroxy-2-ethylpropionate | Amino Acid | Leucine, Isoleucine and Valine Metabolism | -0.41 | 0.12 | **0.033** |  | 0.35 | 0.20 | 0.081 |  | -0.21 | 0.12 | 0.065 |
| dodecadienoate (12:2)* | Lipid | Fatty Acid, Dicarboxylate | -0.47 | 0.13 | **0.018** |  | -0.06 | 0.19 | 0.75 |  | -0.36 | 0.11 | **0.0010** |
| decanoylcarnitine (C10) | Lipid | Fatty Acid Metabolism (Acyl Carnitine, Medium Chain) | -0.68 | 0.14 | **0.0008** |  | 0.00 | 0.20 | 0.99 |  | -0.48 | 0.13 | **1.42E-04** |
| hyocholate | Lipid | Secondary Bile Acid Metabolism | 0.45 | 0.13 | **0.022** |  | -0.22 | 0.20 | 0.28 |  | 0.20 | 0.11 | 0.072 |
| 3-hydroxylaurate | Lipid | Fatty Acid, Monohydroxy | -0.45 | 0.12 | **0.016** |  | -0.08 | 0.19 | 0.66 |  | -0.35 | 0.11 | **8.80E-04** |
| 5-dodecenoylcarnitine (C12:1) | Lipid | Fatty Acid Metabolism (Acyl Carnitine, Monounsaturated) | -0.46 | 0.13 | **0.016** |  | -0.01 | 0.18 | 0.95 |  | -0.37 | 0.11 | **8.66E-04** |
| myristoleoylcarnitine (C14:1)* | Lipid | Fatty Acid Metabolism (Acyl Carnitine, Monounsaturated) | -0.40 | 0.12 | **0.029** |  | -0.07 | 0.18 | 0.70 |  | -0.33 | 0.10 | 0.0017 |
| octanoylcarnitine (C8) | Lipid | Fatty Acid Metabolism (Acyl Carnitine, Medium Chain) | -0.61 | 0.13 | **0.0009** |  | 0.05 | 0.19 | 0.78 |  | -0.48 | 0.12 | **4.17E-05** |
| gamma-glutamylmethionine | Peptide | Gamma-glutamyl Amino Acid | -0.53 | 0.17 | **0.047** |  | 0.00 | 0.20 | 1.00 |  | -0.37 | 0.14 | 0.0075 |
| hexanoylcarnitine (C6) | Lipid | Fatty Acid Metabolism (Acyl Carnitine, Medium Chain) | -0.59 | 0.14 | **0.0024** |  | 0.11 | 0.20 | 0.59 |  | -0.55 | 0.12 | **5.23E-06** |
| gamma-glutamylvaline | Peptide | Gamma-glutamyl Amino Acid | -0.37 | 0.12 | **0.047** |  | 0.08 | 0.20 | 0.68 |  | -0.35 | 0.10 | **3.53E-04** |
| laurylcarnitine (C12) | Lipid | Fatty Acid Metabolism (Acyl Carnitine, Medium Chain) | -0.48 | 0.12 | **0.0039** |  | -0.17 | 0.19 | 0.37 |  | -0.42 | 0.10 | **4.33E-05** |
| cis-4-decenoylcarnitine (C10:1) | Lipid | Fatty Acid Metabolism (Acyl Carnitine, Monounsaturated) | -0.41 | 0.12 | **0.029** |  | -0.01 | 0.19 | 0.97 |  | -0.32 | 0.11 | 0.0031 |
| 3-hydroxymyristate | Lipid | Fatty Acid, Monohydroxy | -0.46 | 0.12 | **0.0094** |  | 0.05 | 0.19 | 0.78 |  | -0.26 | 0.10 | 0.011 |
| gamma-glutamyl-alpha-lysine | Peptide | Gamma-glutamyl Amino Acid | -0.59 | 0.17 | **0.022** |  | -0.16 | 0.21 | 0.44 |  | -0.65 | 0.14 | **2.32E-06** |
| gamma-glutamylleucine | Peptide | Gamma-glutamyl Amino Acid | -0.57 | 0.15 | **0.0098** |  | -0.01 | 0.20 | 0.96 |  | -0.50 | 0.13 | **1.09E-04** |
| nonanoylcarnitine (C9) | Lipid | Fatty Acid Metabolism (Acyl Carnitine, Medium Chain) | -0.68 | 0.16 | **0.0039** |  | 0.02 | 0.20 | 0.94 |  | -0.31 | 0.14 | 0.023 |

AA, African American; BMI, Body mass index; CAC, Coronary artery calcium; LDL-C, Low-density lipoprotein cholesterol; SE, Standard error

Model adjusted for age, sex, BMI, smoking status, hypertension status, CVD, duration of diabetes, date of plasma collection, time between plasma collection and CT exam, LDL-C, statin use

*Indicates compounds that have not been officially confirmed based on a standard, but identified by virtue of their recurrent chromatographic and spectral nature

Statistical significance (**bold**) in the Discovery cohort was defined by a False Discovery Rate (FDR) P-value (P_FDR_) ≤0.05, in the Replication cohort by a nominal P≤0.05, and in the Joint Analysis using a Bonferroni correction, i.e. 0.05/33 or P≤0.0015.

**Additional file 1: Table SXVIII. Replication of plasma metabolites associated in EAs (Additionally adjusted for LDL-C and Statin)**

|  |  |  | **European Americans (EAs)** | | |  | **Replication** | | |  | **Joint Analysis** | | |
| --- | --- | --- | --- | --- | --- | --- | --- | --- | --- | --- | --- | --- | --- |
| **Biochemical** | **Super-pathway** | **Sub-pathway** | **β-Estimate** | **SE** | **P_FDR_** |  | **β-Estimate** | **SE** | **P** |  | **β-Estimate** | **SE** | **P** |
| 1-methyl-4-imidazoleacetate | Amino Acid | Histidine Metabolism | 0.43 | 0.14 | **0.049** |  | 0.13 | 0.12 | 0.29 |  | 0.27 | 0.09 | 0.0036 |
| isobutyrylcarnitine (C4) | Amino Acid | Leucine, Isoleucine and Valine Metabolism | 0.44 | 0.14 | **0.049** |  | 0.10 | 0.11 | 0.39 |  | 0.22 | 0.09 | 0.021 |
| N-acetylleucine | Amino Acid | Leucine, Isoleucine and Valine Metabolism | 0.50 | 0.16 | **0.048** |  | 0.09 | 0.11 | 0.44 |  | 0.25 | 0.09 | 0.0062 |
| N6-acetyllysine | Amino Acid | Lysine Metabolism | 0.45 | 0.14 | **0.045** |  | 0.26 | 0.10 | **0.012** |  | 0.35 | 0.08 | **1.89E-05** |
| 5-hydroxylysine | Amino Acid | Lysine Metabolism | 0.51 | 0.16 | **0.048** |  | 0.08 | 0.10 | 0.41 |  | 0.25 | 0.08 | 0.0027 |
| methionine sulfoxide | Amino Acid | Methionine, Cysteine, SAM and Taurine Metabolism | 0.54 | 0.17 | **0.047** |  | 0.15 | 0.11 | 0.18 |  | 0.23 | 0.09 | 0.014 |
| 2,3-dihydroxy-5-methylthio-4-pentenoate (DMTPA)* | Amino Acid | Methionine, Cysteine, SAM and Taurine Metabolism | 0.50 | 0.14 | **0.039** |  | 0.14 | 0.11 | 0.21 |  | 0.28 | 0.09 | **0.0010** |
| N-acetylphenylalanine | Amino Acid | Phenylalanine Metabolism | 0.58 | 0.14 | **0.0088** |  | 0.05 | 0.11 | 0.64 |  | 0.26 | 0.09 | 0.0031 |
| N-acetyl-isoputreanine | Amino Acid | Polyamine Metabolism | 0.46 | 0.14 | **0.045** |  | 0.27 | 0.11 | **0.011** |  | 0.35 | 0.08 | **1.35E-05** |
| picolinate | Amino Acid | Tryptophan Metabolism | 0.42 | 0.12 | **0.040** |  | 0.03 | 0.12 | 0.80 |  | 0.17 | 0.09 | 0.055 |
| erythronate* | Carbohydrate | Aminosugar Metabolism | 0.50 | 0.16 | **0.048** |  | 0.29 | 0.13 | **0.025** |  | 0.33 | 0.10 | **0.0011** |
| glucuronate | Carbohydrate | Aminosugar Metabolism | 0.60 | 0.15 | **0.012** |  | 0.19 | 0.10 | 0.05 |  | 0.32 | 0.08 | **5.63E-05** |
| arabinose | Carbohydrate | Pentose Metabolism | 0.48 | 0.15 | **0.049** |  | 0.01 | 0.11 | 0.89 |  | 0.14 | 0.09 | 0.12 |
| ascorbic acid 2-sulfate | Cofactors and Vitamins | Ascorbate and Aldarate Metabolism | 0.50 | 0.16 | **0.049** |  | 0.02 | 0.11 | 0.83 |  | 0.16 | 0.10 | 0.096 |
| androstenediol (3beta,17beta) monosulfate (2) | Lipid | Androgenic Steroids | -0.52 | 0.16 | **0.047** |  | -0.11 | 0.11 | 0.30 |  | -0.21 | 0.09 | 0.018 |
| N-palmitoyl-sphinganine (d18:0/16:0) | Lipid | Dihydroceramides | 0.56 | 0.16 | **0.039** |  | 0.35 | 0.13 | **0.0060** |  | 0.41 | 0.10 | **3.68E-05** |
| palmitoyl dihydrosphingomyelin (d18:0/16:0)* | Lipid | Dihydrosphingomyelins | 0.68 | 0.16 | **0.0068** |  | 0.33 | 0.15 | **0.031** |  | 0.42 | 0.11 | **2.63E-04** |
| picolinoylglycine | Lipid | Fatty Acid Metabolism (Acyl Glycine) | 0.44 | 0.12 | **0.039** |  | -0.01 | 0.11 | 0.91 |  | 0.14 | 0.09 | 0.091 |
| N-acetyl-2-aminooctanoate* | Lipid | Fatty Acid, Amino | 0.49 | 0.14 | **0.040** |  | -0.06 | 0.10 | 0.54 |  | 0.15 | 0.08 | 0.077 |
| 3,4-dihydroxybutyrate | Lipid | Fatty Acid, Dihydroxy | 0.48 | 0.15 | **0.047** |  | 0.20 | 0.13 | 0.12 |  | 0.29 | 0.10 | 0.0030 |
| 13-HODE + 9-HODE | Lipid | Fatty Acid, Monohydroxy | 0.56 | 0.17 | **0.047** |  | 0.28 | 0.11 | **0.014** |  | 0.28 | 0.10 | 0.0053 |
| 1-(1-enyl-palmitoyl)-GPE (P-16:0)* | Lipid | Lysoplasmalogen | 0.57 | 0.18 | **0.047** |  | 0.29 | 0.13 | **0.023** |  | 0.34 | 0.10 | **0.0012** |
| 1-(1-enyl-oleoyl)-GPE (P-18:1)* | Lipid | Lysoplasmalogen | 0.58 | 0.16 | **0.039** |  | 0.21 | 0.12 | 0.07 |  | 0.29 | 0.10 | 0.0033 |
| 10-undecenoate (11:1n1) | Lipid | Medium Chain Fatty Acid | -0.48 | 0.15 | **0.048** |  | 0.05 | 0.11 | 0.66 |  | -0.13 | 0.09 | 0.15 |
| 3-hydroxy-3-methylglutarate | Lipid | Mevalonate Metabolism | 0.80 | 0.16 | **3.20E-04** |  | 0.16 | 0.12 | 0.19 |  | 0.41 | 0.10 | **3.14E-05** |
| 1-linolenoylglycerol (18:3) | Lipid | Monoacylglycerol | -0.62 | 0.15 | **0.0088** |  |  |  |  |  |  |  |  |
| glycerophosphoinositol* | Lipid | Phospholipid Metabolism | 0.57 | 0.15 | **0.026** |  | 0.10 | 0.11 | 0.36 |  | 0.19 | 0.09 | 0.041 |
| 1-(1-enyl-palmitoyl)-2-palmitoleoyl-GPC (P-16:0/16:1)* | Lipid | Plasmalogen | 0.51 | 0.16 | **0.049** |  | -0.21 | 0.12 | 0.081 |  | 0.02 | 0.10 | 0.81 |
| pregnenolone sulfate | Lipid | Pregnenolone Steroids | -0.48 | 0.15 | **0.050** |  | -0.16 | 0.11 | 0.14 |  | -0.18 | 0.09 | 0.047 |
| pregnen-diol disulfate* | Lipid | Pregnenolone Steroids | -0.50 | 0.16 | **0.047** |  | -0.02 | 0.11 | 0.89 |  | -0.13 | 0.09 | 0.17 |
| 5alpha-pregnan-diol disulfate | Lipid | Progestin Steroids | -0.90 | 0.28 | **0.047^†^** |  | -0.10 | 0.13 | 0.43 |  | -0.17 | 0.09 | 0.072 |
| 5alpha-pregnan-3beta,20alpha-diol disulfate | Lipid | Progestin Steroids | -0.44 | 0.13 | **0.040** |  | -0.07 | 0.11 | 0.54 |  | -0.15 | 0.08 | 0.079 |
| campesterol | Lipid | Sterol | 0.97 | 0.30 | **0.047^†^** |  | -0.05 | 0.13 | 0.70 |  | 0.16 | 0.09 | 0.068 |
| 7-methylguanine | Nucleotide | Purine Metabolism, Guanine containing | 0.53 | 0.16 | **0.045** |  | -0.09 | 0.11 | 0.43 |  | 0.14 | 0.09 | 0.10 |
| N2,N2-dimethylguanosine | Nucleotide | Purine Metabolism, Guanine containing | 0.55 | 0.14 | **0.012** |  | 0.09 | 0.13 | 0.46 |  | 0.27 | 0.09 | 0.0042 |
| 2'-O-methyluridine | Nucleotide | Pyrimidine Metabolism, Uracil containing | 0.48 | 0.15 | **0.048** |  | 0.22 | 0.14 | 0.11 |  | 0.28 | 0.10 | 0.0037 |

BMI, Body mass index; CAC, Coronary artery calcium; EA, European American; LDL-C, Low-density lipoprotein cholesterol; SE, Standard error; TCA, tricarboxylic acid cycle

Model adjusted for age, sex, BMI, smoking status, hypertension status, CVD, duration of diabetes, date of plasma collection, time between plasma collection and CT exam, LDL-C, statin use

*Indicates compounds that have not been officially confirmed based on a standard, but identified by virtue of their recurrent chromatographic and spectral nature

^†^Indicates results derived from dichotomized metabolite values, i.e. presence vs absence, when >50% of the data were missing

Statistical significance (**bold**) in the Discovery cohort was defined by a False Discovery Rate (FDR) P-value (P_FDR_) ≤0.05, in the Replication cohort by a nominal P≤0.05, and in the Joint Analysis using a Bonferroni correction, i.e. 0.05/36 or P≤0.0014.
